# Pulmonary vein isolation durability and lesion regression in patients with recurrent arrhythmia after pulsed-field ablation

**DOI:** 10.1007/s10840-023-01608-7

**Published:** 2023-07-31

**Authors:** Thomas Kueffer, Anita Stefanova, Antonio Madaffari, Jens Seiler, Gregor Thalmann, Nikola Kozhuharov, Jens Maurhofer, Oskar Galuszka, Andreas Haeberlin, Fabian Noti, Helge Servatius, Hildegard Tanner, Laurent Roten, Tobias Reichlin

**Affiliations:** 1 Department of Cardiology, Inselspital, Bern University Hospital, University of Bern, Freiburgstrasse, CH-3010 Bern, Switzerland; 2 ARTORG Center for Biomedical Engineering Research, University of Bern, Bern, Switzerland

**Keywords:** Atrial fibrillation, Pulsed-field ablation, Reconnection, Pulmonary Vein isolation

## Abstract

**Background:**

A novel multipolar pulsed-field ablation (PFA) catheter has recently been introduced for pulmonary vein isolation (PVI). Pre-market data showed high rates for PVI-durability during mandatory remapping studies.

**Objective**: To present post-market data in patients with recurrent arrhythmias.

**Methods:**

Consecutive patients undergoing a redo procedure after an index PFA PVI using a bipolar-biphasic PFA system were included. 3-D electro-anatomical maps (3D-EAM) on redo procedure were compared to the 3D-EAM acquired after ablation during the index procedure. PVI durability was assessed on a per-vein and per-patient level and the sites of reconnections were identified. Furthermore, lesion extent around veins with durable isolation was compared to study lesion regression.

**Results:**

Of 341 patients treated with a PFA PVI, 29 (8.5%) underwent a left atrial redo ablation due to arrhythmia recurrence. At the end of the index procedure, 110/112 veins (98%, four common ostia) were isolated. On redo procedures performed a median of 6 months after the first ablation, 3D-EAM identified 69/110 (63%) PVs with durable isolation. In 6 (21%) patients, all PVs were durably isolated. Reconnections were more often found on the right-sided veins and on the anterior aspects of the upper veins. Only minor lesion regression was observed between the index and redo procedure (a median of 3 mm (0 – 9.5) on the posterior wall).

**Conclusion:**

In patients with arrhythmia recurrence after PFA PVI using a first-generation PFA device, durable isolation was observed in 63% of the veins and 21% of the patients showed durable isolation of all previously isolated veins.

**Graphical abstract:**

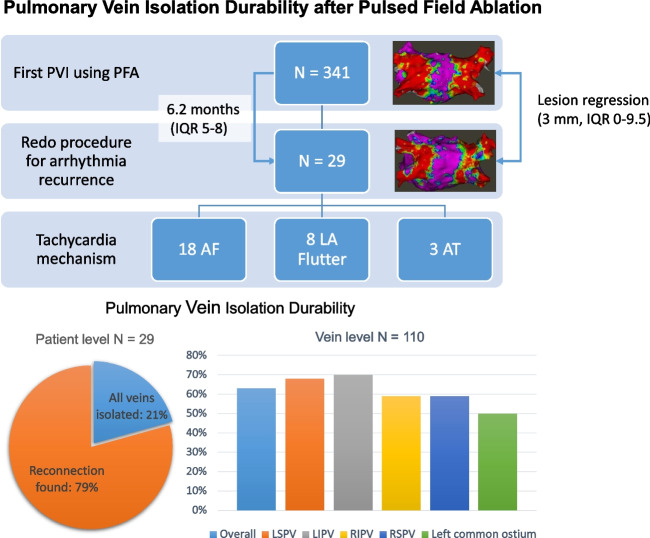

**Supplementary Information:**

The online version contains supplementary material available at 10.1007/s10840-023-01608-7.

## Introduction

Pulmonary vein isolation (PVI) is the treatment of choice in the interventional management of atrial fibrillation (AF) [1]. Thermal ablation technologies have been used for more than 2 decades. Despite significant progress in mapping and ablation technologies, the long-term efficacy in terms of rhythm control remains moderate [2]. One major reason for this is the inability to reliably achieve durable PVI. In patients undergoing repeat procedures due to arrhythmia recurrences, PV reconnections are found in 4 out of 5 patients [3].

After a surge in research and development effort in the field of cardiac electrophysiology, the first pulsed-field ablation (PFA) catheter for PVI gained CE approval in January 2021. Since then, more than 15′000 patients have been treated with the new technology and early results – mainly concerning procedural efficiency and safety of the ablation modality – look promising [4]. Unlike thermal ablation, which can cause collateral damage, PFA targets only the cardiac muscle tissue and leaves surrounding structures unharmed by applying microsecond-scale pulses that disrupt cell membranes, leading to cell death.

In contrast to thermal ablation technologies, pre-market studies using the novel PFA catheter have shown very low reconnection rates on planned remapping studies, 3 months after the index ablation [5]. This has generated high expectations that PFA could be the tool to overcome the durability issue leading to improved outcomes after AF ablation. Post-market data on PVI durability after PFA PVI however is scarce and conflicting [6, 7]. Here we present our data on PVI durability collected during redo ablations after an initial PFA PVI.

## Methods

### Study population

Consecutive patients with paroxysmal or persistent AF undergoing an index PVI at the Inselspital, Bern University Hospital, Switzerland, using the FARAPULSE PFA system (Boston Scientific, United States) were enrolled in a prospective institutional registry. The registry was approved by the local ethics committee and carried out in accordance with the declaration of Helsinki. The authors had full access to the data and bear full responsibility for its accuracy.

For this analysis, patients who underwent a repeat left atrial procedure for recurrent atrial arrhythmias after their first PFA PVI were analyzed.

### Index ablation procedure

The index PVI was performed using the PFA platform consisting of a generator, a long steerable sheath, and an ablation catheter (FARASTAR, FARADRIVE, and FARAWAVE, Boston Scientific). The generator of the platform delivers high-intensity, bipolar and biphasic electric pulses to the catheter electrodes, creating an electric field that disrupts the membranes of affected cells and leads to irreversible electroporation and cell death [8].

Prior to the procedure, patients underwent trans-esophageal echocardiography and computed tomography to exclude intracardiac thrombi and to obtain a detailed understanding of the left atrial anatomy. Deep conscious sedation using propofol and fentanyl was used, guided by a physician-led, nurse-administered protocol, while patients with a high risk of sedation complications underwent general anesthesia [9]. Left atrial access was obtained by fluoroscopy-guided transseptal puncture either using a standard transseptal sheath, followed by an exchange to the 13F Faradrive sheath, or through a direct puncture using the 13F sheath [10], depending on the physician’s preference.

In the majority of the patients, a 3D electro-anatomical mapping (3D-EAM) system (Carto 3, Biosense Webser, Irvine, CA, USA) was used at the beginning and/or at the end of the procedure to acquire a high-density 3D-EAM.

PVI was performed with a minimum of four applications in basket and four applications in flower configuration per vein (32 applications in total). In each configuration, the catheter was rotated by 36° after two applications, to cover the entire circumference. Additional applications were delivered at the discretion of the operator in subsidiary PVs (such as a right middle PV), in case of a wide carina, or if near-field signals remained after the standard ablation protocol. Voltage amplitude was changed from 1.9 kV to 2.0 kV following the recommendation of Boston Scientific in September 2021. Catheter size selection (31 vs 35 mm) was at the discretion of the operator and based on the LA size but no precise cut-off was defined.

Acute PVI was verified at the end of the procedure by 3D-EAM or by using the FARAWAVE catheter in a basket configuration with the assessment of Entrance- and Exit-Block [11]. No adenosine was used to reveal dormant conduction and no waiting time was mandated.

### Follow-up

Patients underwent 7-day Holter ECG monitoring at 3, 6, and 12 months after their ablation procedure. Recurrence was defined as any atrial tachyarrhythmia (AF, atrial flutter, or atrial tachycardia) lasting longer than 30 s between day 91 and 365 post-ablation after the standard blanking period of 90 days. In cases with recurrence, the patient and doctor discussed further options and scheduled a redo procedure upon agreement.

### Mapping protocol at repeat ablation

All repeat ablation procedures were performed using a 3D-EAM system (Carto 3, Biosense Webser, Irvine, CA, USA) and a high-density multielectrode mapping catheter (Pentarray, Biosense Webser, Irvine, CA, USA). The high-density 3D maps were used to identify reconnected veins and the individual reconnection sites and to study the extent and distribution of extra-PV left atrial (LA) scarring. Pre-ablation maps of the redo procedure were compared to post-ablation maps of the index procedure, if available.

Based on the high-density 3D maps, the target lesion set for the repeat procedure was determined and additional ablation was performed with either point-by-point radiofrequency ablation or PFA depending on the operator’s preference.

### Assessment of PV reconnections and of PFA lesion regression

Pulmonary vein reconnection was defined as any local signals and/or pace capture (Output 10 V/2 ms) remaining within a wide antral isolation area during the assessment with the multipolar mapping catheter. If local signals were present in either of the carinas, these signals were attributed to either the superior, the inferior, or both veins, depending on their exact location.

For the analysis of individual reconnection sites, a double-layer circular scheme was used: The inner circle represents ablations using the basket shape and the outer circle represents ablations using the flower configuration of the PFA catheter. Each circle was further divided into five equally spaced segments, with two of the segments pointing towards the carina (Supplemental Figure). Long left common ostia were considered as one vein.

The acquisition of post-ablation maps during the index procedure allowed us to quantify lesion regression. On each map, we measured the distance from the lesion of the right pulmonary veins to the lesion of the left pulmonary veins on three levels as follows: 1) at the height of both carinas; 2) 20 mm above this line; and 3) 20 mm below this line (Supplemental Figure). The standard voltage cutoff of < 0.05 mV was used to identify scar/ prior lesions. The difference in lesion distance was calculated and averaged to represent overall lesion regression. Patients with PV reconnection on the posterior side were excluded from this analysis, as lesion regression could not be measured reliably.

### Statistical analysis

Continuous data are shown as mean (± standard deviation) or as median (interquartile range) as appropriate and compared by a Mann–Whitney-U test for non-normal distributions and by a t-test for normal distributions. Categorical variables were reported as counts (percentage) and compared by a Pearson chi-square test or Fisher exact test. The statistical analysis was conducted using R 4.2.2 (R Core Team, Vienna, Austria).

## Results

### Patient population and details on index PVI procedures

From Mai 2021 to October 2022, 341 patients underwent an index PVI using PFA (23% females; paroxysmal AF n = 173 (51%); persistent AF n = 168 (49%). Baseline characteristics and procedural data are presented in Tables 1 and 2. A 31 mm device was used in 318 (93%) patients, and a 35 mm in 23 (7%). High-density 3D-EAM was used in 290 (85%) patients for the confirmation of PVI. At the end of the first procedure, 1301 of 1304 (99.8%) of PV’s were isolated (One RIPV was not isolated after a cardiac tamponade preventing the continuation of the procedure and two LIPV could not be isolated despite extensive ablation with different device configurations). All veins were isolated using PFA only and no touch-up using radiofrequency ablation was performed.

**Table 1:** Data at first PVI

Variable	Overall	With redo	Without redo	*p*-value
*N*	341	29	312	
Age, years	68.0 [60.0, 74.0]	69.0 [67.0, 73.0]	67.0 [59.0, 74.0]	0.587
Male sex — no. (%)	263 (77.1)	23 (79.3)	240 (76.9)	0.951
Persistent AF	185 (54.3)	24 (82.8)	161 (51.6)	0.002
Body-mass index	27.3 [24.4, 31.1]	28.2 [25.5, 31.5]	27.2 [24.2, 31.1]	0.303
CHA_2_DS_2_-VASc score — no. (%)				0.208
0	57 (16.7)	3 (10.3)	54 (17.3)	
1	74 (21.7)	3 (10.3)	71 (22.8)	
2	87 (25.5)	11 (37.9)	76 (24.4)	
3	75 (22.0)	5 (17.2)	70 (22.4)	
4	34 (10.0)	5 (17.2)	29 (9.3)	
> 4	14 (4.1)	2 (6.9)	12 (3.8)	
NYHA classification — no. (%)				0.136
0	26 (7.6)	1 (3.4)	25 (8.0)	
1	147 (43.1)	10 (34.5)	137 (43.9)	
2	137 (40.2)	12 (41.4)	125 (40.1)	
3	29 (8.5)	6 (20.7)	23 (7.4)	
4	2 (0.6)	0 (0.0)	2 (0.6)	
Previous stroke / TIA — no. (%)	23 (6.7)	3 (10.3)	20 (6.4)	0.674
Previous myocardial infarction — no. (%)	32 (9.4)	4 (13.8)	28 (9.0)	0.604
Coronary artery disease — no. (%)	63 (18.5)	6 (20.7)	57 (18.3)	0.943
Hypertension — no. (%)	192 (56.3)	16 (55.2)	176 (56.4)	1.000
Diabetes mellitus — no. (%)	47 (13.8)	5 (17.2)	42 (13.5)	0.777
Chronic obstructive pulmonary disease — no. (%)	15 (4.4)	3 (10.3)	12 (3.8)	0.246
Obstructive sleep apnea syndrome — no. (%)	58 (17.0)	7 (24.1)	51 (16.3)	0.418
Beta-blocker — no. (%)	240 (70.4)	24 (82.8)	216 (69.2)	0.189
Class I AAD — no. (%)	20 (5.9)	2 (6.9)	18 (5.8)	1.000
Class III AAD — no. (%)	129 (37.8)	13 (44.8)	116 (37.2)	0.540
Left atrial diameter — mm	43.0 [38.0, 48.0]	46.5 [41.0, 50.5]	43.0 [38.0, 48.0]	0.072
Left atrial volume index — mL/m^2^	42.0 [35.0, 52.0]	48.0 [43.5, 56.0]	40.0 [34.0, 49.0]	0.015
Left ventricular ejection fraction – no. (%)	60.0 [50.0, 60.0]	55.0 [50.0, 60.0]	60.0 [50.0, 60.0]	0.124
Left common ostium — no (%)	60 (17.6)	5 (17.2)	55 (17.6)	1.000

Numbers are median [IQR] unless otherwise noted. *IQR* interquartile range

**Table 2:** Procedural data at first PVI

Variable	Overall	With redo	Without redo	*p*-value
Procedure time of index procedure	90.0 [71.0, 116.0]	101.0 [80.0, 138.0]	88.5 [71.0, 115.2]	0.079
Number of PFA applications	36.0 [32.0, 40.0]	34.0 [32.0, 44.0]	36.0 [32.0, 40.0]	0.877
More than 32 PFA applications — no (%)	192 (56.3)	16 (55.2)	176 (56.4)	1.000
Pulse Amplitude 1.9 kV — no (%)	48 (14.1)	9 (31.0)	39 (12.5)	0.014
Catheter size 35 mm — no (%)	23 (6.7)	5 (17.2)	18 (5.8)	0.049
PVI confirmation using 3D-mapping — no (%)	290 (85.3)	27 (93.1)	263 (84.6)	0.333
Fluoroscopy time — min	22.0 [15.6, 29.0]	24.5 [18.6, 29.8]	22.0 [15.3, 28.9]	0.191
Fluoroscopy dose — Gycm^2^	6.1 [3.1, 11.2]	7.4 [3.6, 14.4]	6.0 [3.0, 10.9]	0.195

Numbers are median [IQR] unless otherwise noted. *IQR* interquartile range

### Recurrence of atrial arrhythmias during Follow-up

After a median of 9 (6–14) months after the first ablation, recurrence of atrial arrhythmias occurred in 58/341 (17%) patients (8/155 (5.2%) with paroxysmal AF and 50/186 (27%) with persistent AF).

**Fig. 1:**
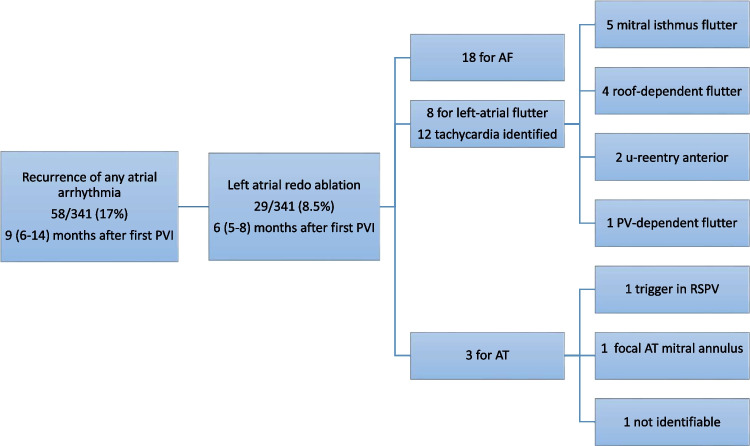
Study flowchart and recurring arrhythmias. 29 Redo procedures were performed after an index pulmonary vein isolation using pulsed-field ablation, 18 for recurrence of atrial fibrillation, 8 for left atrial flutter, and 3 for atrial tachycardia. AF = atrial fibrillation; AT = Atrial tachycardia; PV = Pulmonary vein; PVI = Pulmonary vein isolation; RSPV = Right superior pulmonary vein

A repeat LA ablation procedure was performed in 29/341 (8.5%) patients with a median of 6.2 (5.1—8.3) months after the first ablation (4/155, 2.6% in paroxysmal AF; 25/168, 13% in persistent AF). The recurring arrhythmias were AF in 18 (62%), atypical left atrial flutter in 8 (28%) and focal left atrial tachycardia in 3 (10%) (Fig. 1).

### Findings on redo procedures

Baseline characteristics and procedural characteristics of the index PVI are shown in Tables 1 and 2. Predictors for a redo ablation were persistent AF, high left atrial volume index, use of a voltage amplitude of 1.9 kV for PFA PVI, and the use of a 35 mm device.

**Fig. 2:**
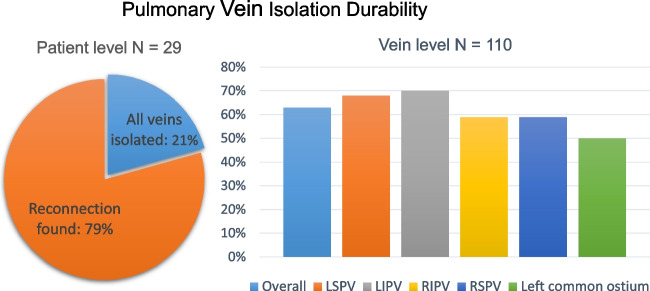
Pulmonary vein isolation durability at redo procedure after PFA PVI. Proportion of patients with all veins isolated and persisting isolation of pulmonary veins found during redo procedure after a first pulmonary vein isolation using pulsed-field ablation. LSPV = Left superior pulmonary vein; LIPV = Left inferior pulmonary vein; RIPV = Right inferior pulmonary vein; RSPV = Right superior pulmonary vein

Mapping during the 29 redo procedures identified 110 previously isolated veins (4 long common left ostia, supplemental data). Durable isolation was found in 69/110 (63%) pulmonary veins overall. PVI was persistent in 17/29 (59%) RSPV, 17/29 (59%) RIPV, 17/25 (68%) LSPV, 16/23 (70%) LIPV, and 2/4 (50%) long left common ostia (Fig. 2). Persisting isolation of all previously isolated PV’s was found in 6 of 29 (21%) patients. Reconnection was not different with different catheter sizes: we found 5/18 (28%) reconnections for the 35 mm device and 35/92 (38%) reconnections for the 31 mm device, p = 0.575. All post-ablation maps of the first procedure and pre-ablation maps of the repeat procedure can be found in the supplemental data.

**Fig. 3:**
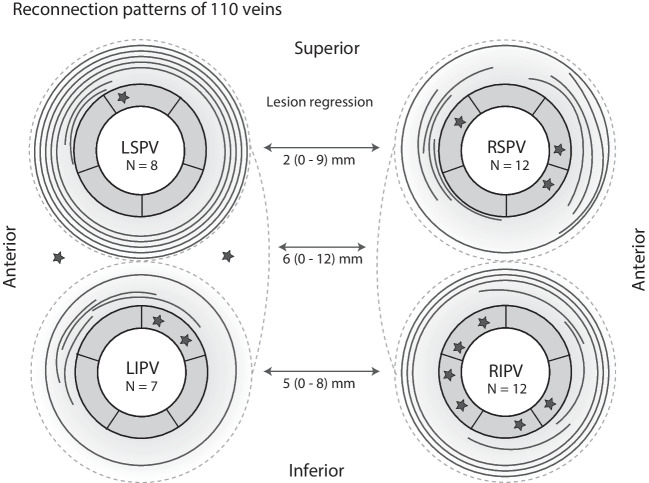
Reconnection patterns identified by high-density mapping during repeat procedures after first pulmonary vein isolation using pulsed-field ablation. Stars denote focal gaps. Full circles denote complete, un-localizable reconnection of the pulmonary vein. Curved lines denote segmental gaps. Two additional stars denote the reconnection of 2 long left common ostia. LIPV = left inferior pulmonary vein; LSPV = left superior pulmonary vein; RIPV = right inferior pulmonary vein; RSPV = right superior pulmonary vein

Identified reconnection sites were located on the anterior aspects of the upper PV’s (8, 20%), on the inferior aspect of the RIPV (6, 14%), on the posterior aspect of the RSPV (5, 12%), and on the anterior carina of the LIPV (4, 10%). In patients with post-ablation 3D-EAM available from the index procedure (n = 27), lesion regression at the posterior wall was 3 mm (IQR 0 – 9.5) overall. In more detail, it was 6 mm (IQR 0—12) at the level of the carinas, 2 mm (IQR 0—9) at the upper line and 5 mm (IQR 0—8) at the lower line (Fig. 3).

**Fig. 4:**
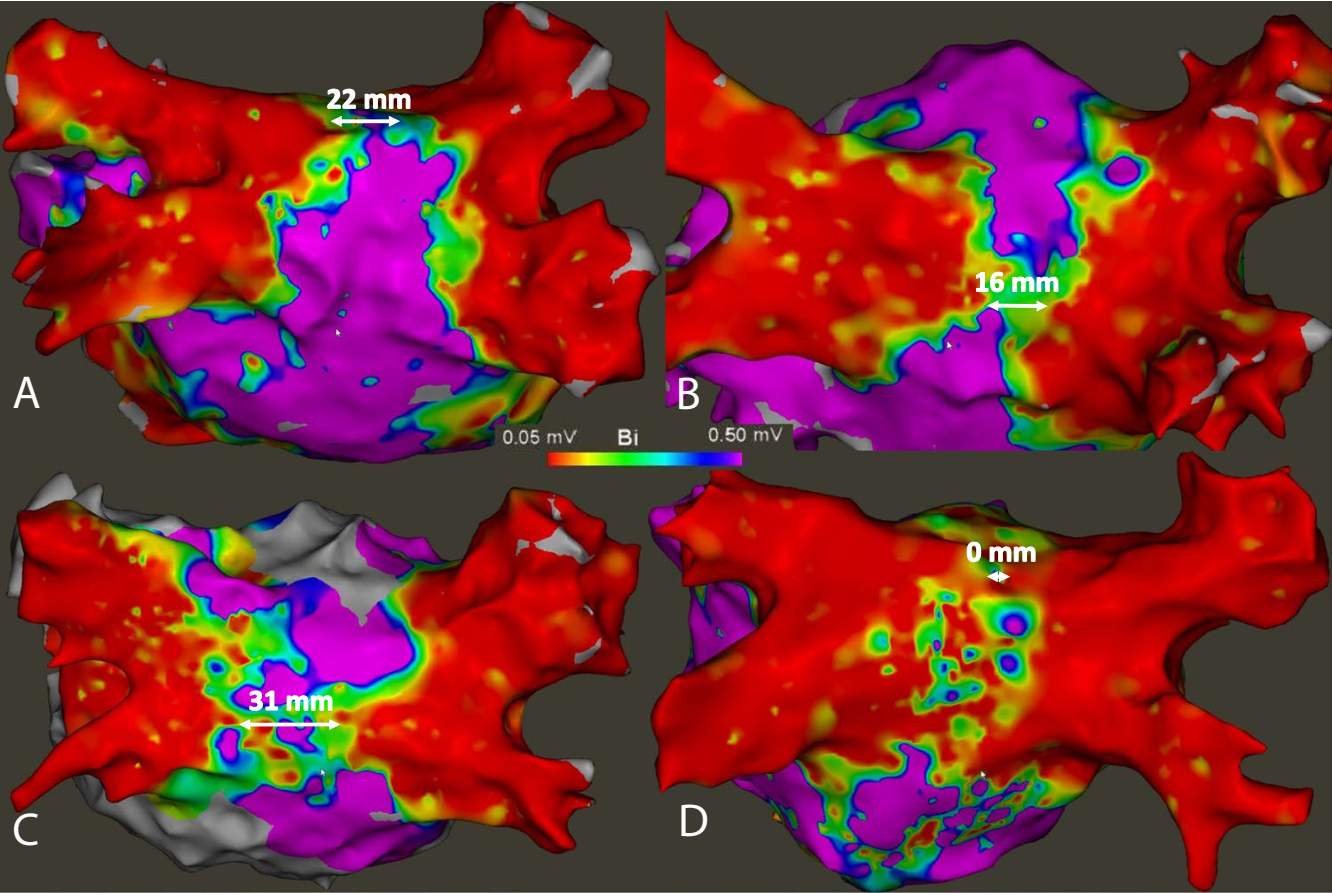
Voltage maps in patients with subsequent roof-dependent atrial flutter. 3D-electroanatomical bipolar voltage maps acquired at the end of the first PFA PVI procedure in 4 patients (Panels **A**-**D**). Minimal channel width on the posterior wall is indicated. During the redo procedures, roof-dependent left atrial flutter was the confirmed tachycardia mechanism in all 4 patients. In addition to a narrow remaining channel, scarring of the posterior wall was present in patients **C** and **D**. Color scale: Bipolar voltage (0.05 mV … 0.5 mV)

In patients with recurrence of atrial flutter, 12 tachycardias could be identified. The tachycardia mechanism was mitral isthmus dependent flutter in 5 (42%), roof-dependent flutter in 4 (33%) patients, anterior micro-reentry in 2 (17%), and peri-ostial PV flutter in 1 (8.3%) case. In cases with roof-dependent flutter, the minimal channel width of the critical isthmus on the post-ablation map of the first procedure was 19 (12 – 24) mm (Fig. 4). In patients with recurrence of AT, the tachycardia mechanism was a trigger in a reconnected RSPV in one patient, a focal AT at the mitral annulus in another, and the mechanism not identifiable in the third.

The ablation modality used during the repeat ablation was PFA in 22 (76%) patients and point-by-point radiofrequency ablation in 7 (24%). Posterior wall ablation was added in 18 (62%) patients (all with persistent AF).

## Discussion

In 29 patients with arrhythmia recurrence after PFA PVI undergoing a repeat ablation procedure a median of 6 months (IQR 5–8) after the first ablation, we report the following main findings: First, PV reconnection does occur after PVI with this first-generation PFA device. Despite verified PVI by 3D-EAM at the end of the index procedure, PV reconnections occurred in 37% of the veins and in 79% of the patients with arrhythmia recurrence. Second, PV reconnections occurred more frequently in the right-sided pulmonary veins and reconnection sites were located at the anterior aspects of the upper veins and, on the inferior aspect of the RIPV, on the posterior side of the RSPV, and on the anterior carina of the left inferior vein. Third, lesion regression around durably isolated veins was minimal, with a median of 3 (0 – 9.5) mm at the posterior wall.

### Durability of PV isolation

Durable PVI is a critical and still unmet need in electrophysiology. PVI durability can be assessed within study protocols in all patients regardless of arrhythmia recurrence, or in clinical routine in the subset of patients with recurrent arrhythmias.

In studies using thermal energies and with mandatory repeat procedures in all patients, durability of PVI was reported in 80% of the veins, and roughly 50% of all patients showed no reconnections [12]. A pre-market study performed with mandatory repeat procedures in 44 patients showed a remarkable increase in PVI durability to 96% of the veins, and 84% of the patients had no PV reconnections [13]. These data created enthusiasm and high expectations that PFA eventually could be the tool to provide durable PVI in the vast majority of patients.

Because of the negative selection of patients with clinically indicated repeat procedures due to arrhythmia recurrences, reconnections can be expected to be observed more frequently in such patient populations. Recent studies of repeat ablations from multi-center studies after index PVIs using thermal ablation technologies reported durabilities of only 46—64% on a per vein level and only 10—30% on a patient level [3, 14]. Post-market durability data after index PFA PVI was first reported by the Frankfurt group. In 25 patients with arrhythmia recurrence, they found durable isolation in 90% of the veins and durable isolation of all four veins in 76% of the patients a median of 6 months after PFA PVI [6]. These data were in line with the pre-market PFA data [13] and indicate superiority in terms of PVI durability compared to data from studies investigating thermal energies. A recent small report from Copenhagen with 8 patients however reported durable PVI in only 38% of redo procedures after PFA PVI [7]. Our findings are similar with durable PV isolation in 62% of the veins and 21% of the patients.

Several aspects might contribute to the observed differences: First, while the learning curve for the Farapulse system in general, is considered to be short, proper catheter and sheath positioning as well as adequate tissue contact are important. Second, there may be methodological differences in the assessment and definition of PV reconnections across the studies. Third, the distribution in the use of the 31 mm vs. 35 mm devices, and energy choice of 1.9 kV vs. 2.0 kV was different and might have contributed to the different results. Fourth, given that the sample sizes are still small, anatomical factors and chance might have played a role as well. Additional data from other centers will help to elucidate the real-world durability of PFA PVI.

### Pattern of reconnection sites

Reconnections were more often found on the right-sided veins and on the anterior aspects of the upper veins. Stable placement of the catheter in the right-sided PVs may be somewhat more difficult than in the left PVs and is a known issue in cryoballoon ablation [15]. Anterior torque on the sheath is important to secure sufficient contact. Furthermore, proper placement of the septal and inferior petals of the PFA catheter in the flower configuration can be challenging with the right-sided PVs. Failure to cover the septal aspect of the right PVs might have contributed to the observed reconnections.

### Lesion evolution from the index to the repeat ablation

Surrounding the core zone of irreversible electroporation, a border zone of reversible electroporation may be created with PFA applications. This border is electrically inactivated acutely, but prone to recovery over time. In our population, lesion regression on the posterior wall was minimal with 3 mm (IQR 0 – 9.5) between the index procedure and the redo procedure. This finding is comparable to remapping studies conducted during the approval study of the FARAPULSE system where Kawamura et al. found a mean regression on the posterior wall by 1.15 mm [16].

### Clinical implications

Our study highlights the importance of procedural aspects including careful device positioning and sufficient tissue contact. Sensor-enabled integration of the PFA device into a 3D-Mapping system will be useful in this regard, but will only be possible with the second generation of the PFA device used in our study [17, 18]. Two other PFA systems with full integration of the PFA devices into a 3D-Mapping system have completed their pivotal studies and are expected to receive regulatory approval soon [19, 20]. The potential of integrated systems to further improve PVI durability will have to be assessed in the future. In addition, 3D-Mapping integration will allow direct identification and treatment of left atrial scar tissue outside the PVs [21]. For example, pre-existing scar on the posterior wall (particularly in patients with persistent AF), or narrow channels of healthy tissue created by the PFA applications that can subsequently cause roof-dependent flutter could be visualized and treated as necessary. Indeed, in our cases with recurrence of roof-dependent flutter, the maps acquired at the end of the PFA procedure showed a rather narrow channel or scarring on the posterior wall (Fig. 4). In such cases, preemptive ablation of the posterior wall may be warranted to prevent successive flutter. Posterior wall ablation can be performed with the current device in a safe and efficient manner [18, 22]. In addition, in patients with all pulmonary veins durably isolated (37% of our population), the recurrence of arrhythmia is likely due to extra-PV triggers. The optimal treatment of this important group of patients is unknown at this point in time. Whether empiric posterior wall ablation could be helpful in these patients remains to be investigated. Finally, the use of a 35 mm catheter and the use of a voltage amplitude of 1.9 kV were both associated with a redo procedure in our preliminary analysis, both of which should be considered during procedure planning. In a similar study, Tohoku et al. found a higher reconnection rate in patients with PVI using the 35 mm catheter. One possible factor could be reduced ablation effectiveness by spreading the same electrical field over a larger area. However, we used the 35 mm device more often in the beginning, in conjunction with an ablation voltage of 1.9 kV, and more often in enlarged atria. Both, low ablation voltage, and a large atrium are confounders in our analysis. In addition, an early ablation results in a longer follow-up period, therefore increasing the probability of a redo procedure. In addition, we did not find a difference in PV reconnection rates for the two catheters. Ablation using 2.0 kV has been recommended by the catheter manufacturer.

### Limitations

Potential limitations of the present study merit consideration. First, this is a retrospective observational single-center study. Our results have to be interpreted in conjunction with similar data reported from other groups. Second, only 29/58 (50%) patients with recurrence of an arrhythmia underwent repeat ablation. This might have introduced a selection bias. Third, the definition of PV reconnection and the identification of reconnection sites can be difficult in ambiguous cases and the subjective judgement may vary. However, the applied methodology of high-density bipolar mapping (median number of mapping points during redo procedures was 2881 (IQR 1912 – 6091) was rigorous for identifying lesion gaps and the risks for misinterpretation are low. To allow comparison with other groups, we have disclosed all maps of both index and redo procedures in the supplement. Last, lesion regression might be challenging to quantify for a number of reasons: the quality of the maps acquired at the different procedures might be different regarding the spatial density of measurement points, tissue contact, cardiac rhythm, and overall completeness. Further, reconnected veins impoverish the validity of lesion regression analysis. In order to minimize these aspects, we only measured lesion regression on high-quality maps without PV reconnections on the posterior side of the veins.

## Conclusion

In patients with arrhythmia recurrence after PFA PVI using a first-generation PFA device, durable isolation was observed in 63% of the veins and 21% of the patients showed durable isolation of all previously isolated veins.

## Clinical perspectives

- In patients with recurrent atrial arrhythmias after PVI using PFA, durable PVI was observed in 63% of the veins and in 21% of the patients. This underlines the importance of the still unmet need of durable PVI for rhythm control and needs further investigation and comparison to thermal ablation technologies.
- Further, the study highlights the importance of catheter positioning and tissue contact for pulsed-field ablation. In this regard, sensor-enabled integration of the PFA device will be useful but only available with 2^nd^ generation systems.
- The use of a 35 mm catheter and the use of an ablation voltage of 1.9 kV were both associated with a redo procedure and should be avoided, if possible.

### Supplementary Information

Below is the link to the electronic supplementary material.

Supplementary file1 (DOCX 181 KB)

Supplementary file2 (DOCX 4.91 MB)

## Data Availability

The data underlying this article will be shared on reasonable request to the corresponding author.
