# Supplementary material for: Pulmonary vein isolation durability and lesion regression in patients with recurrent arrhythmia after pulsed-field ablation
Source: J Interv Card Electrophysiol. 2023 Jul 31;67(3):503–11. doi: 10.1007/s10840-023-01608-7 (PMC11015999; doi:10.1007/s10840-023-01608-7)
Supplement: Supplementary file 1 — (DOCX 181 KB) [file 10840_2023_1608_MOESM1_ESM.docx]

### Supplemental Figure

###
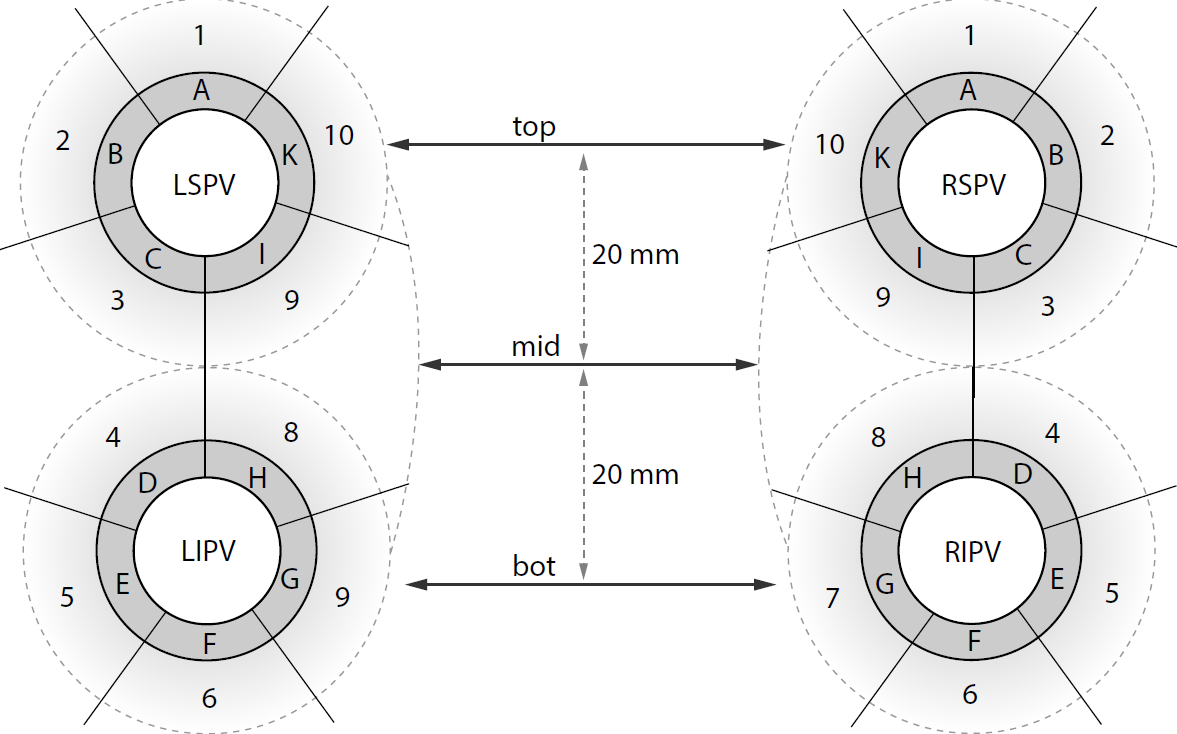


### Supplemental Figure

Scheme for identification of pulmonary vein reconnection sites and quantification of posterior wall channel width. The inner circle corresponds to ablations with the catheter in basket configuration, the outer circle to ablations with the catheter in flower configuration. LIPV = left inferior pulmonary vein; LSPV = left superior pulmonary vein; RIPV = right inferior pulmonary vein; RSPV = right superior pulmonary vein.

**Supplemental data**

Illustration of all 27 post-ablation maps acquired during the index procedure and all 29 pre-ablation maps acquired during the redo procedure.
