# Supplementary material for: Pulmonary vein isolation durability and lesion regression in patients with recurrent arrhythmia after pulsed-field ablation
Source: J Interv Card Electrophysiol. 2023 Jul 31;67(3):503–11. doi: 10.1007/s10840-023-01608-7 (PMC11015999; doi:10.1007/s10840-023-01608-7)

Supplemental Material

**Pulmonary vein reconnection rates and lesion regression during repeat procedures in patients with recurrent arrhythmia after pulsed field ablation pulmonary vein isolation**

Thomas Kueffer MSc^1^, Antonio Madaffari MD^1^, Aline Mühl MSc^1^, Jens Seiler MD^1^, Gregor Thalmann MD, Helge Servatius MD^1^, Nikola Kozhuharov MD^1^, Hildegard Tanner MD^1^, Andreas Haeberlin MD,PhD^1,2^ , Fabian Noti MD^1^, Samuel H. Baldinger MD^1^, Laurent Roten MD^1^, Tobias Reichlin MD

Page 2 – 16: Side to side comparison of post-ablation maps of the index PVI procedure performed using PFA to the pre-ablation maps of the redo procedure in 29 patients.


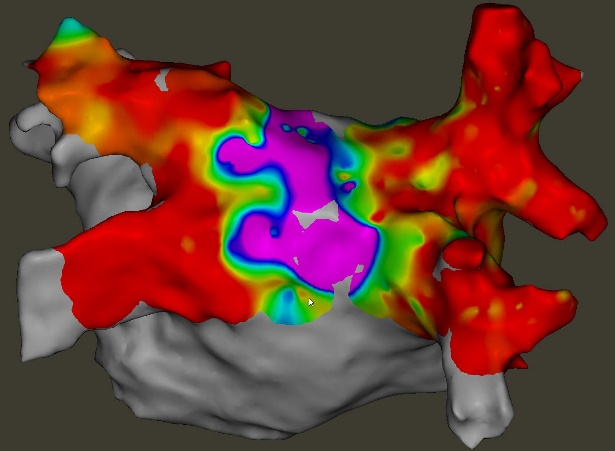

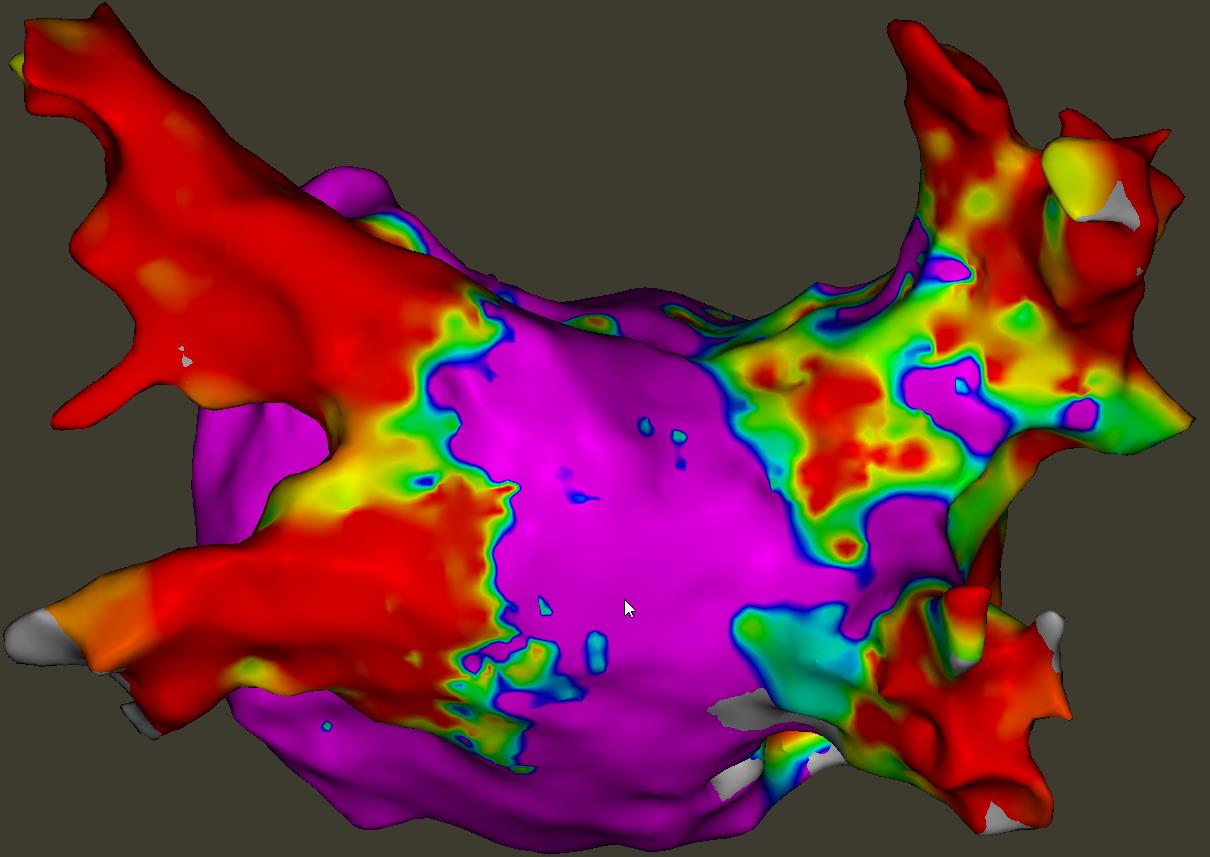


Patient #1

Map after first PFA procedure Map at repeat procedure


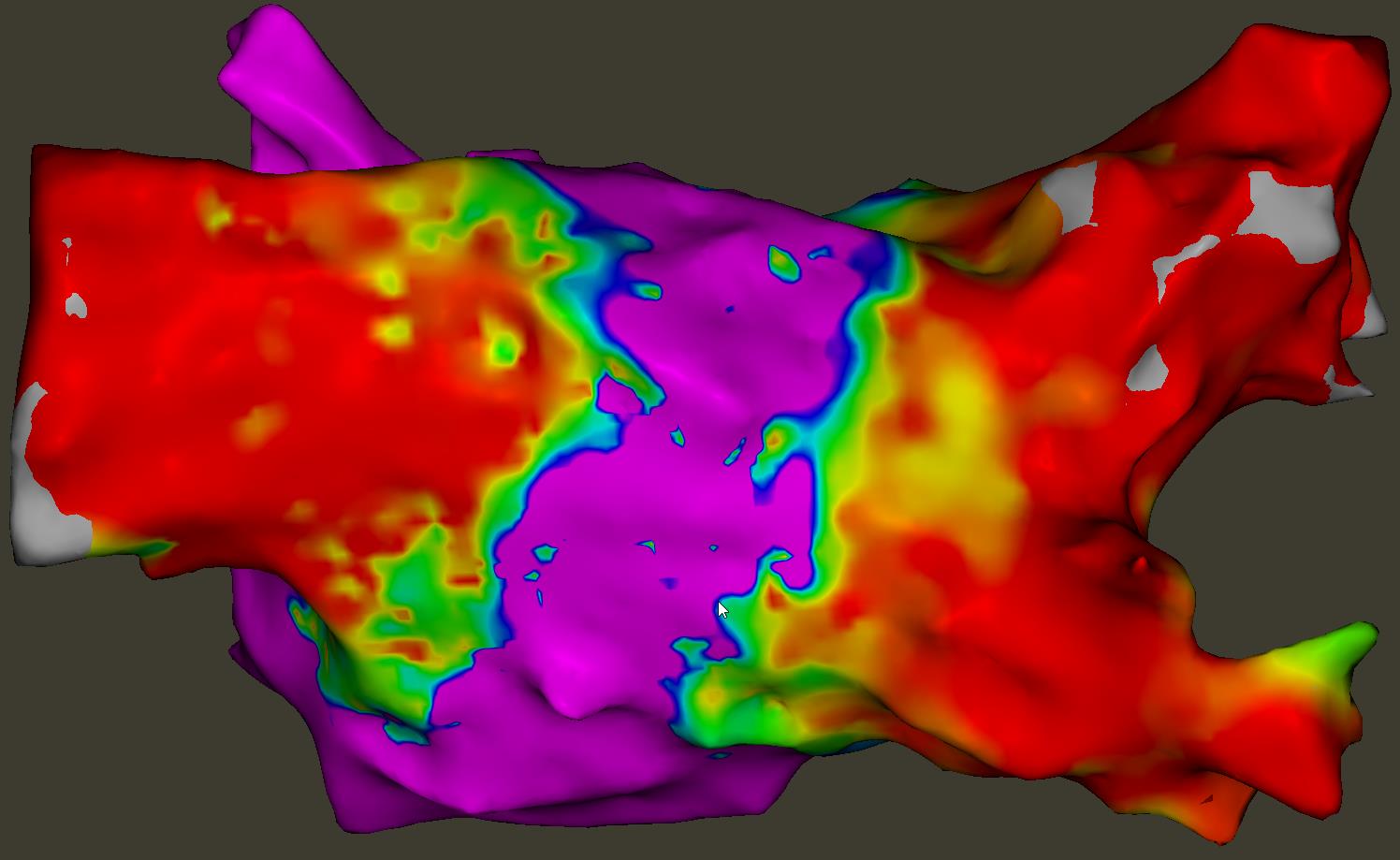

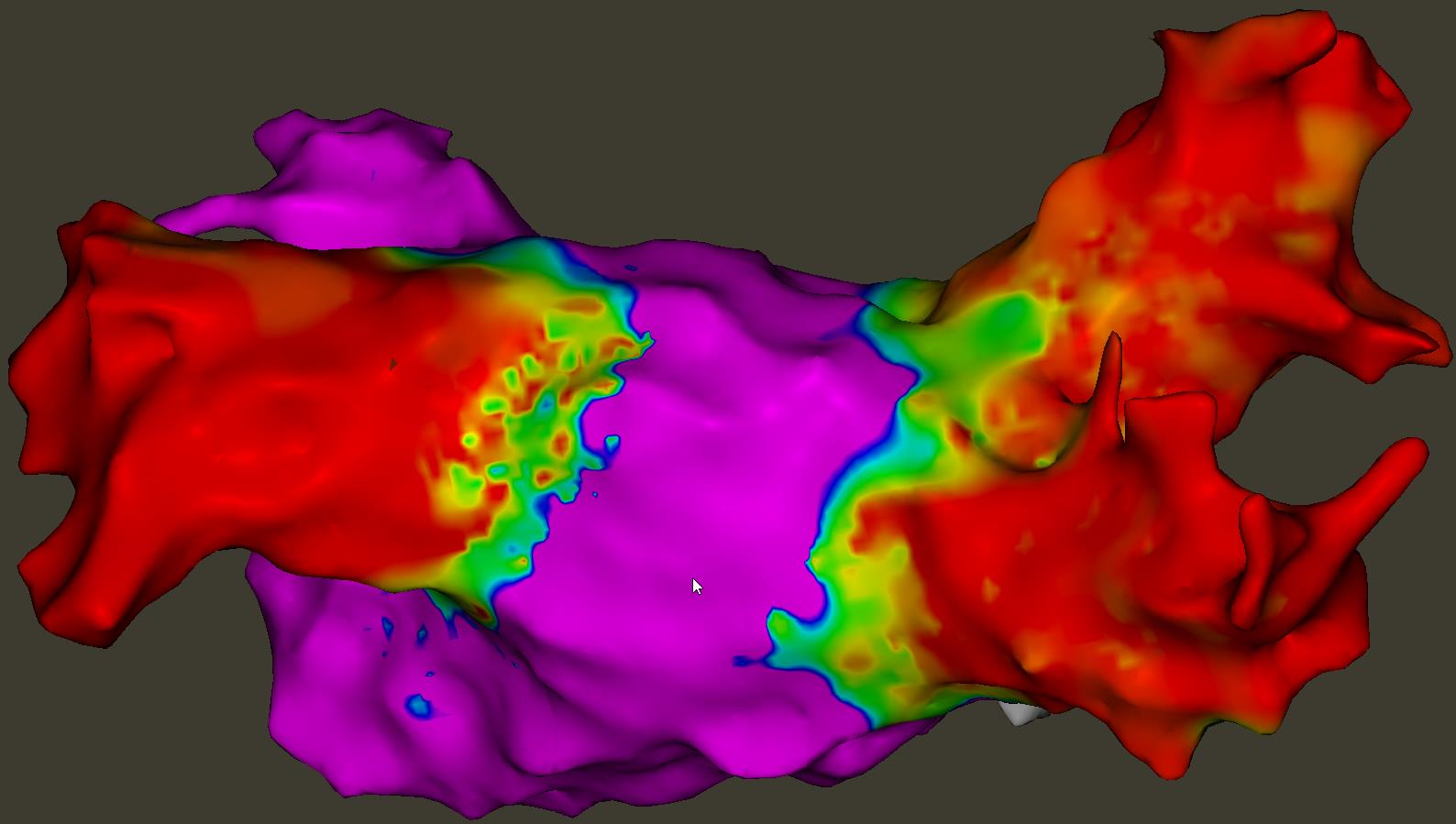


Patient #2


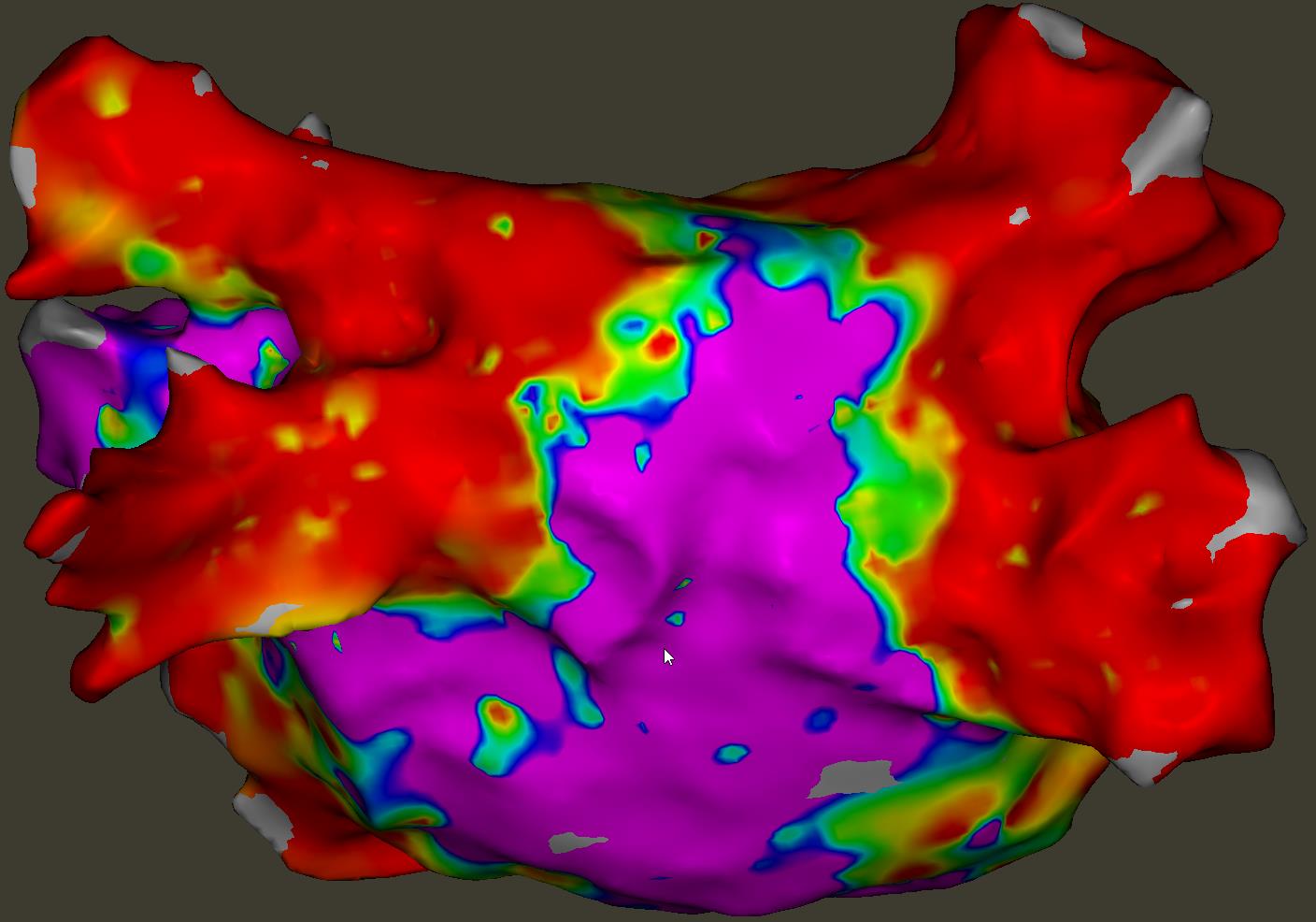

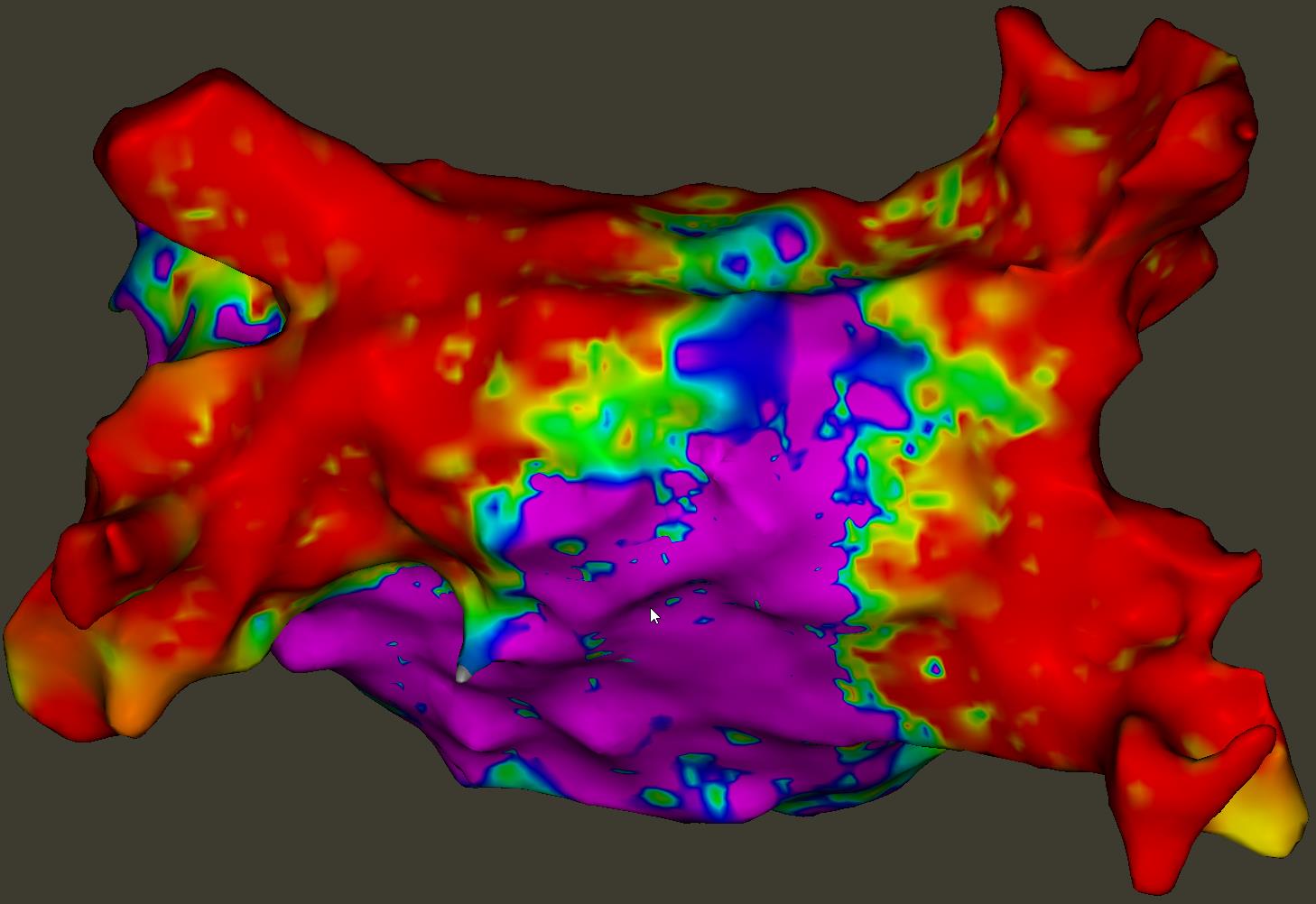

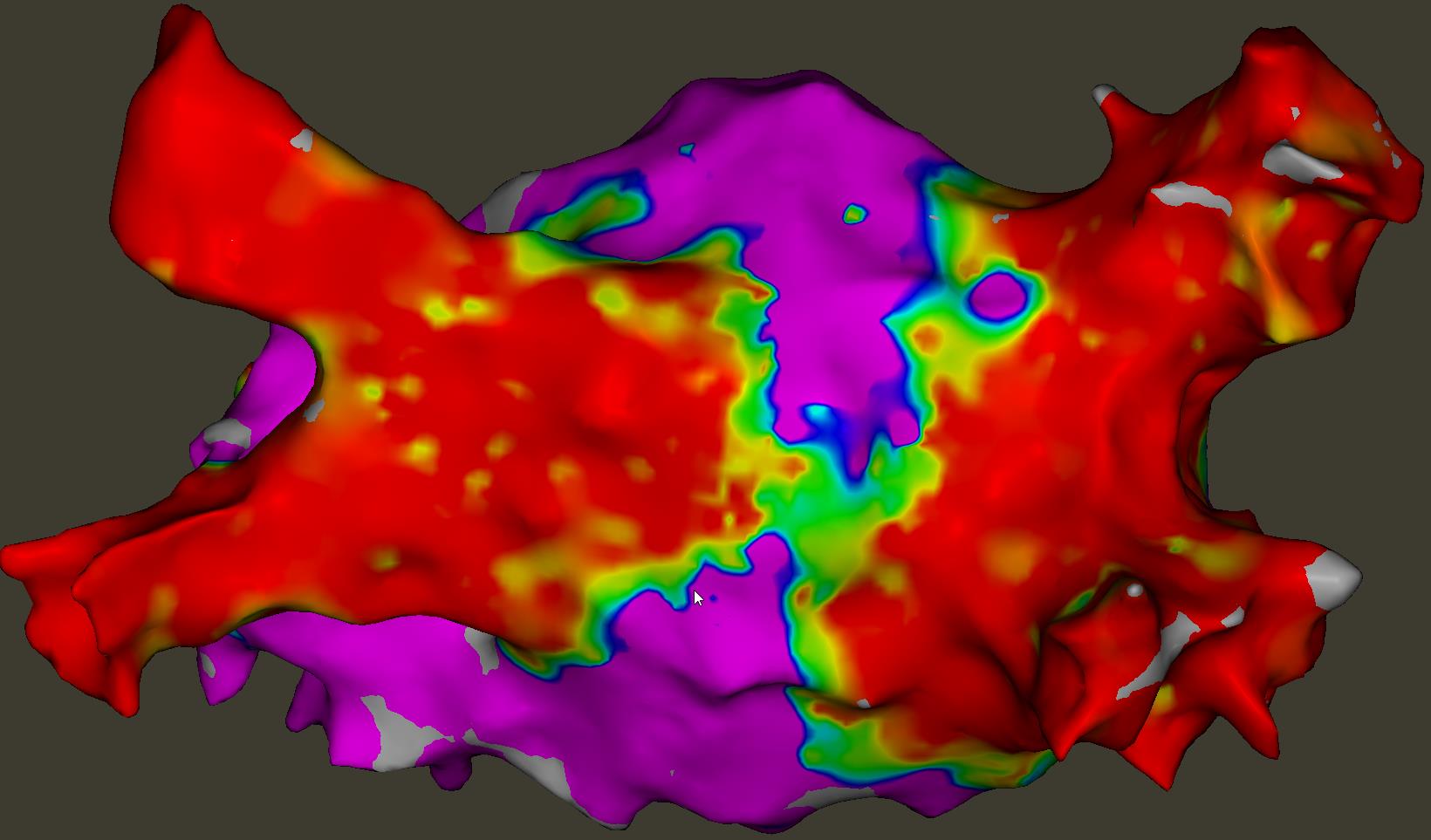

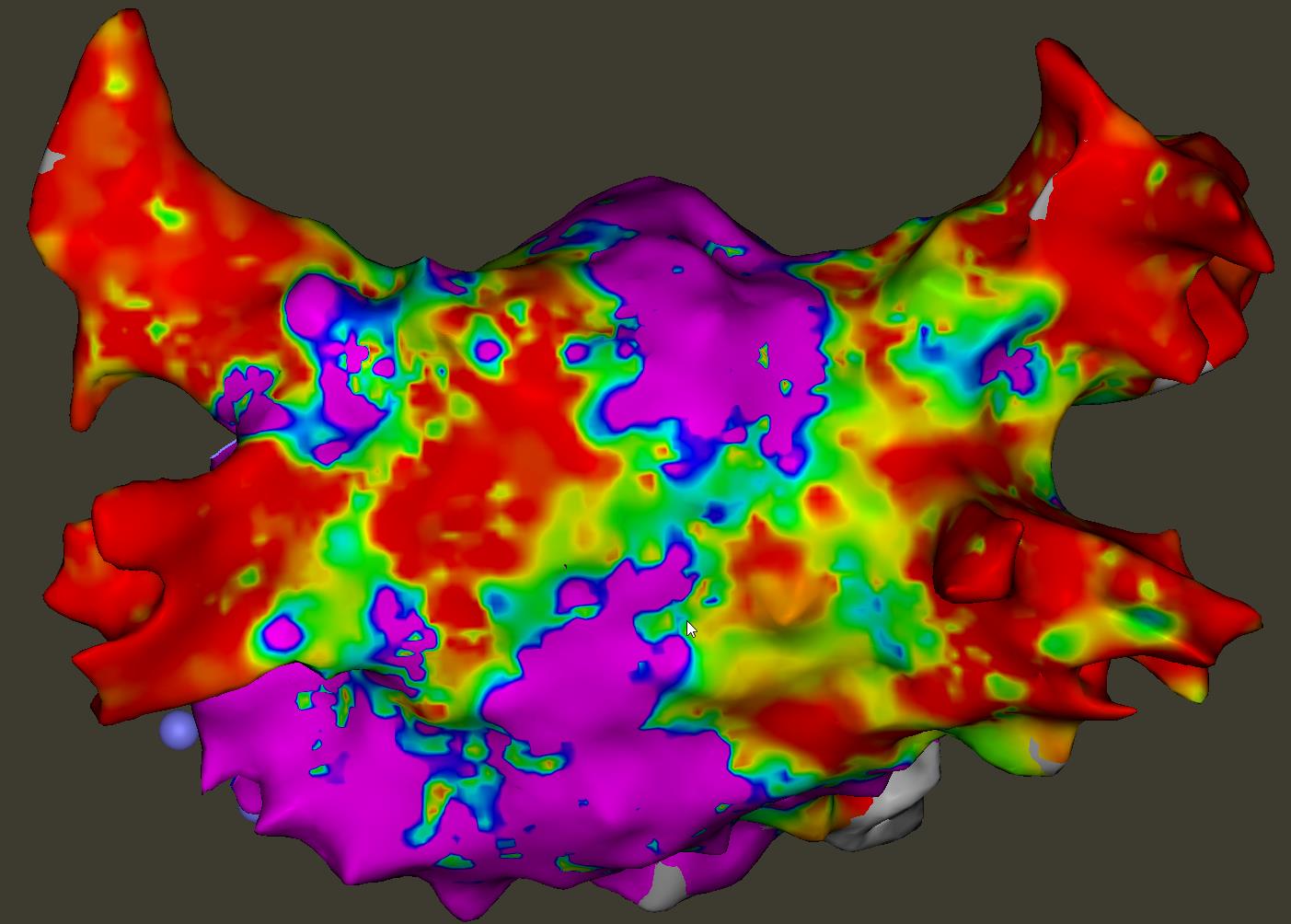

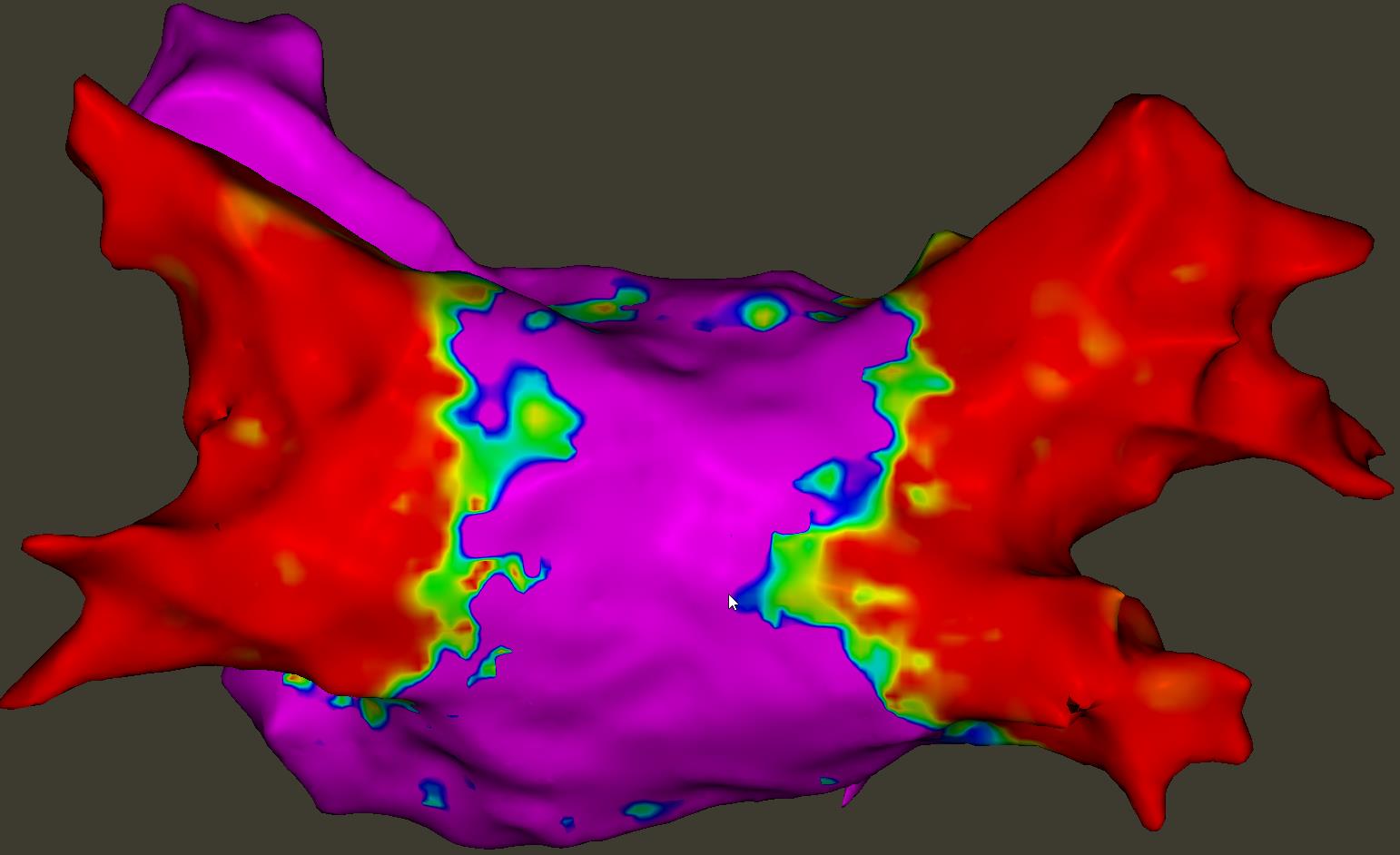

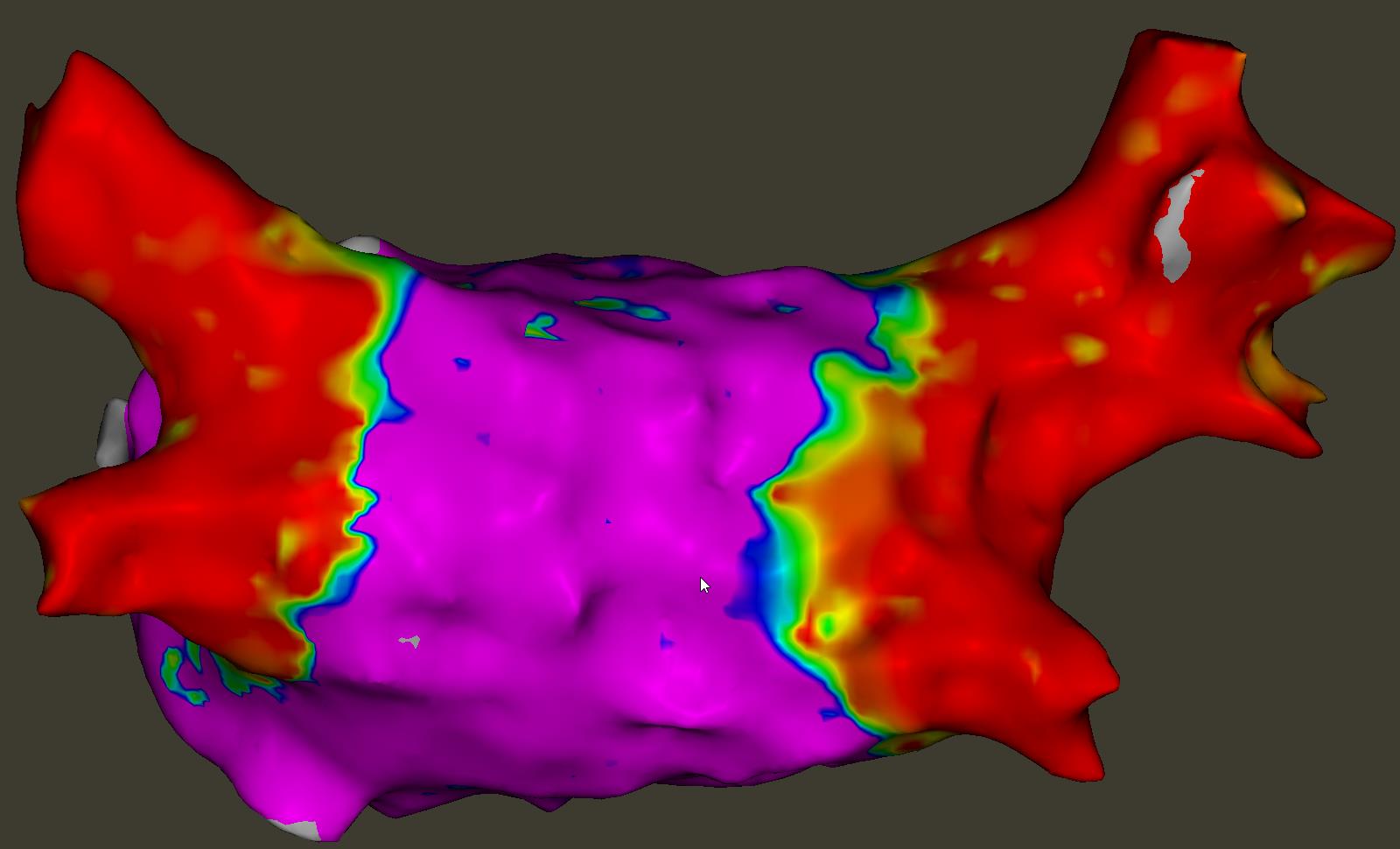

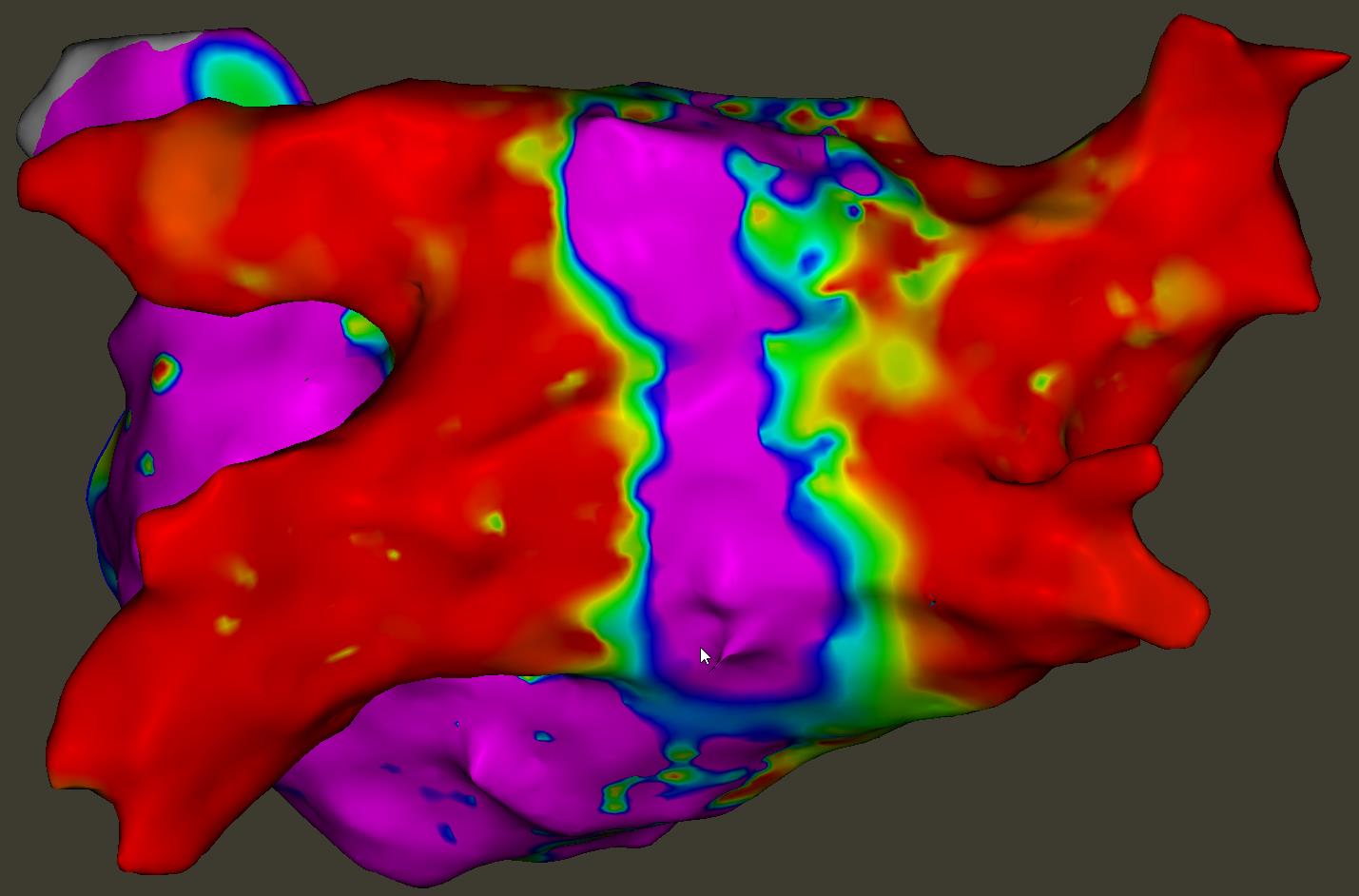

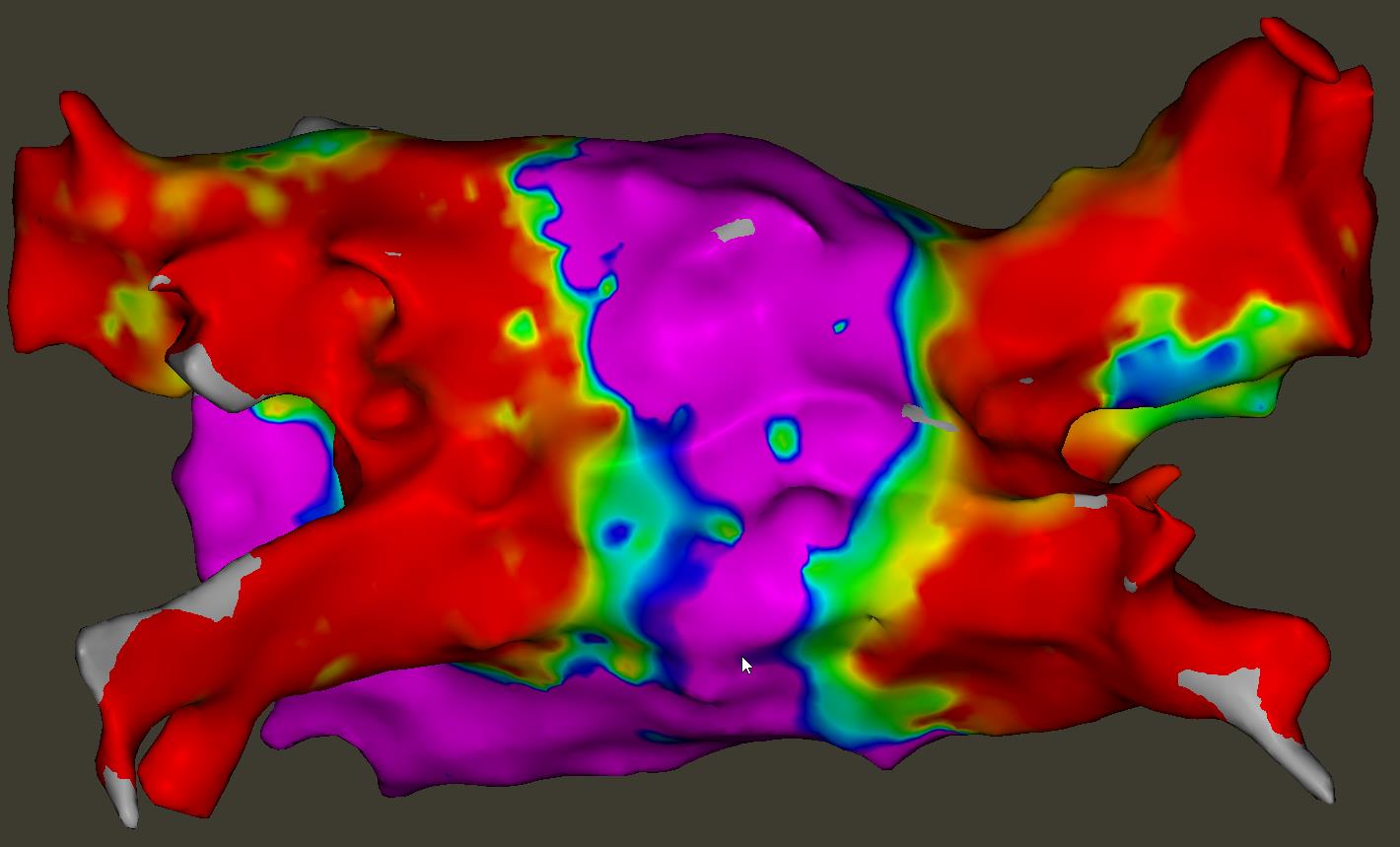

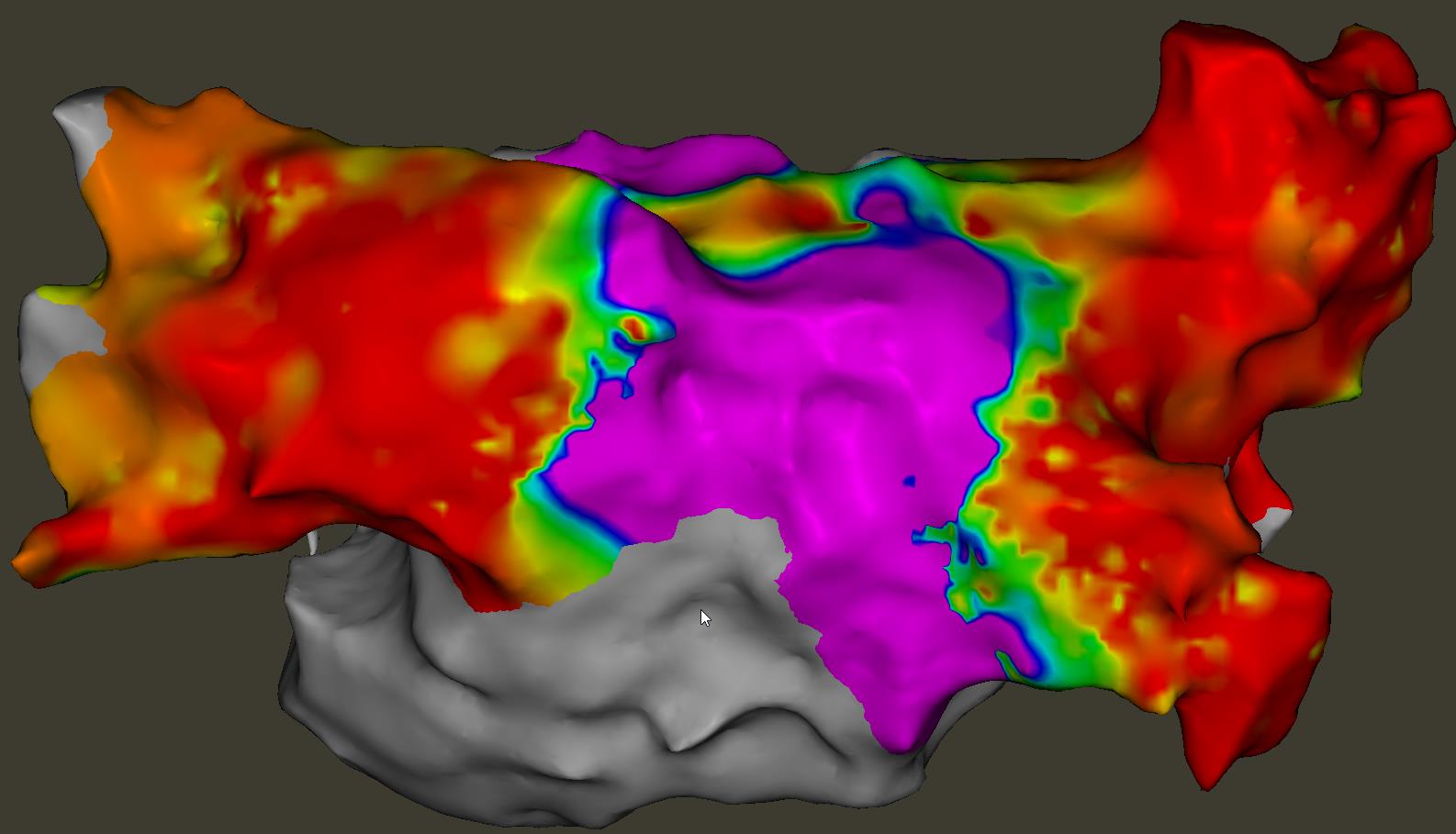

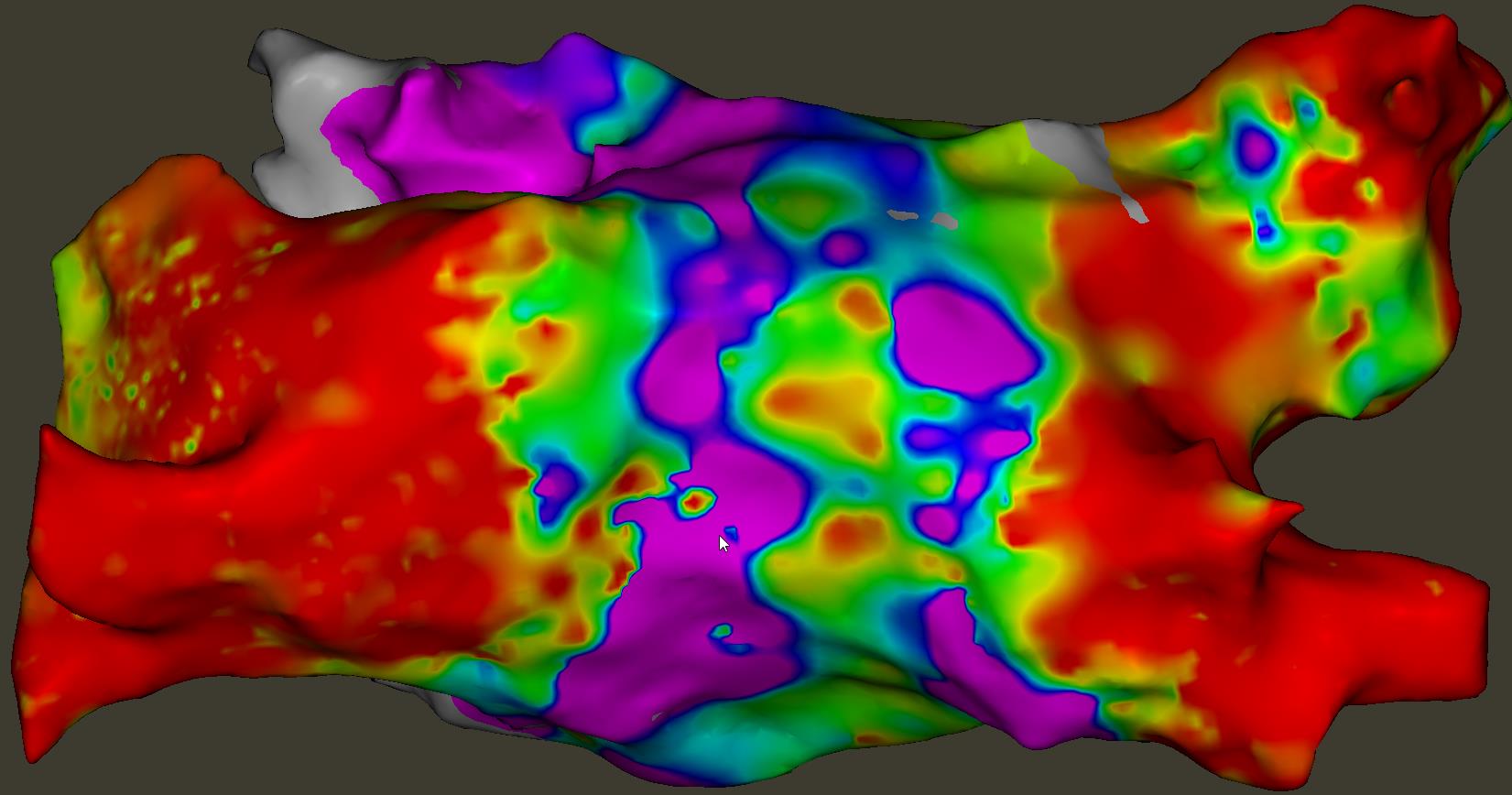

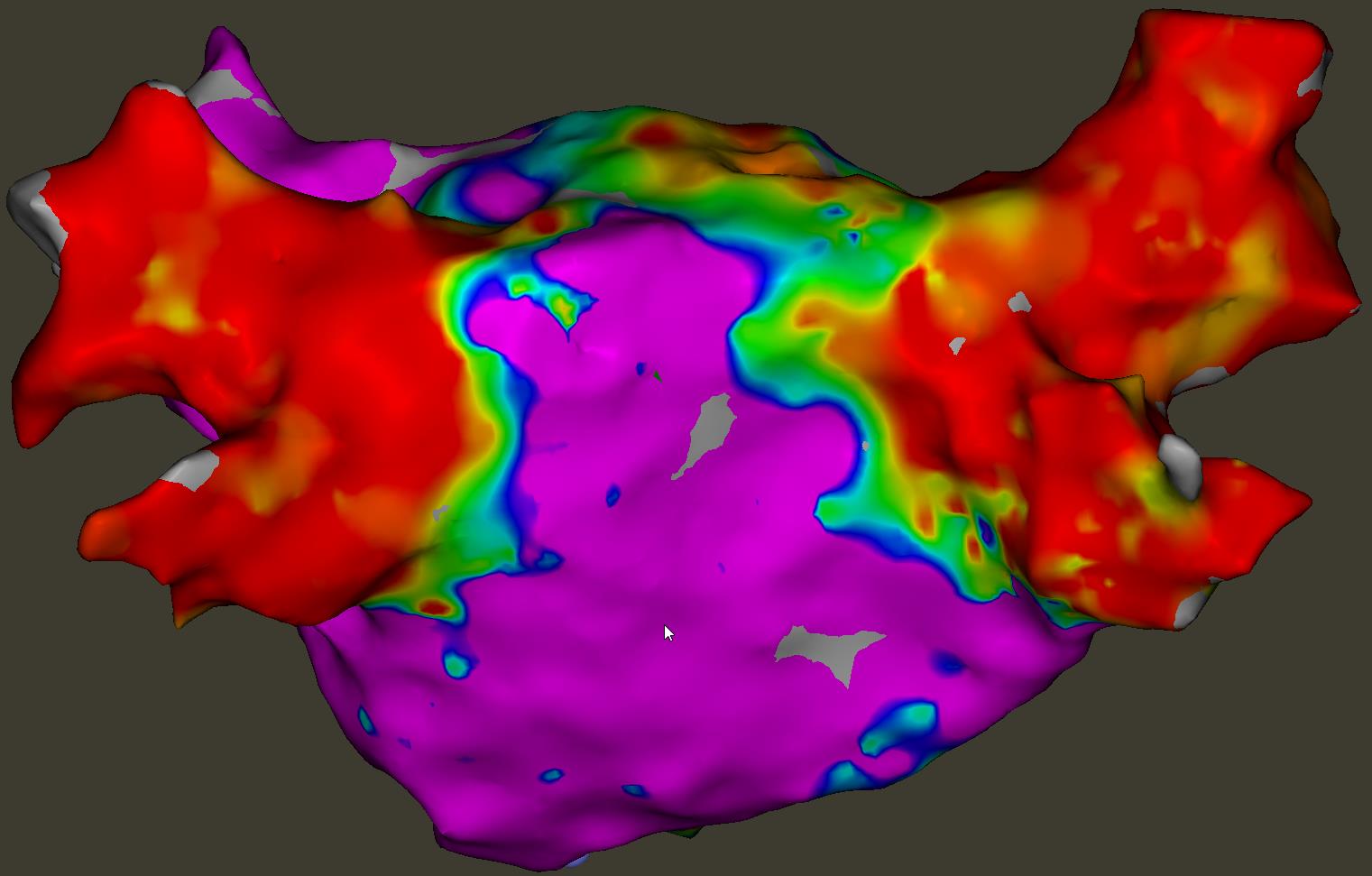

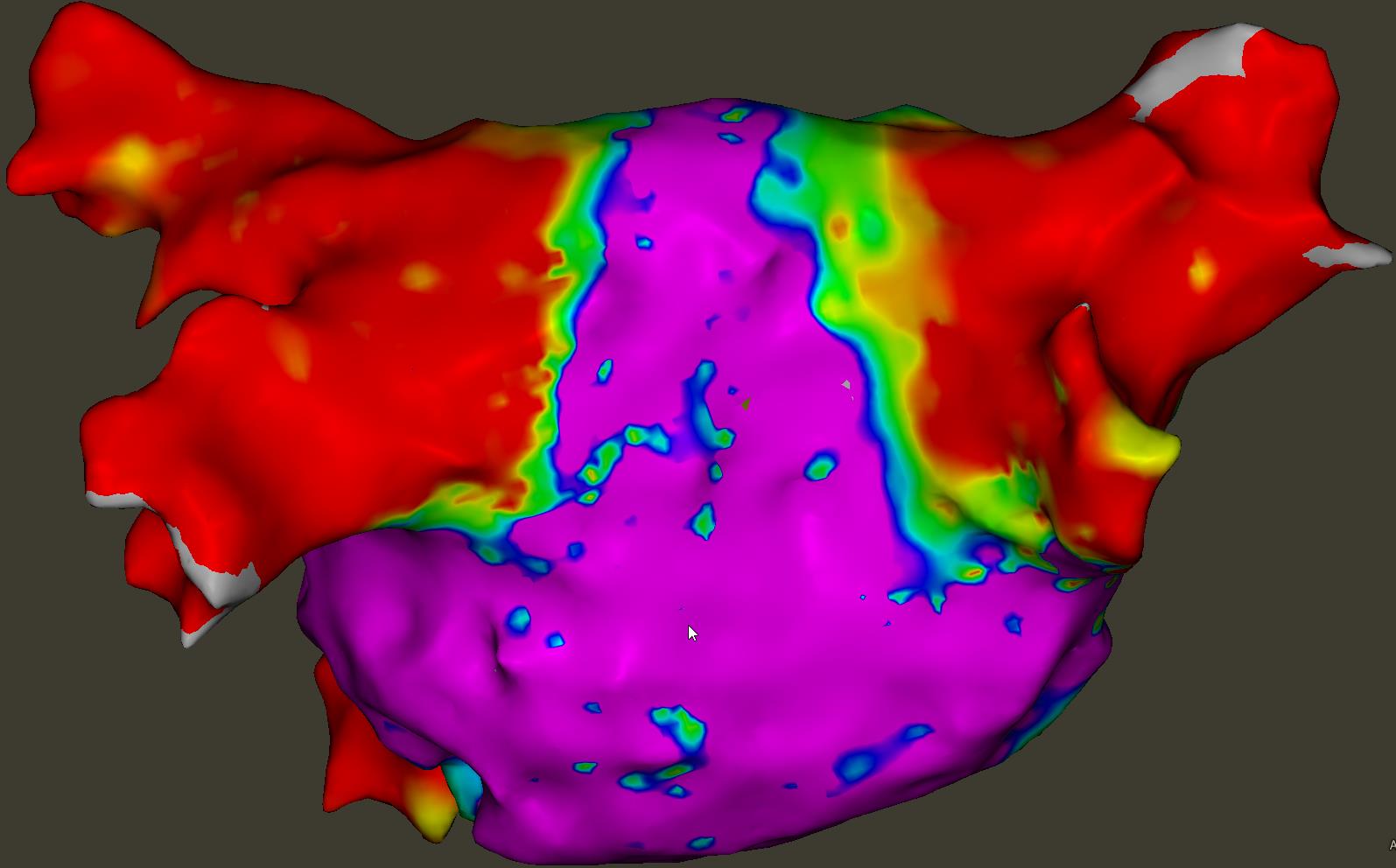

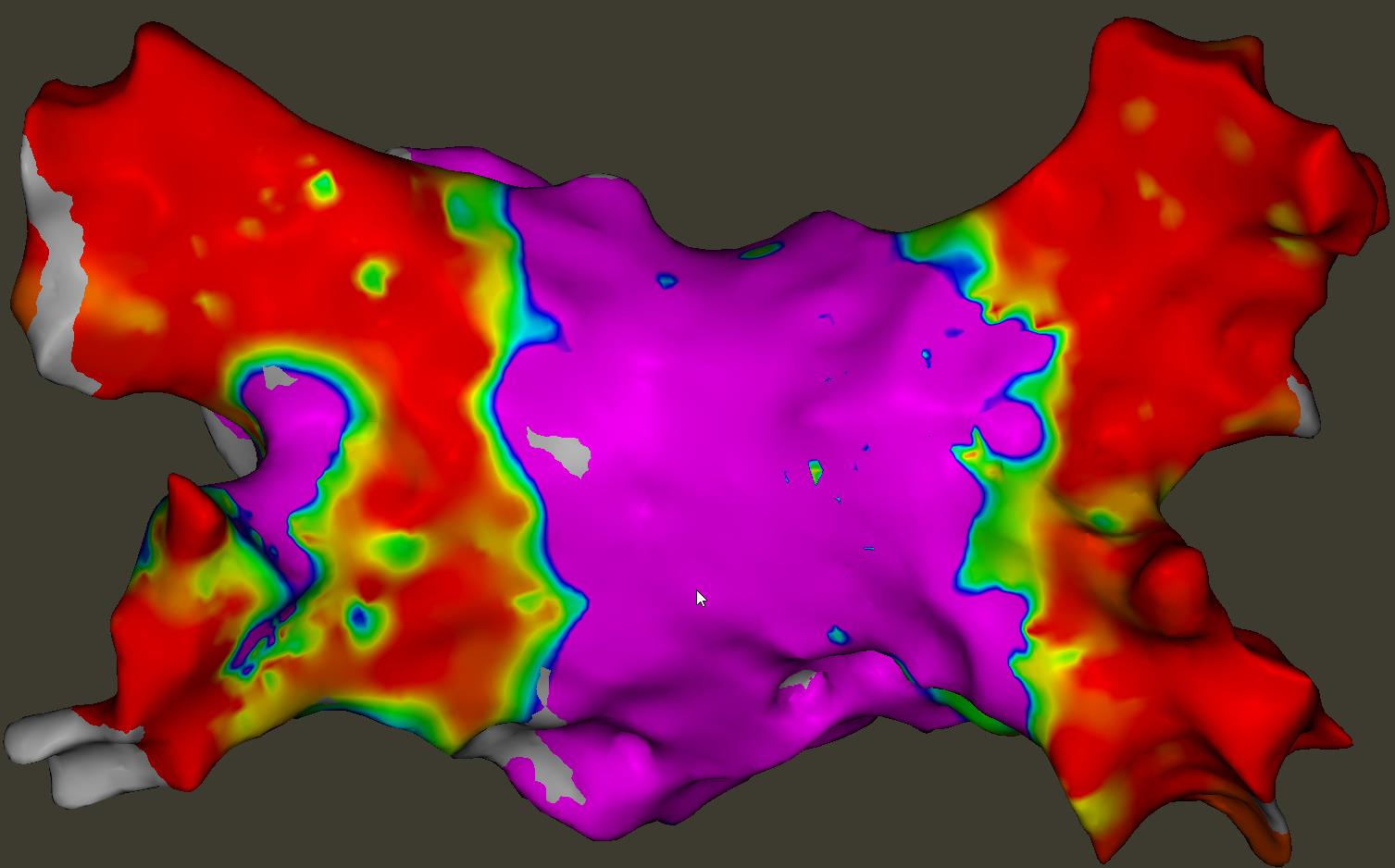

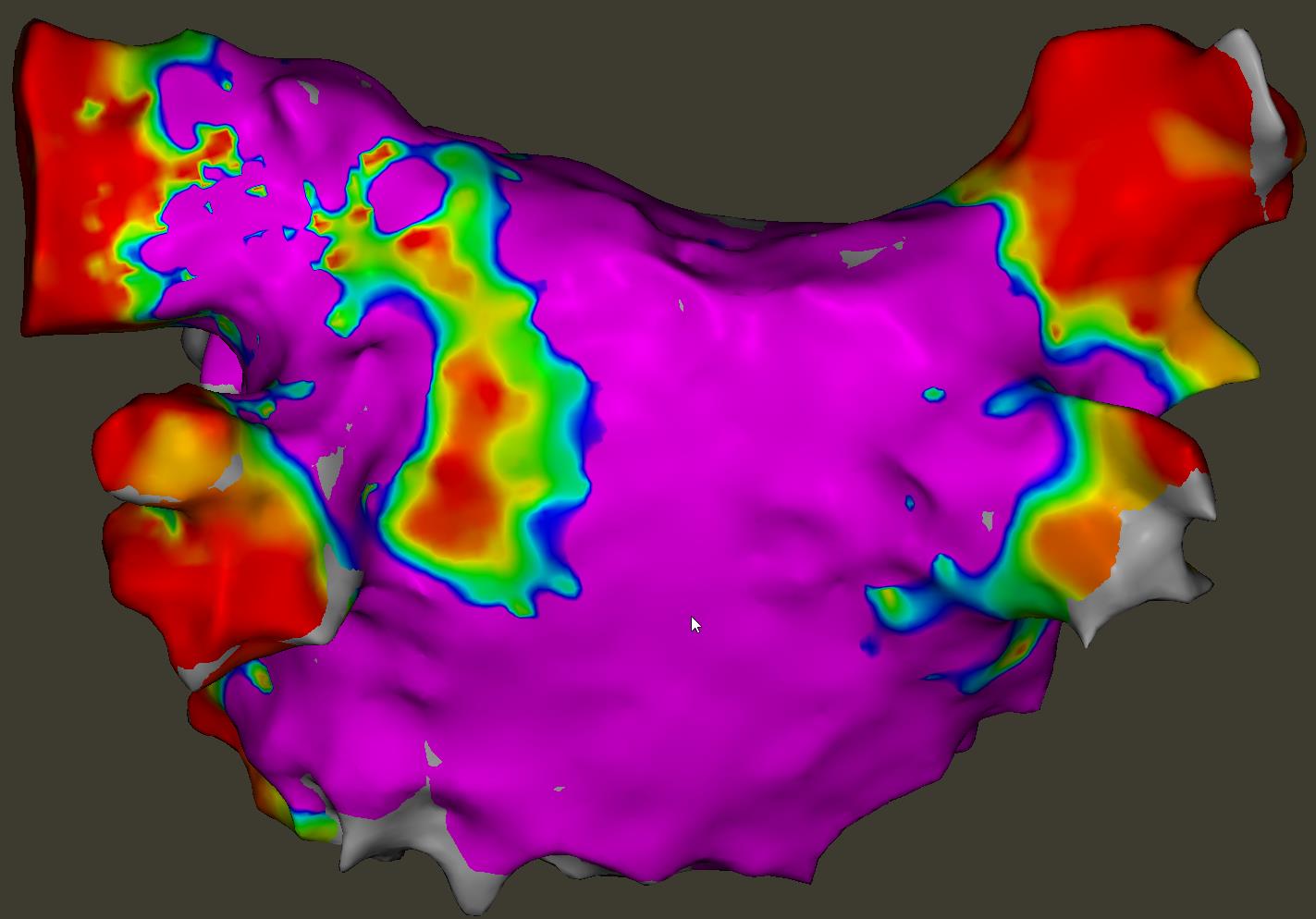

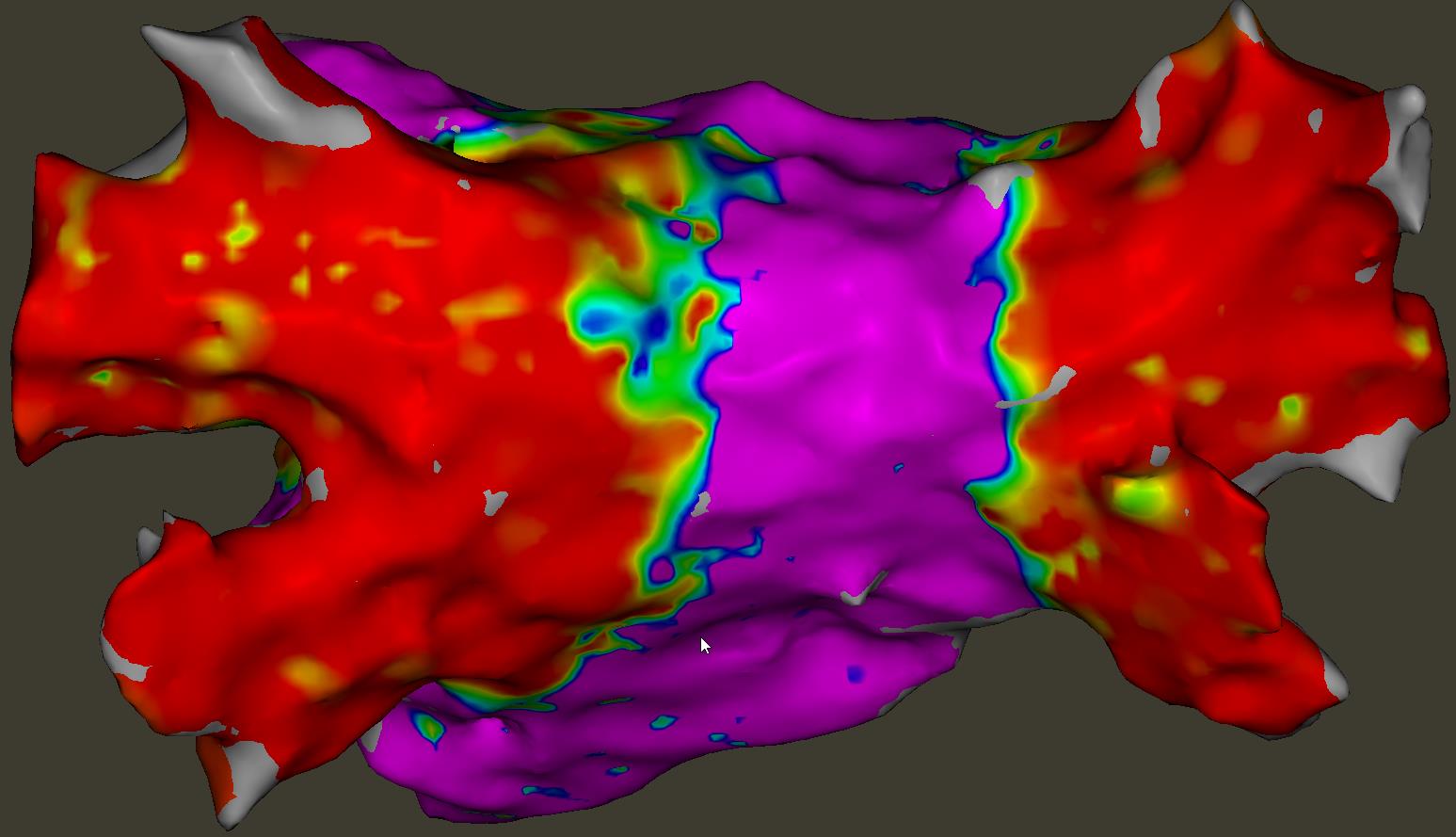

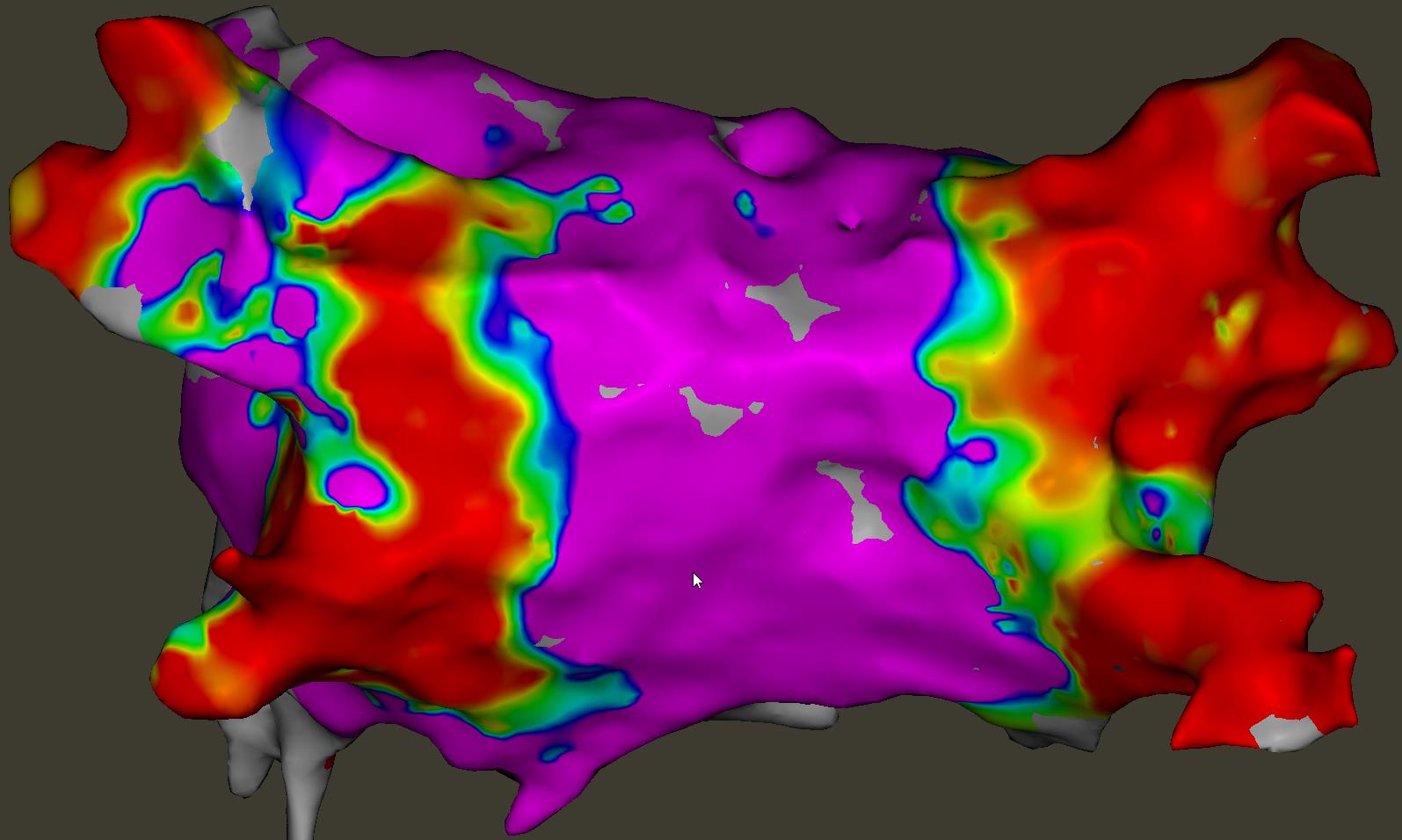

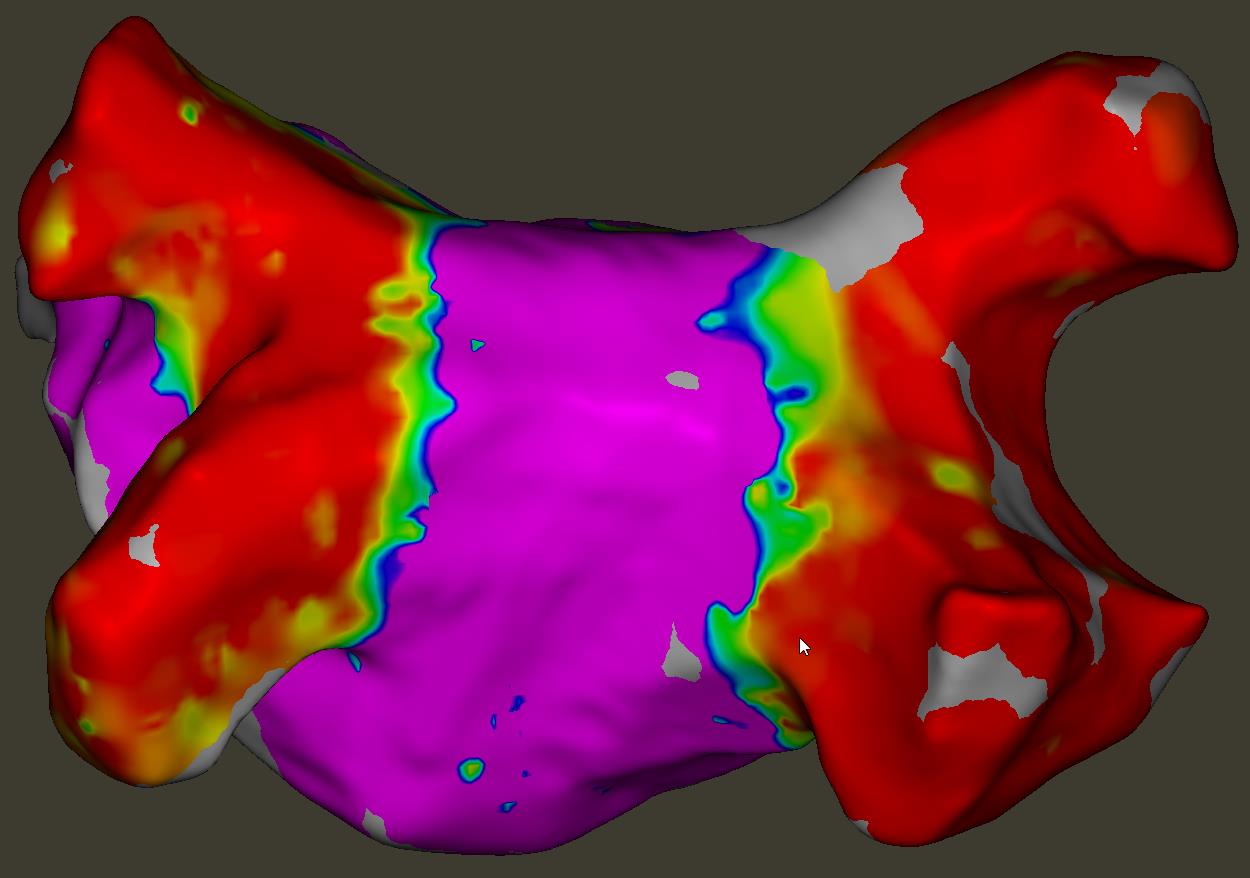

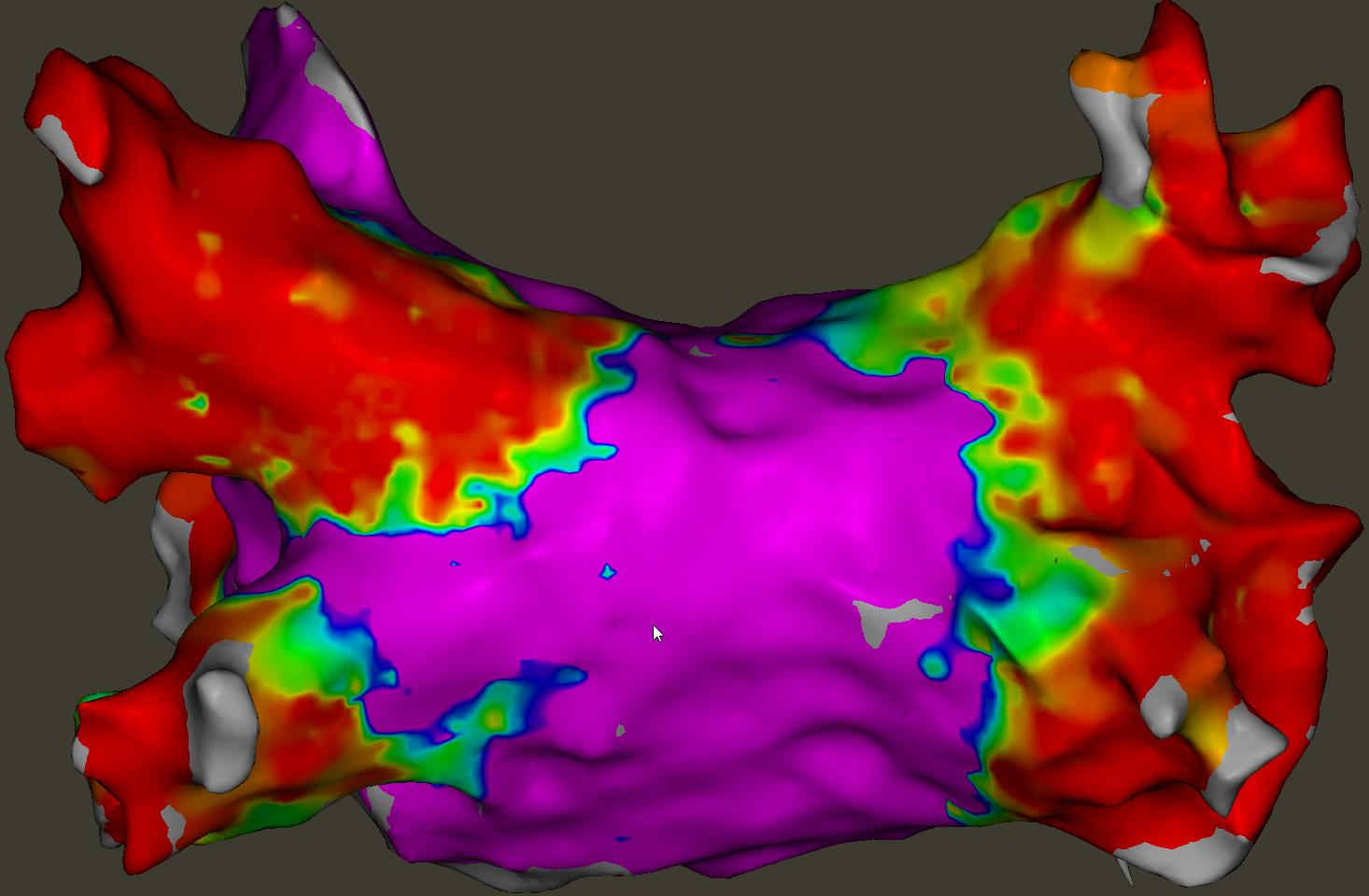

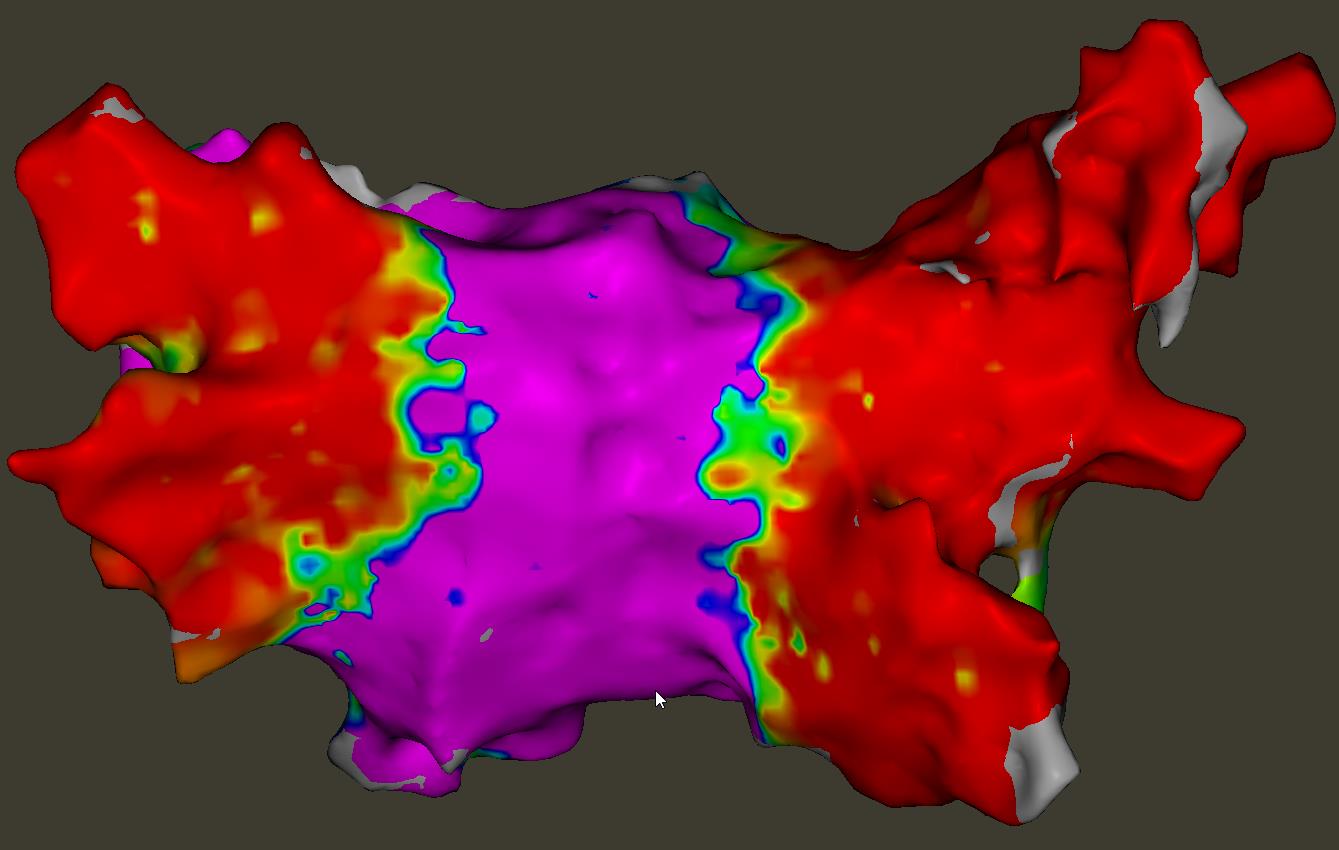

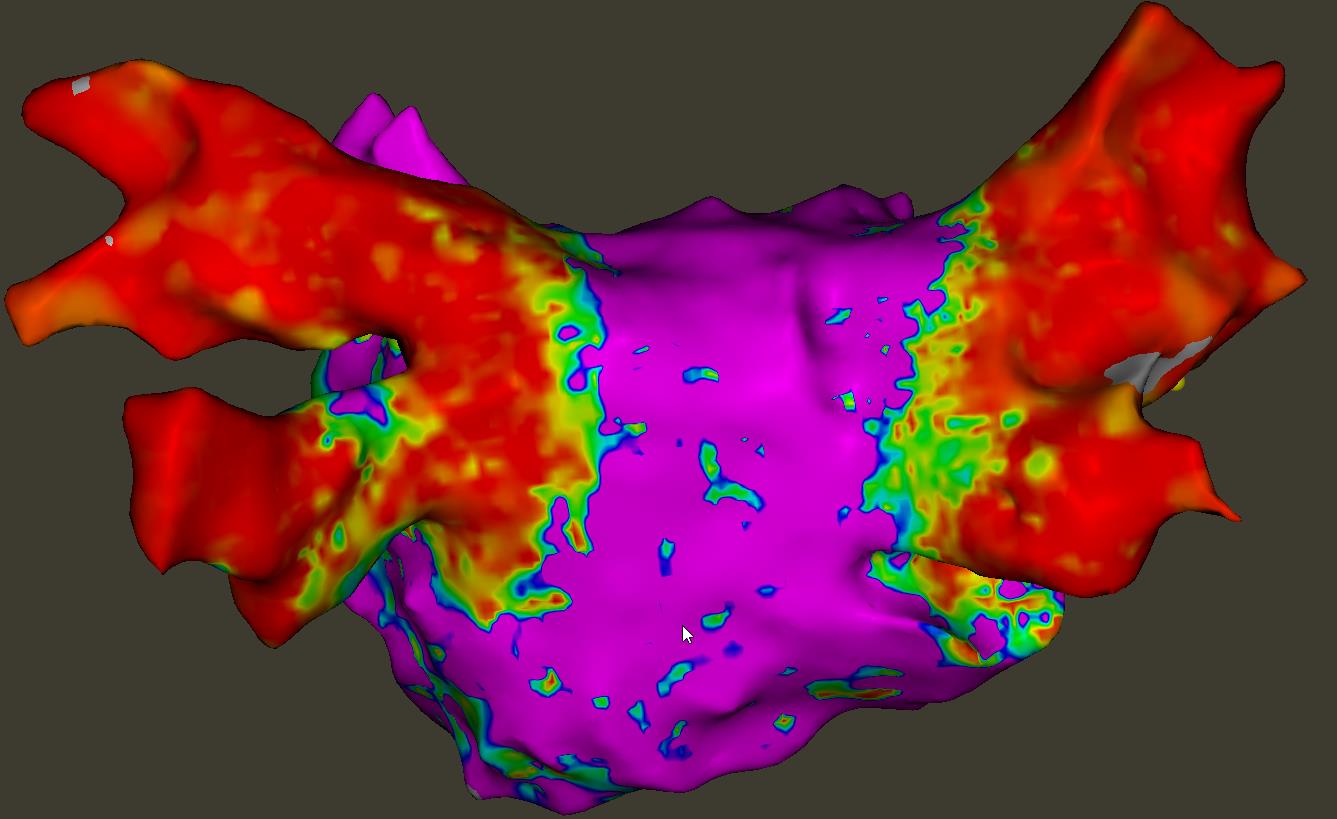

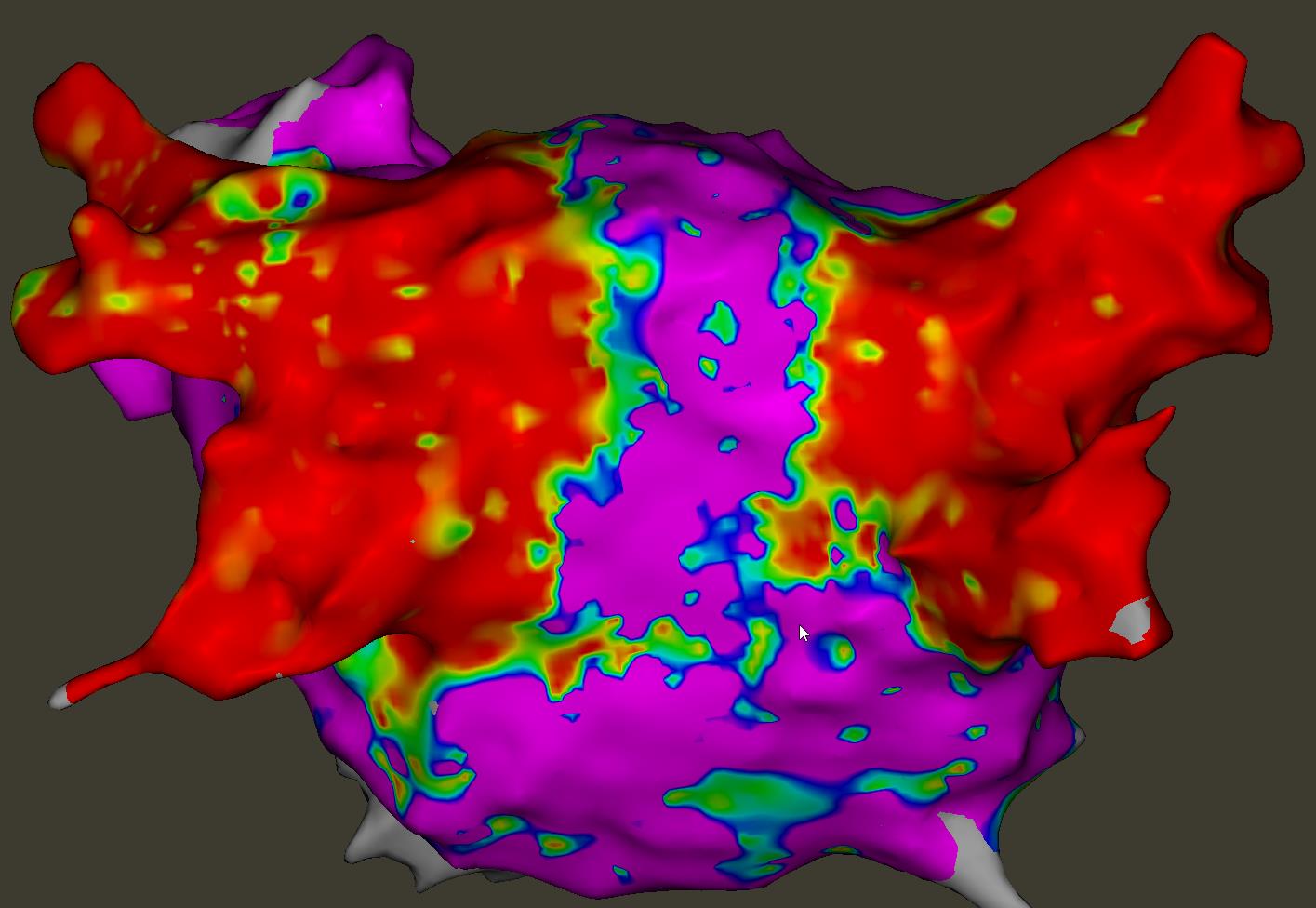

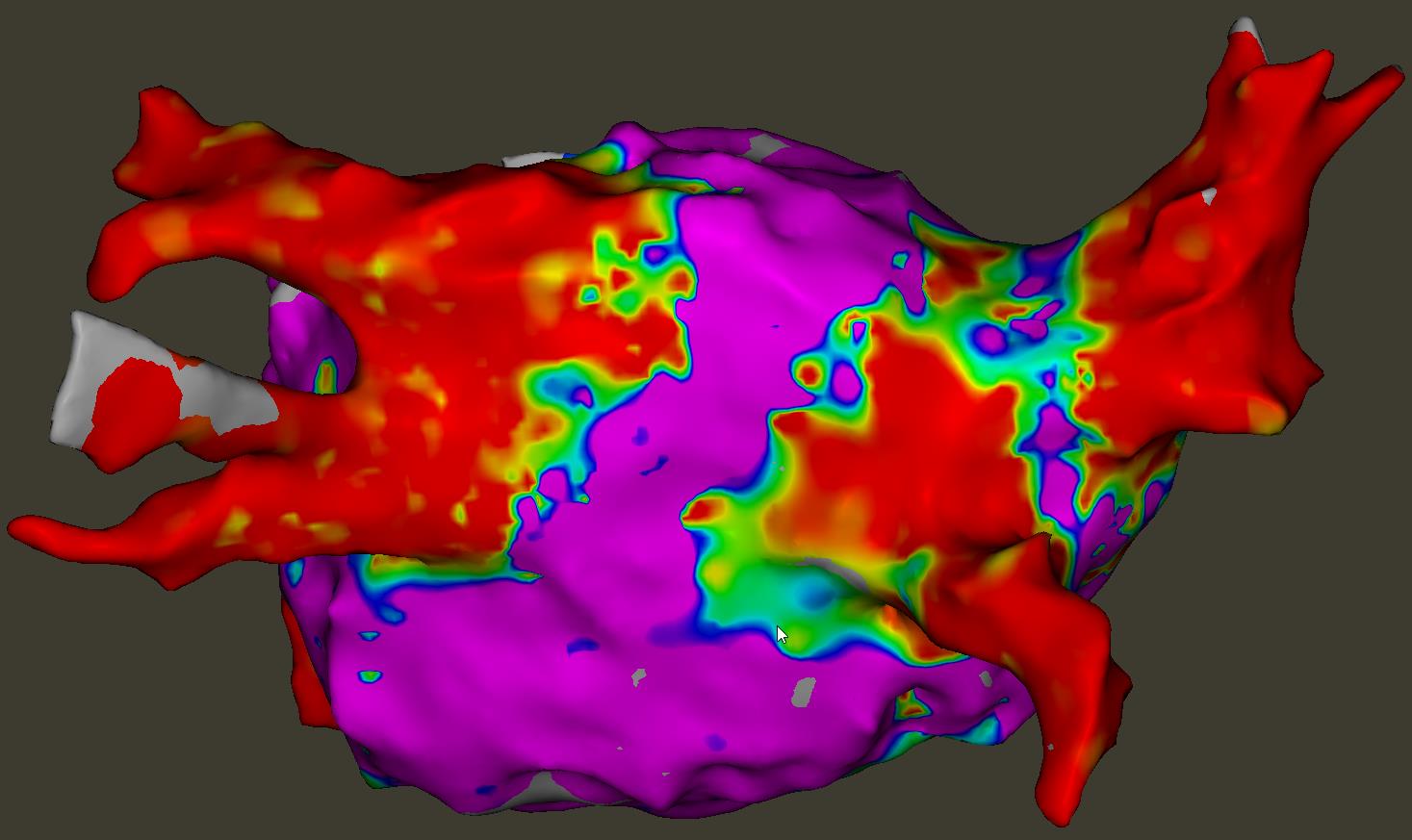

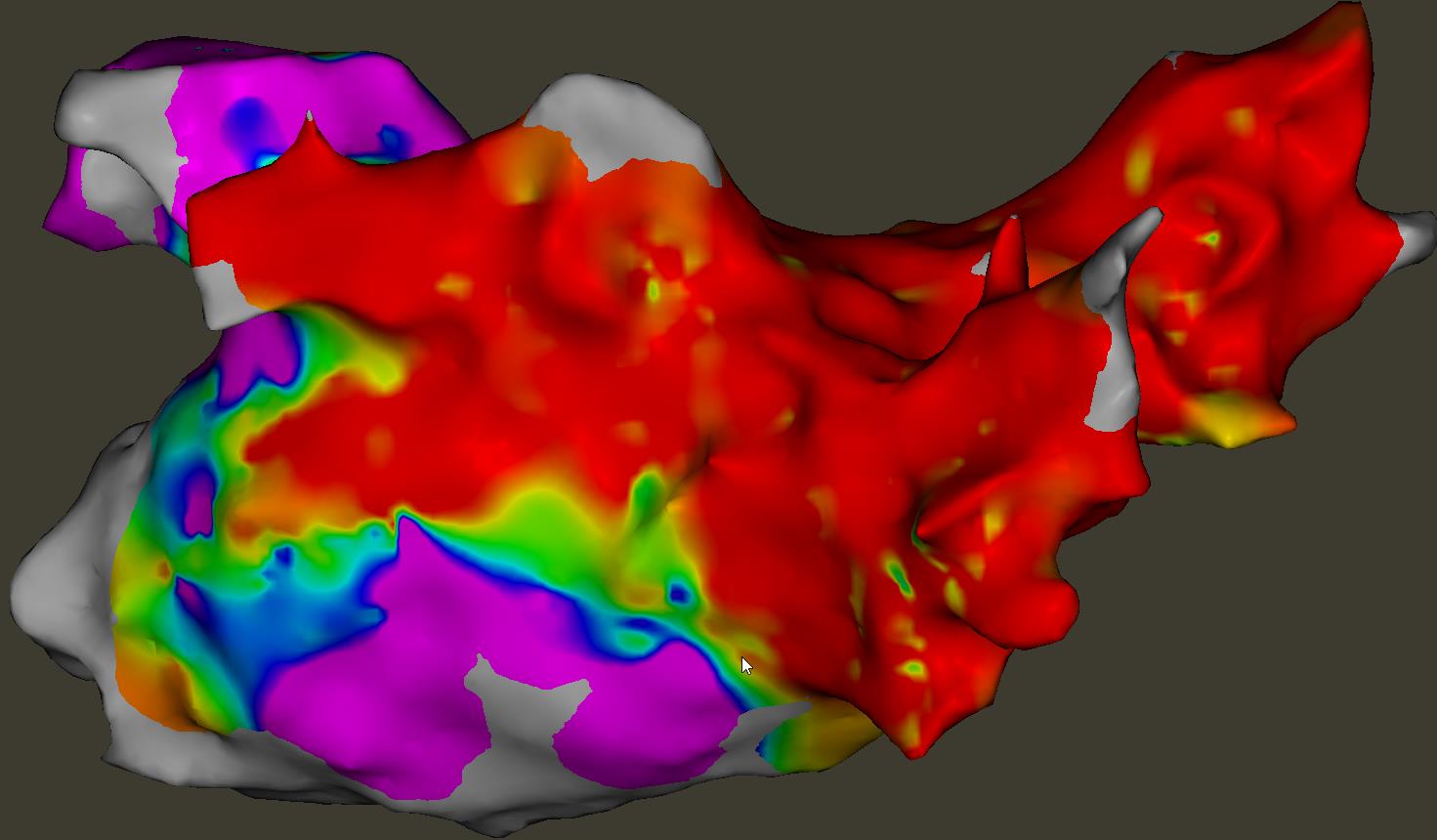

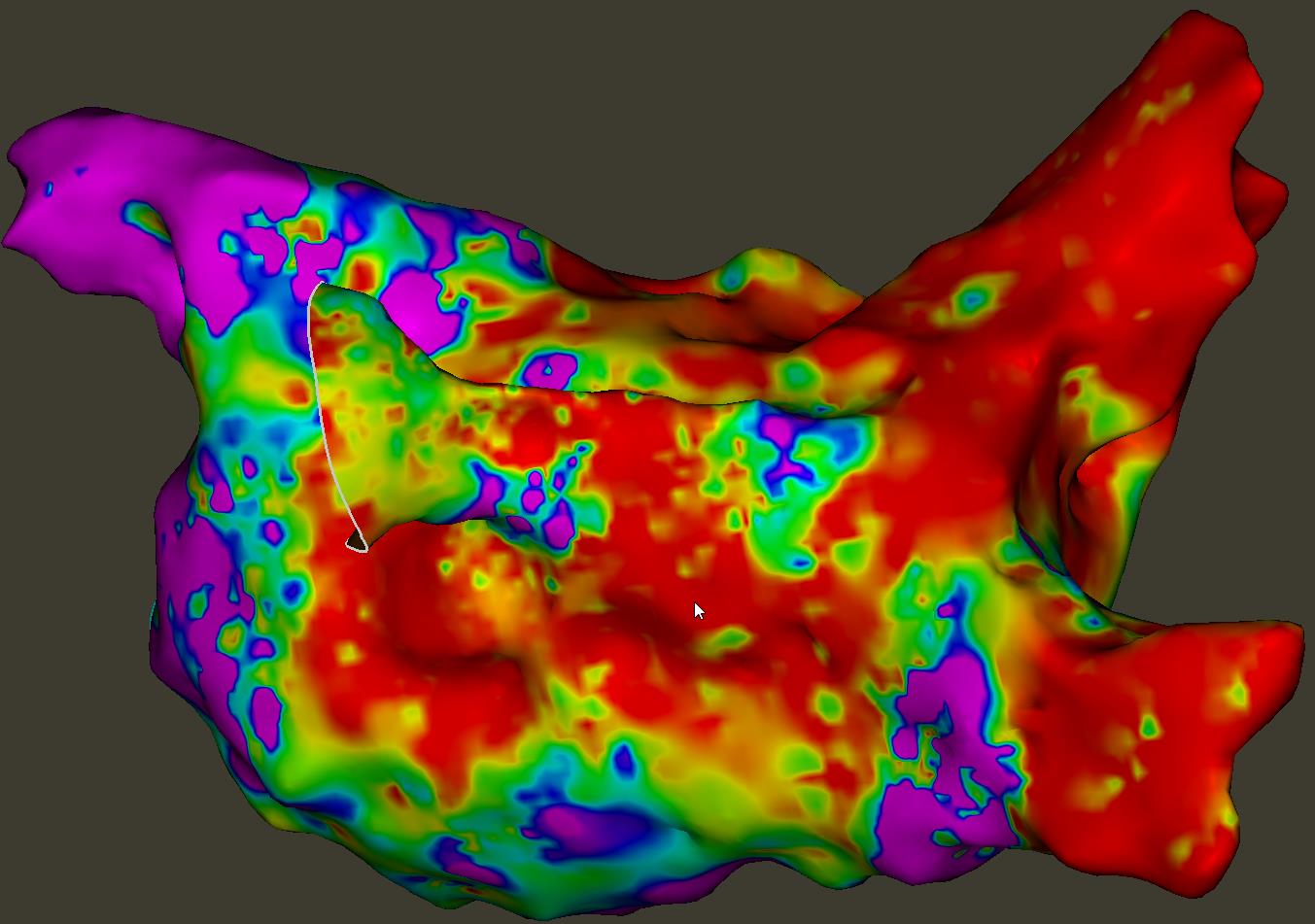

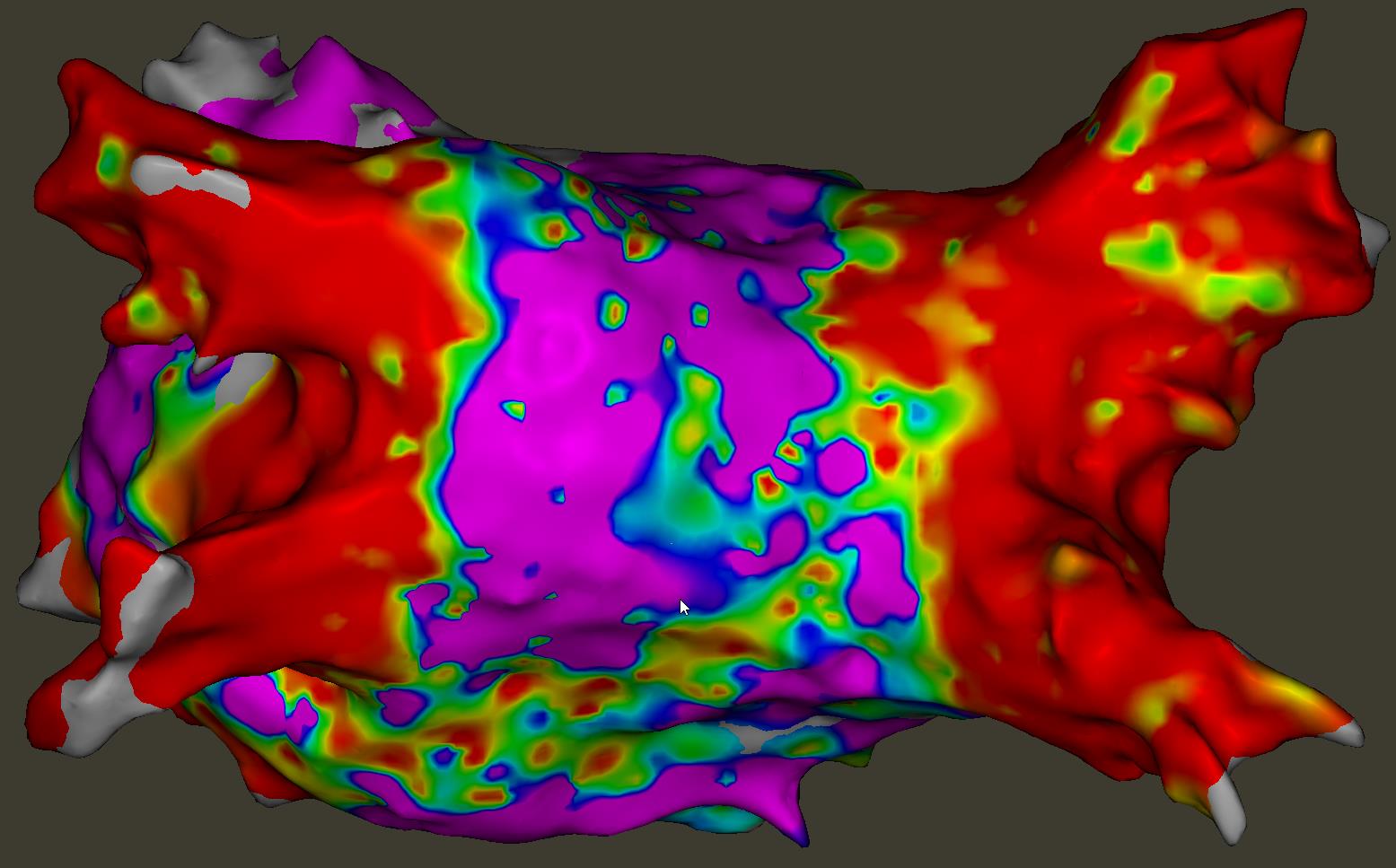

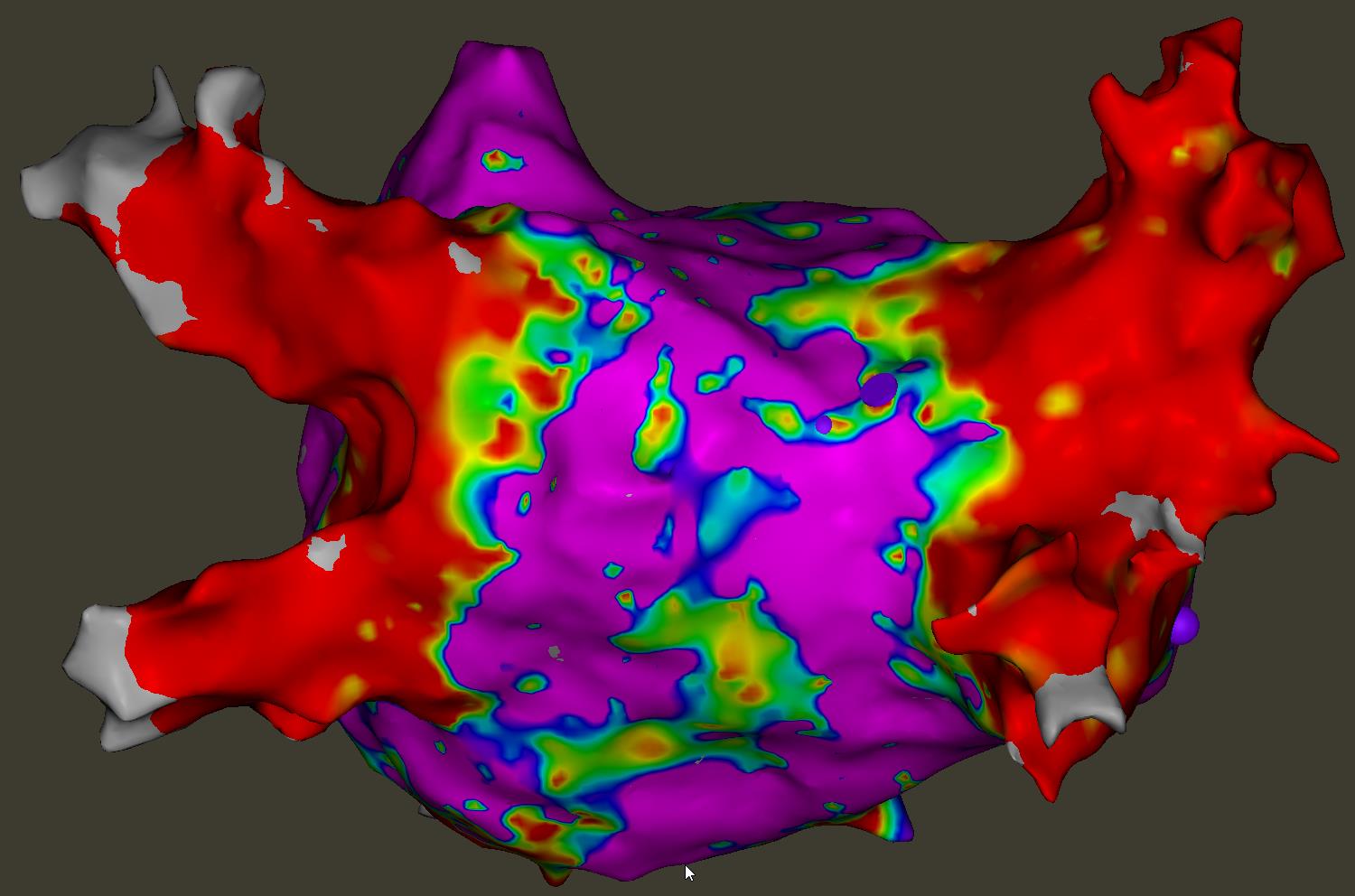

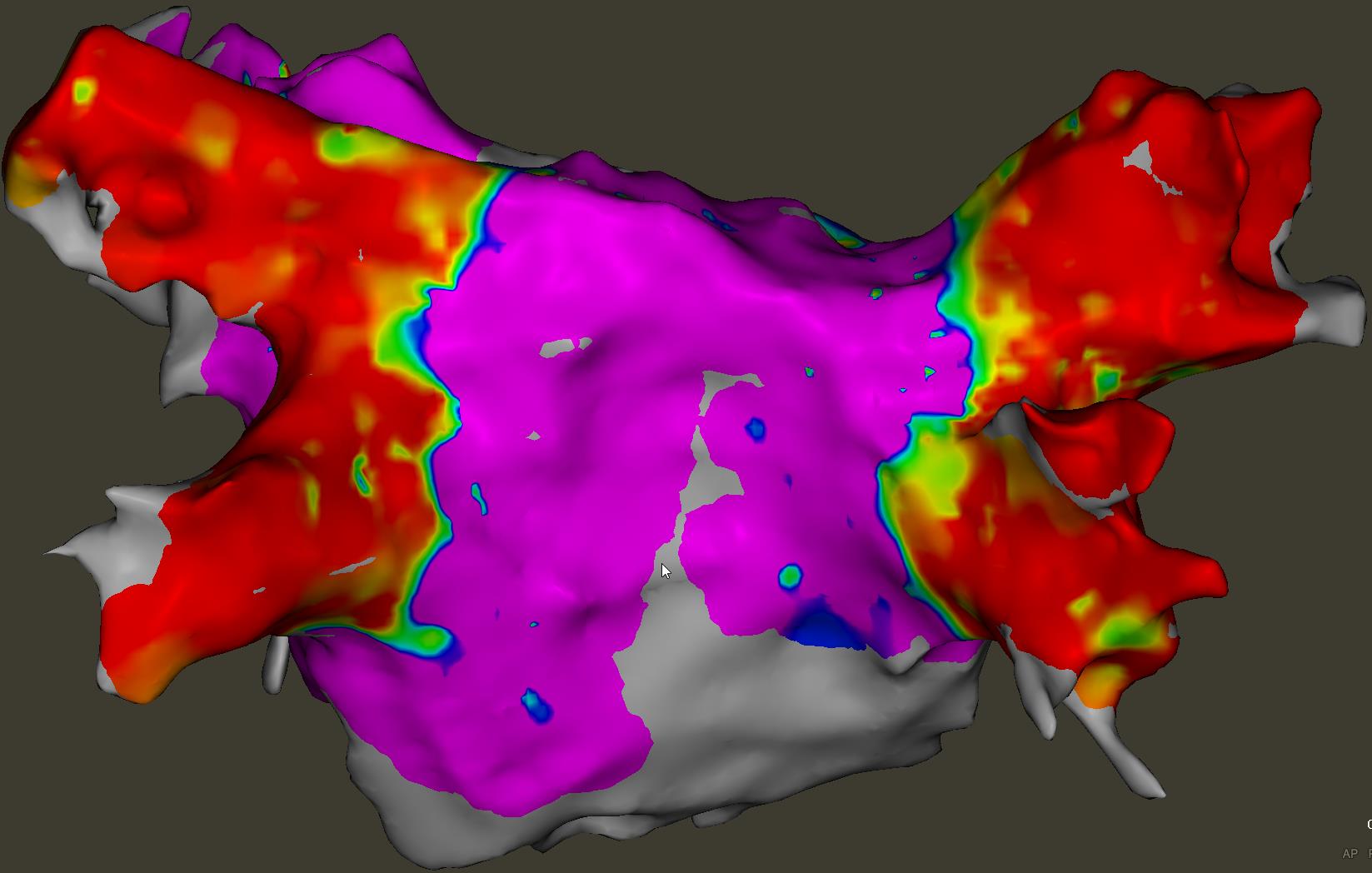

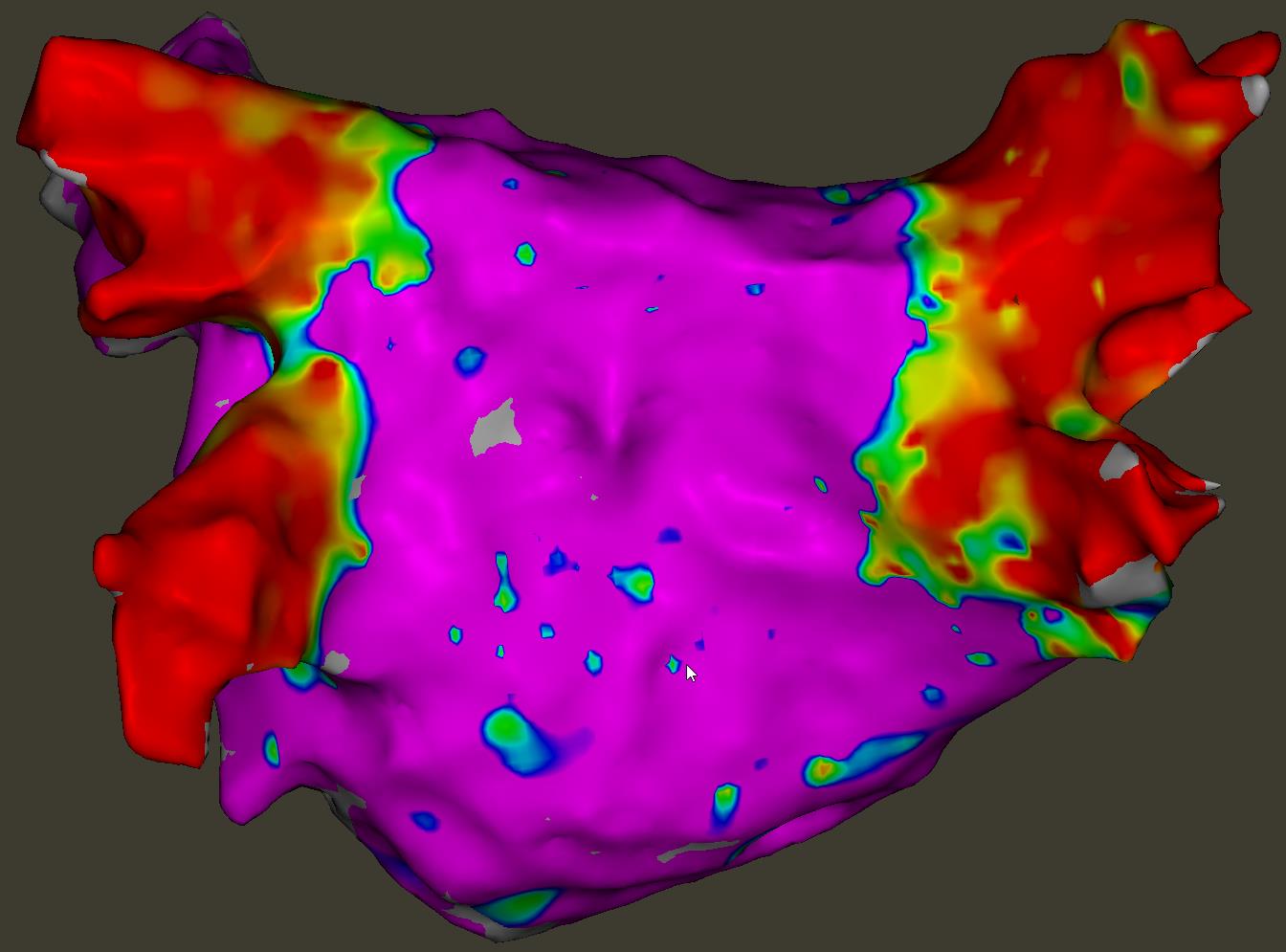


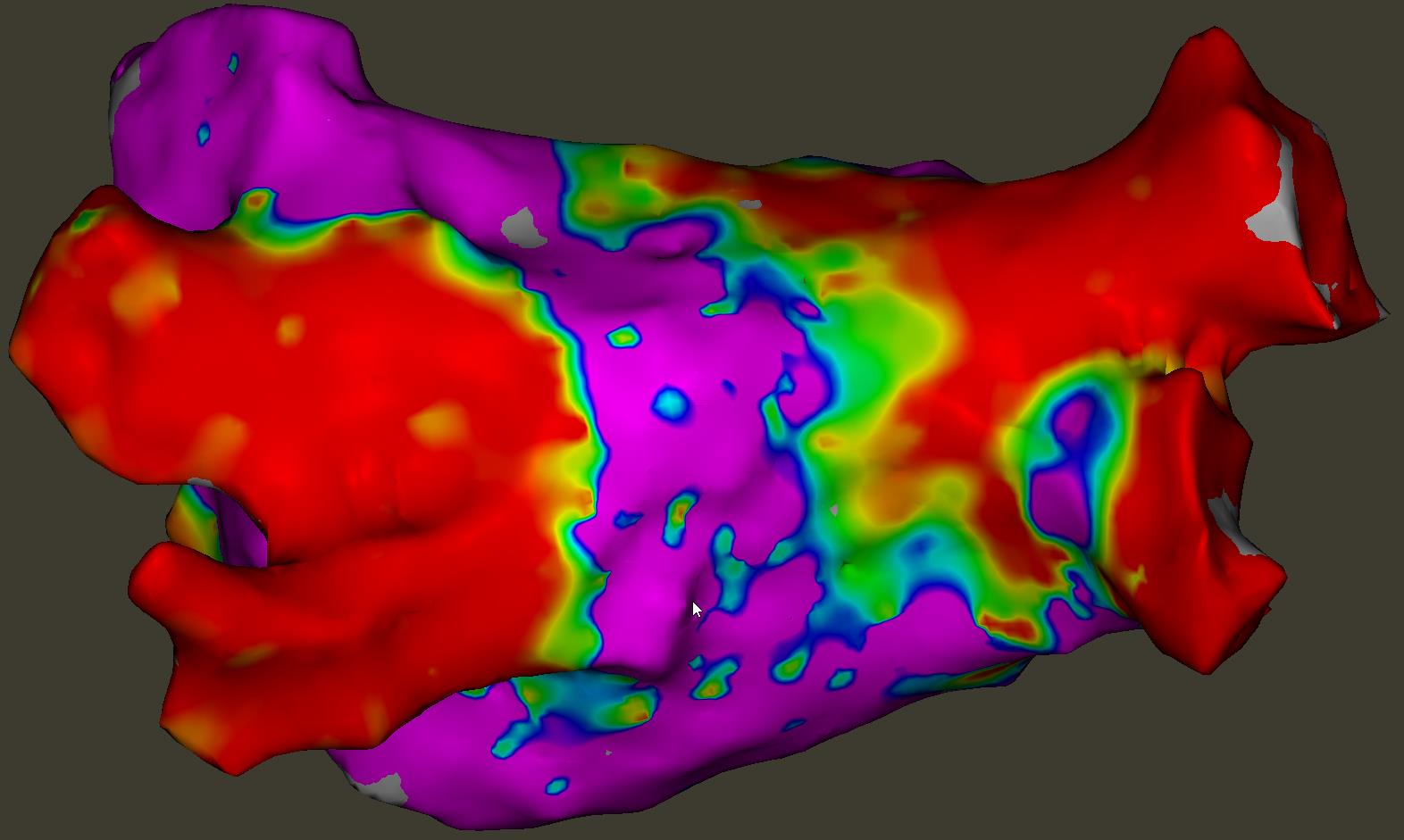


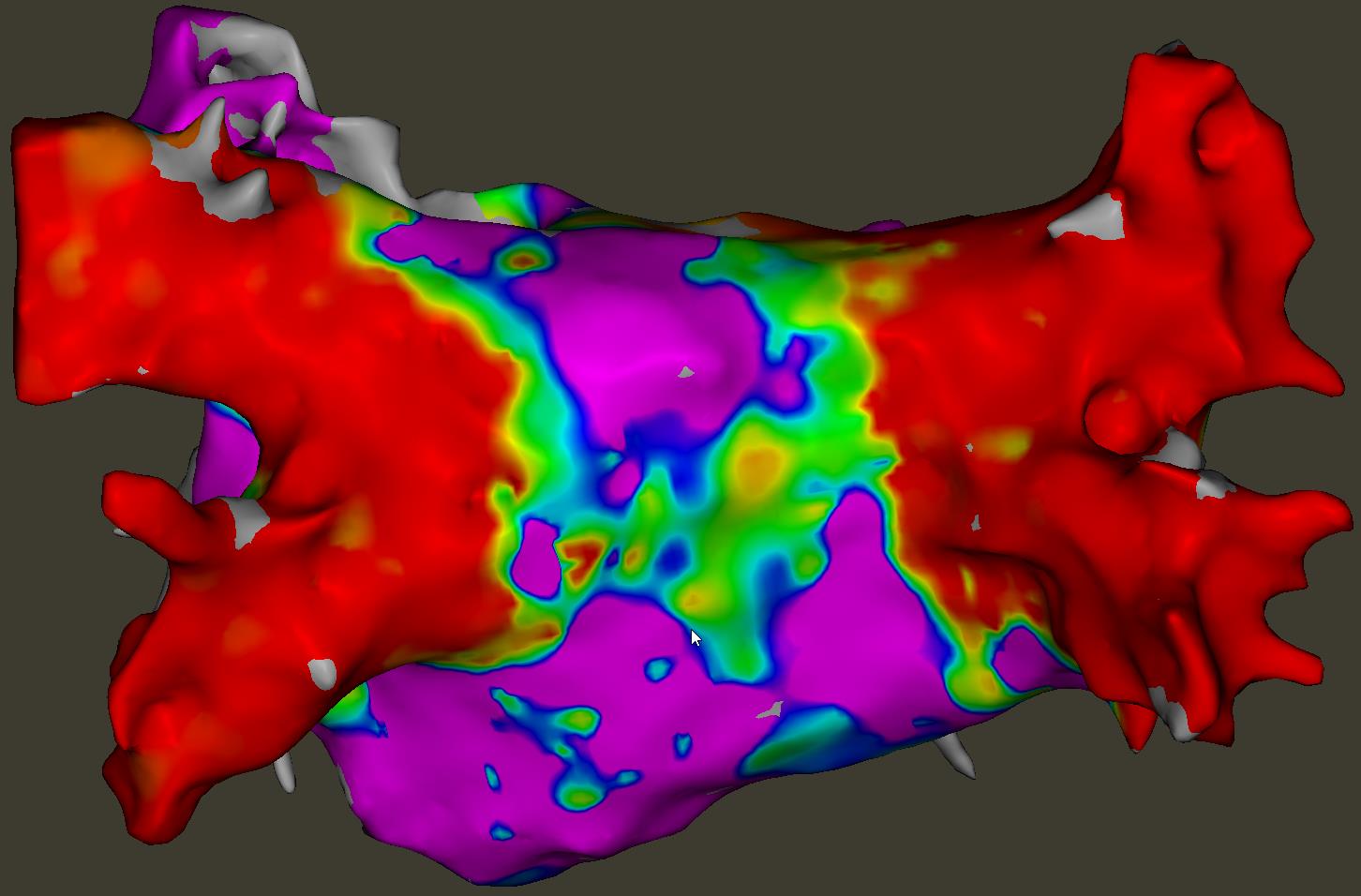

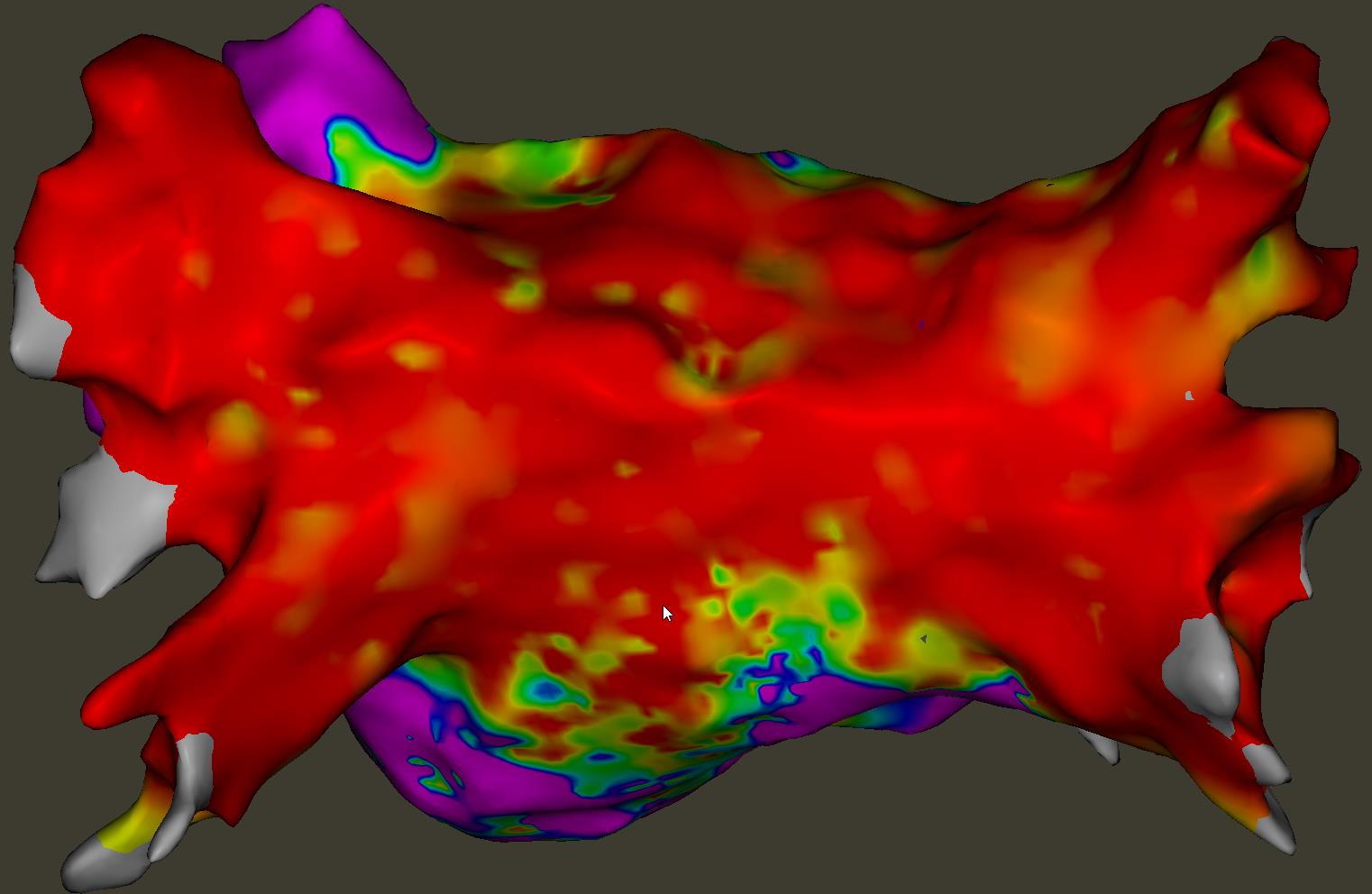

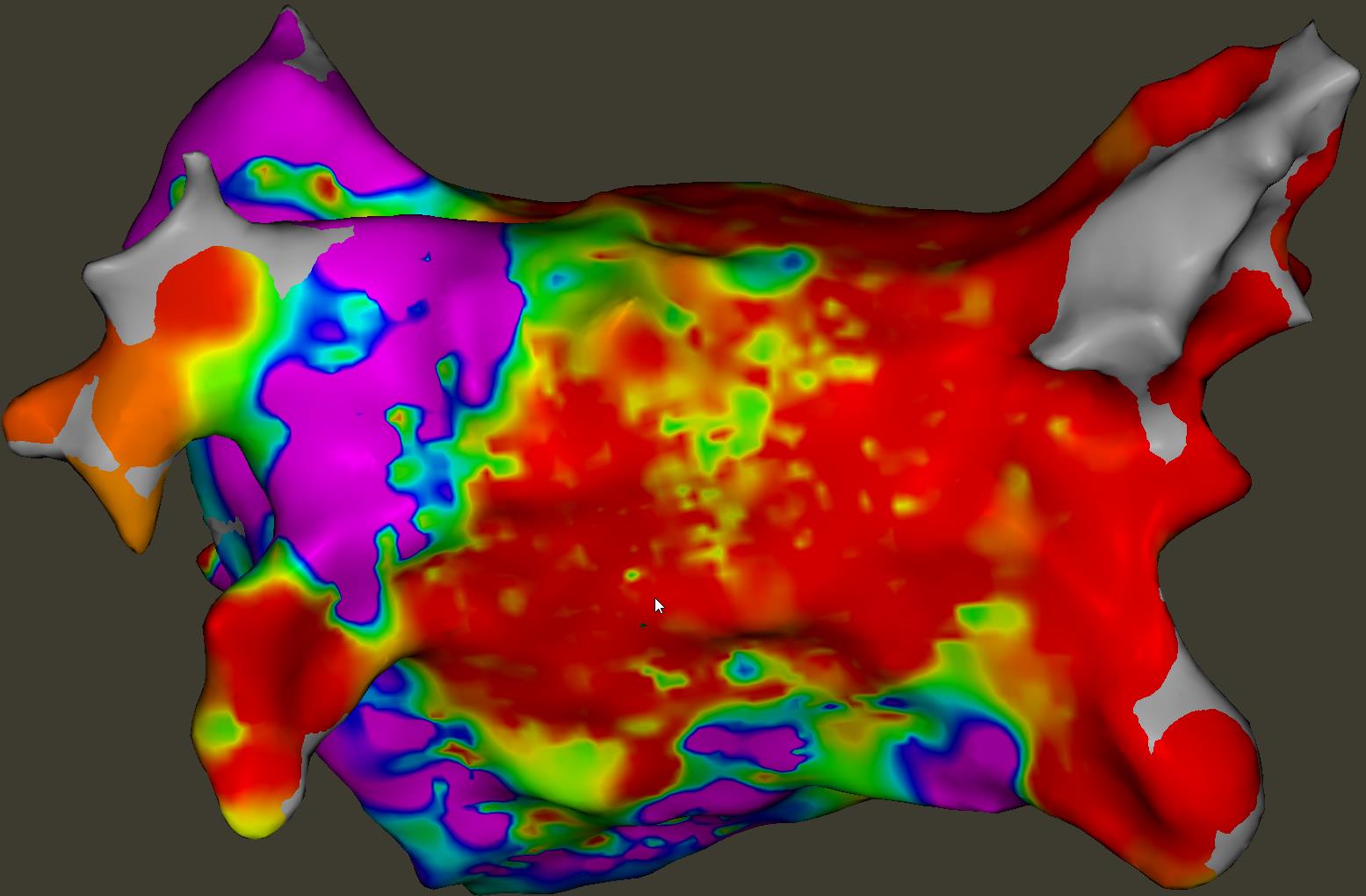

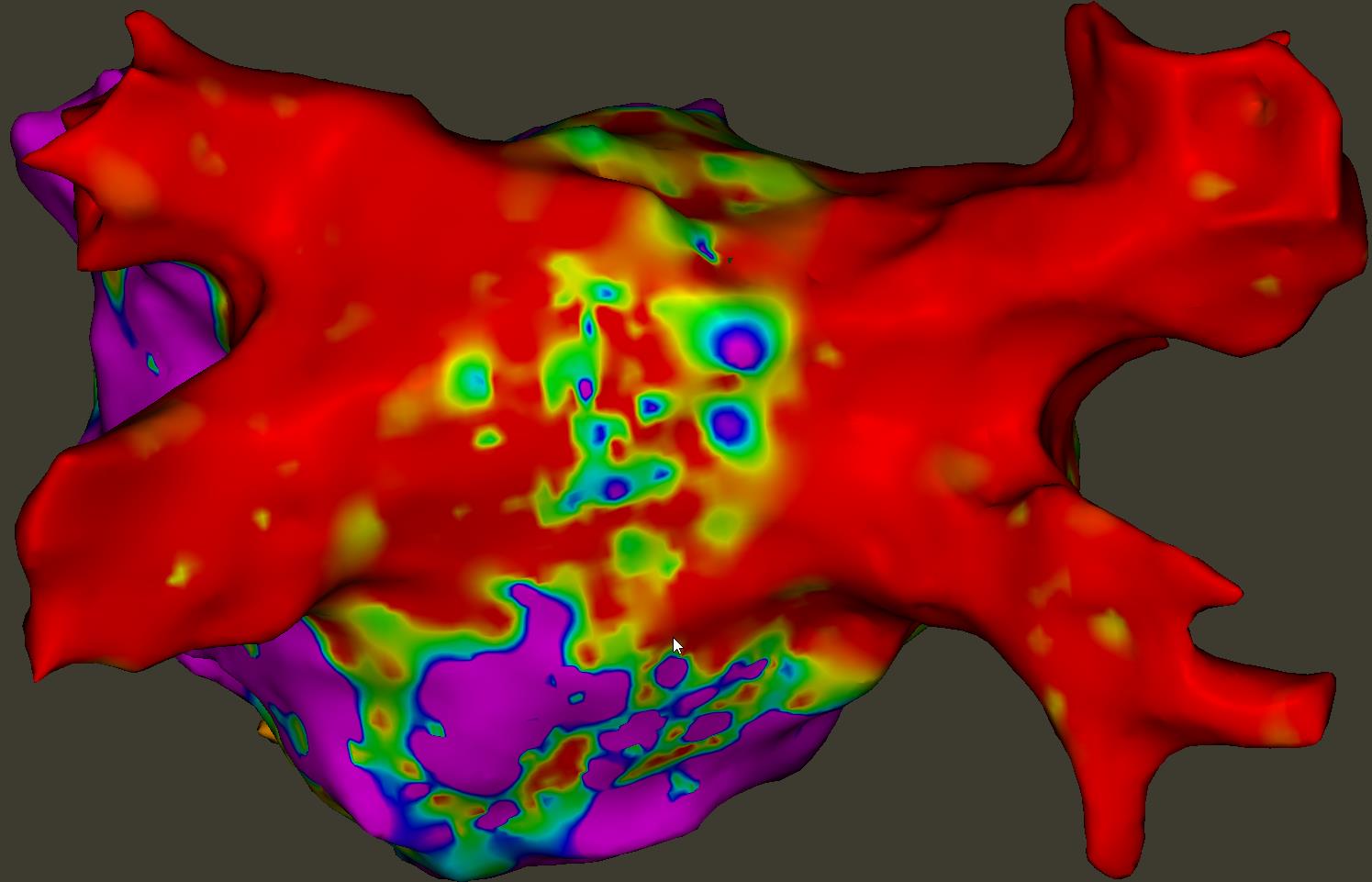

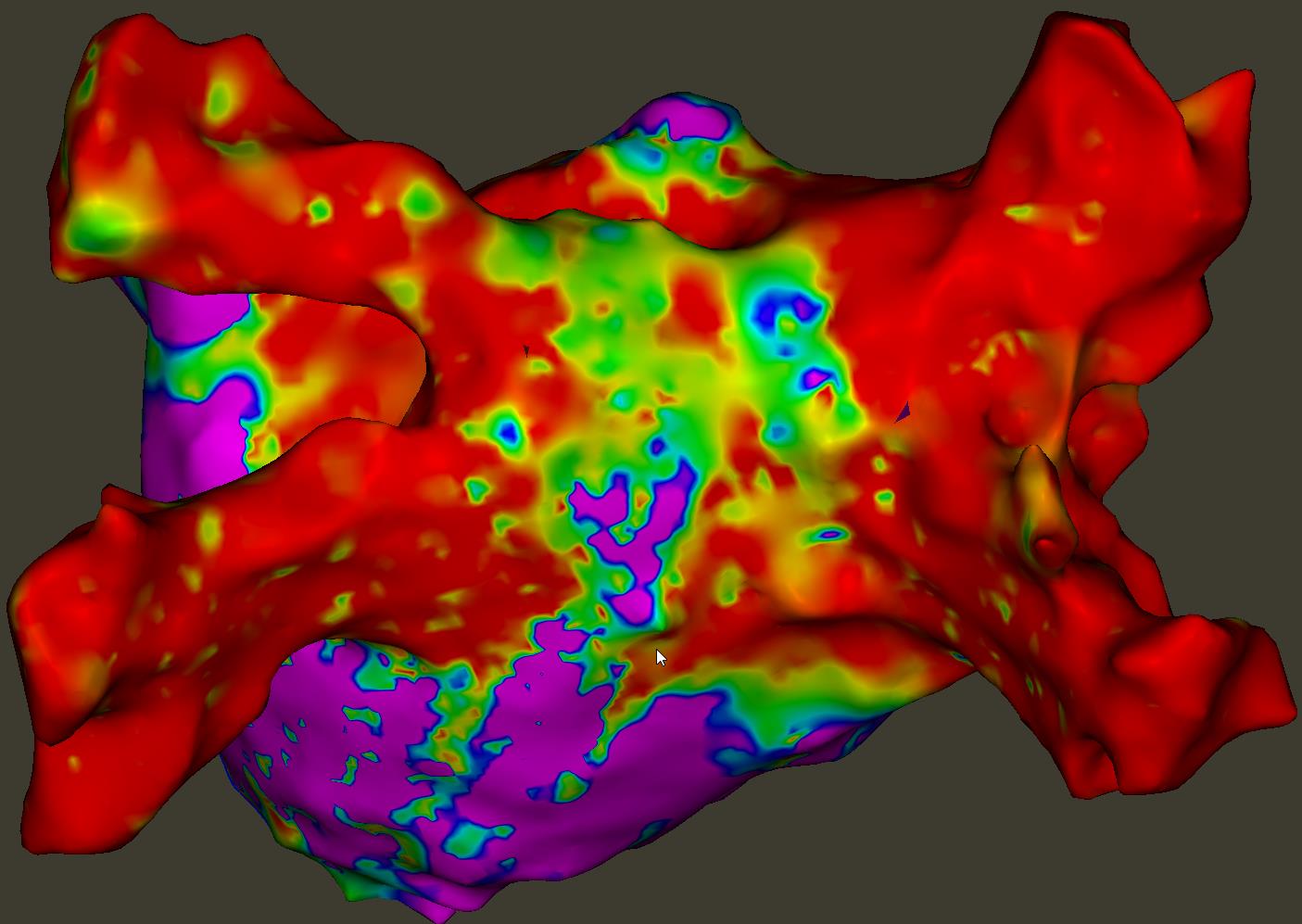

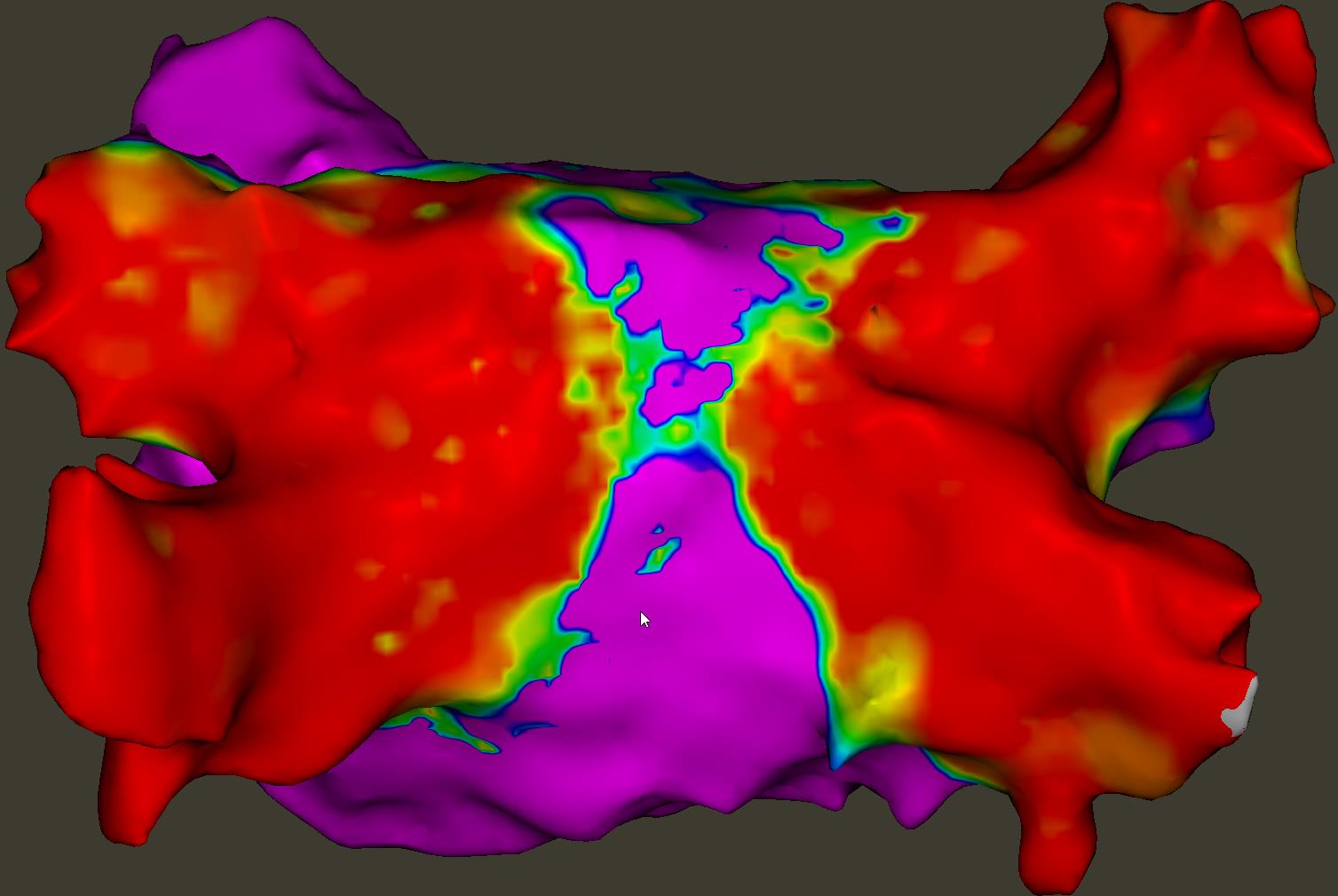

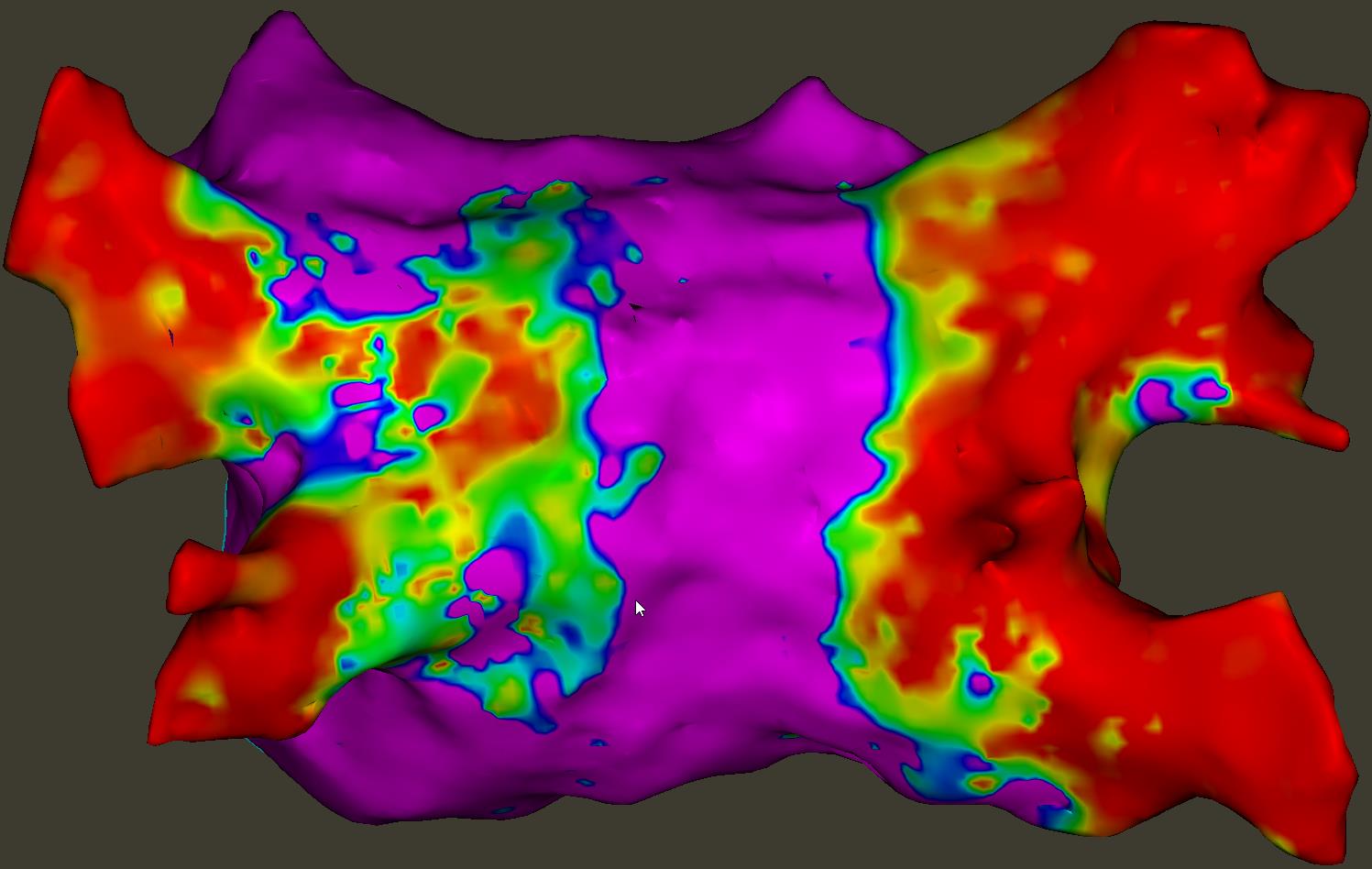

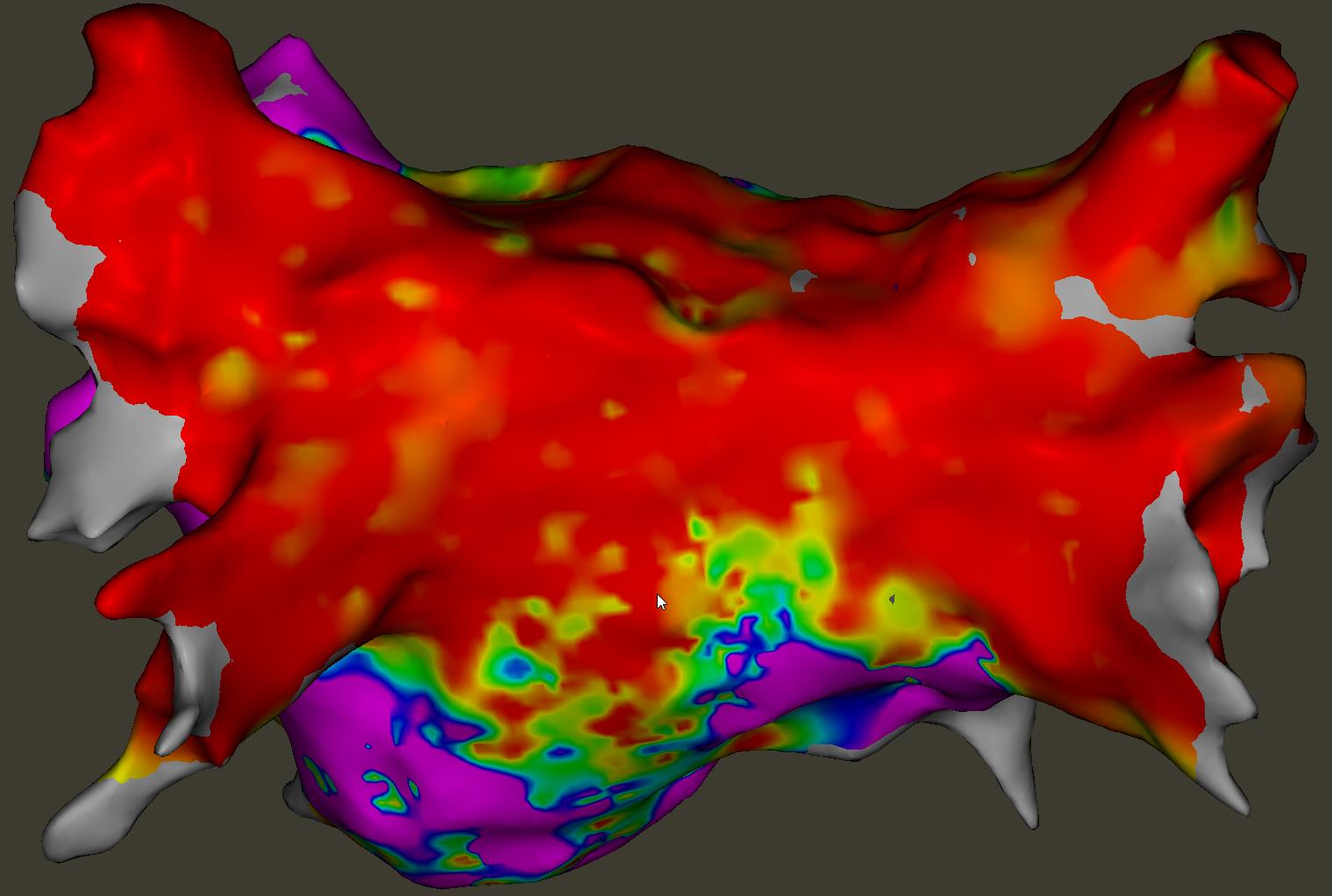

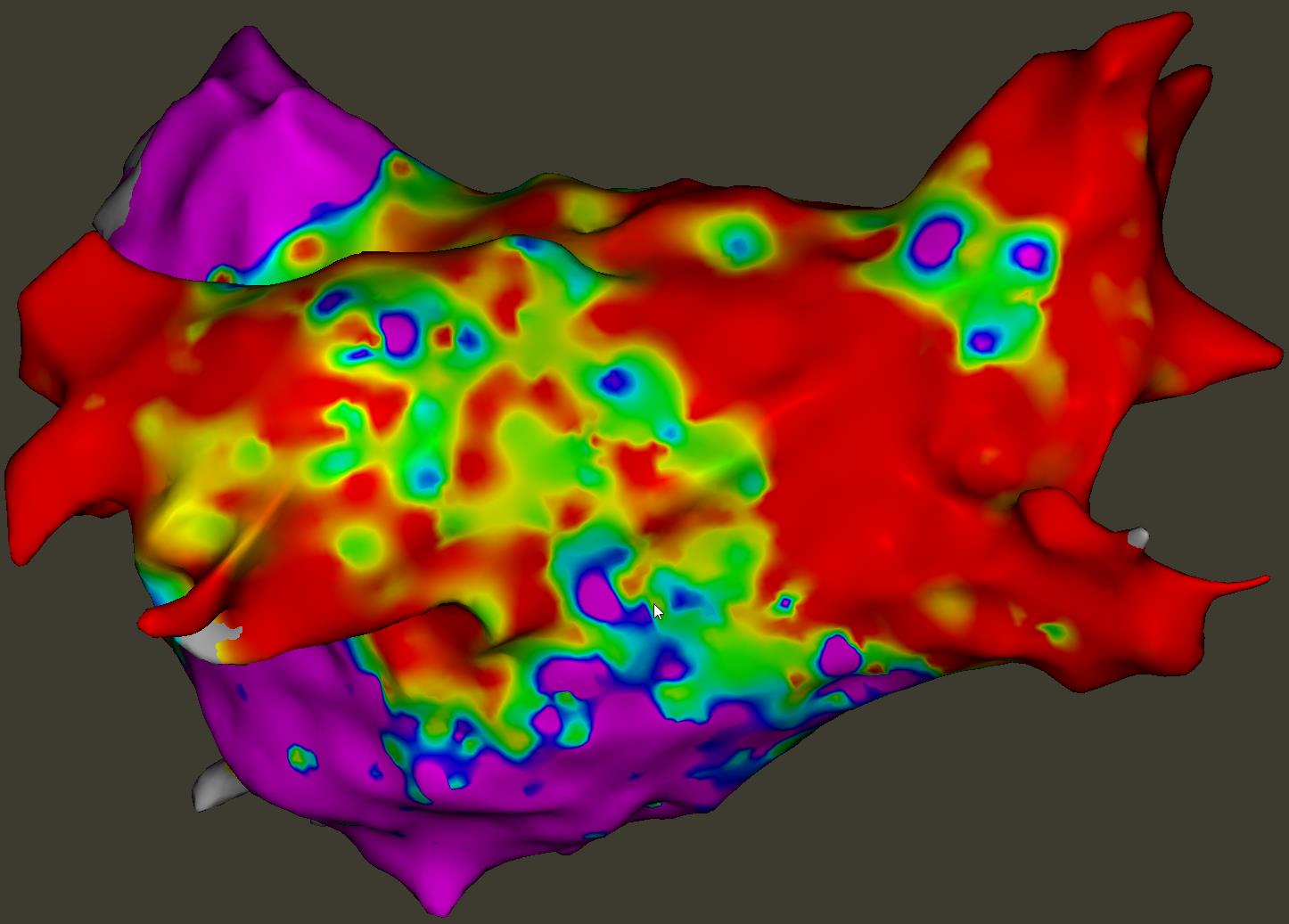

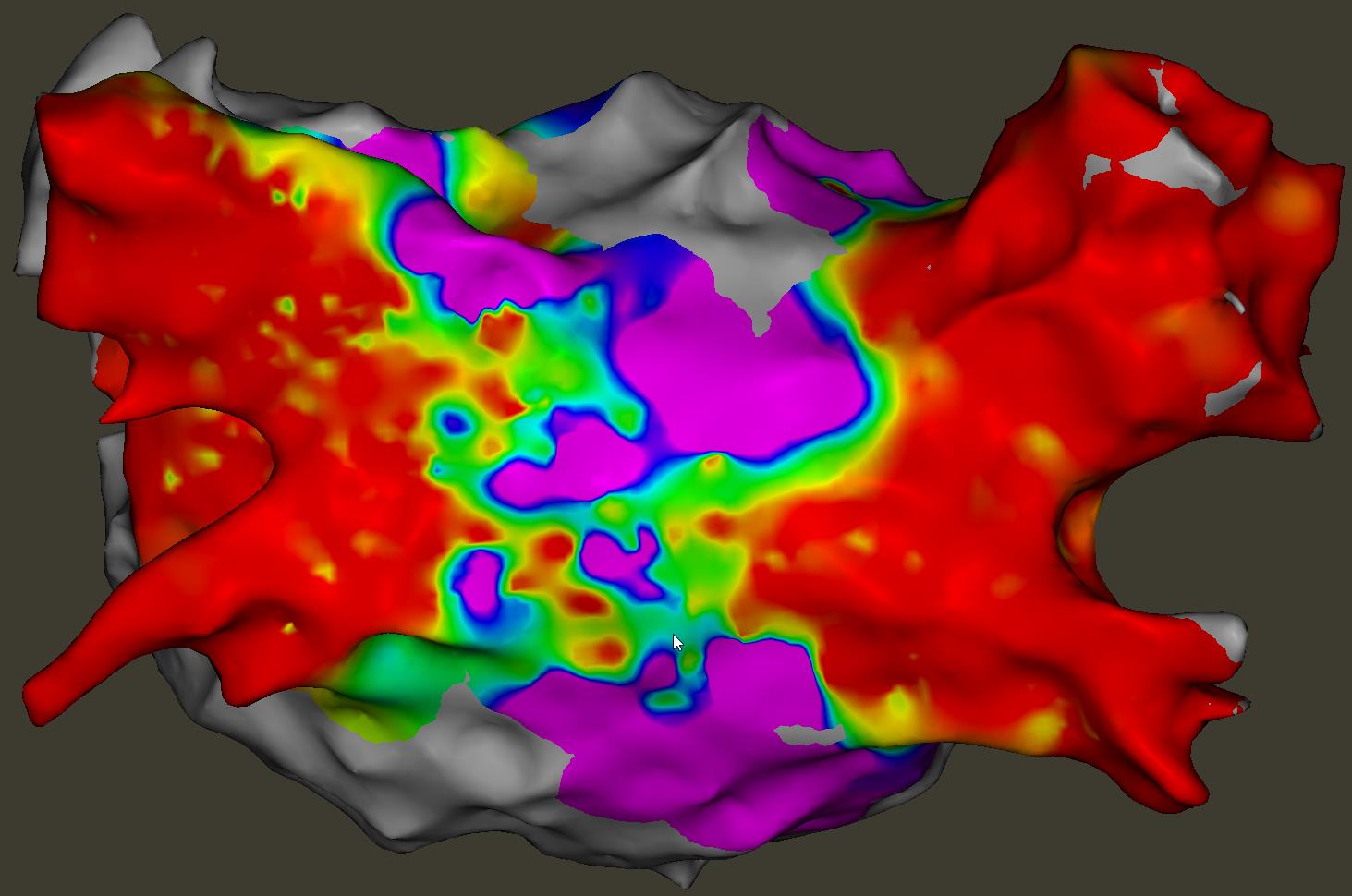

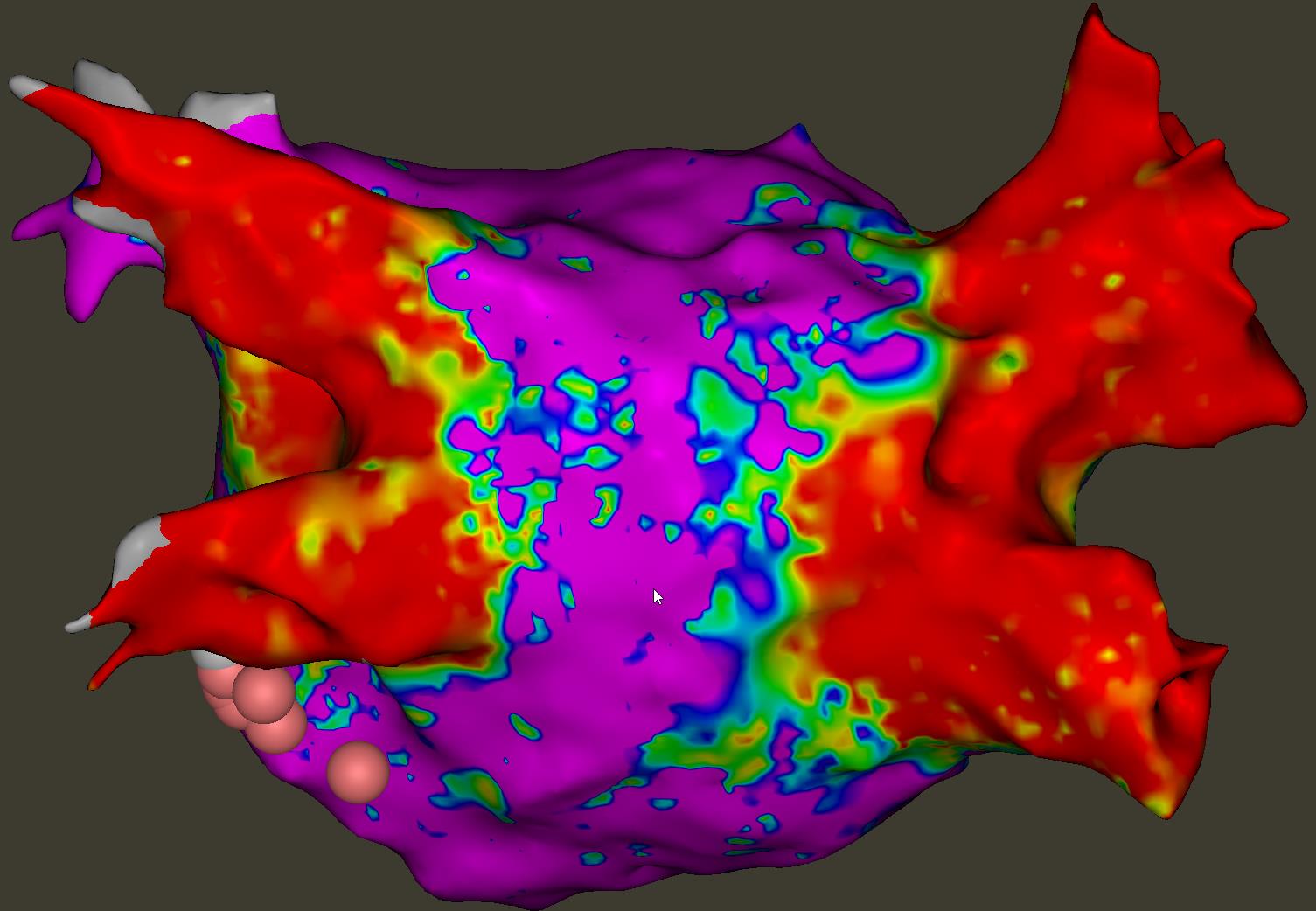


No map acquired


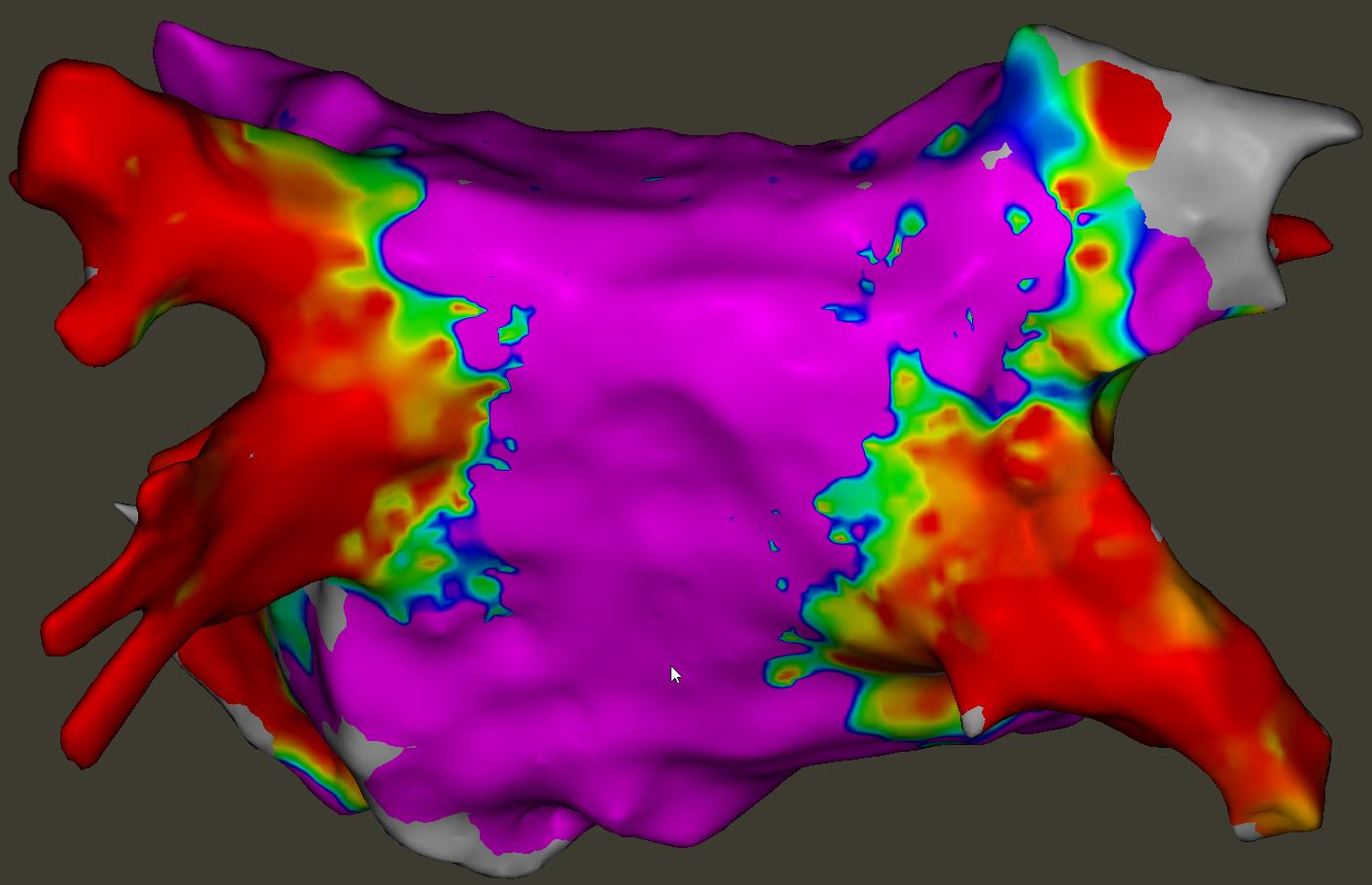


No map acquired


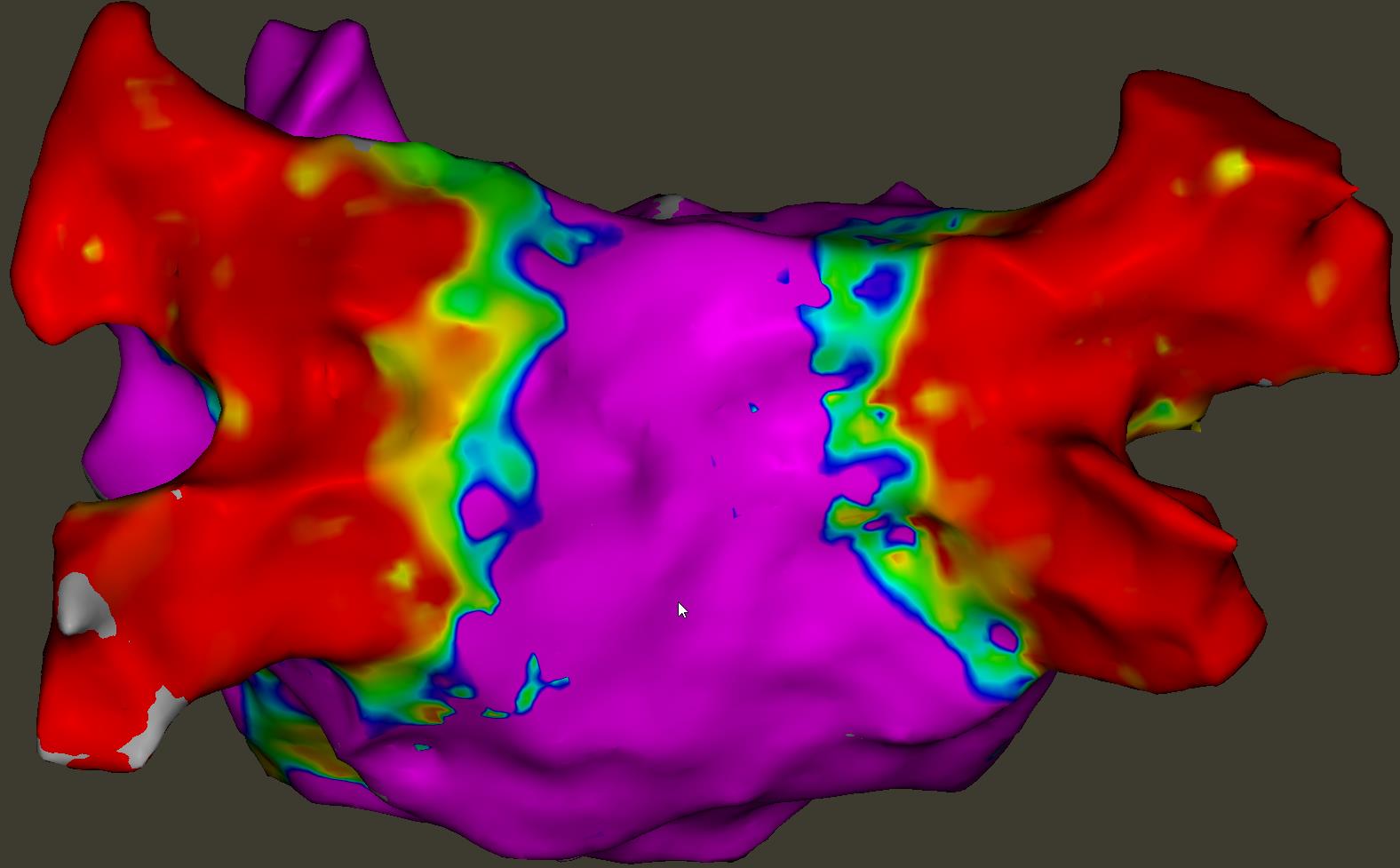

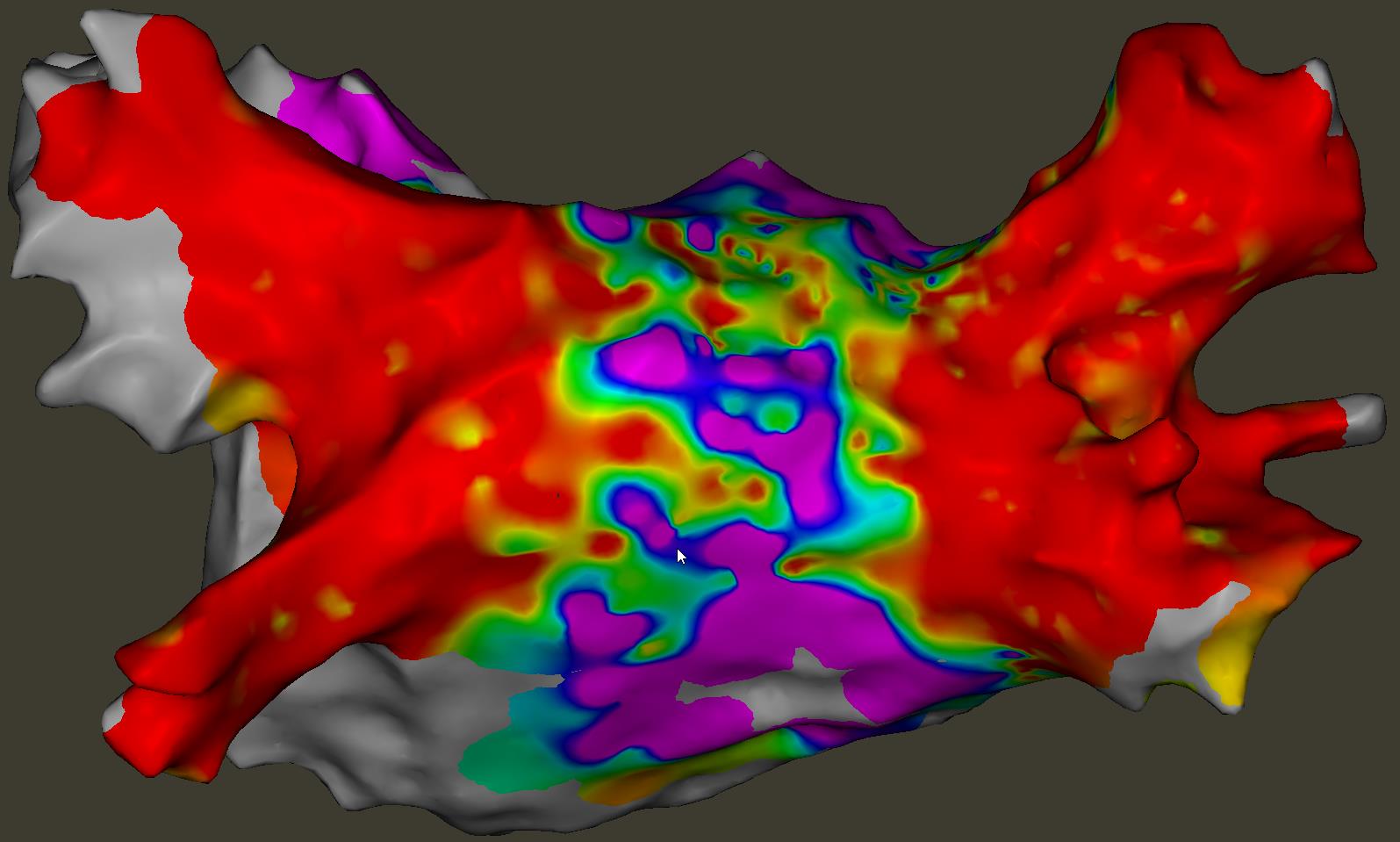

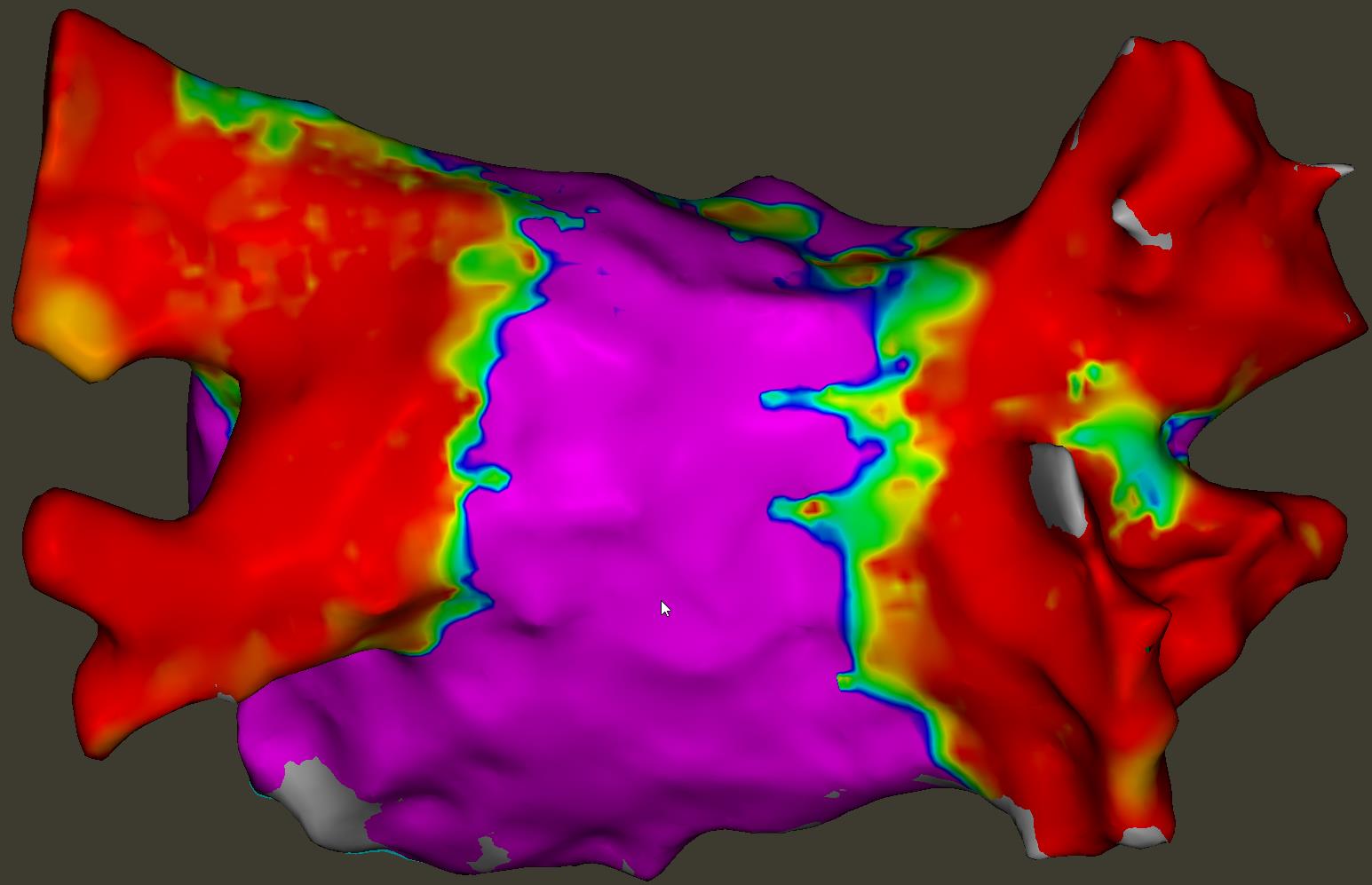

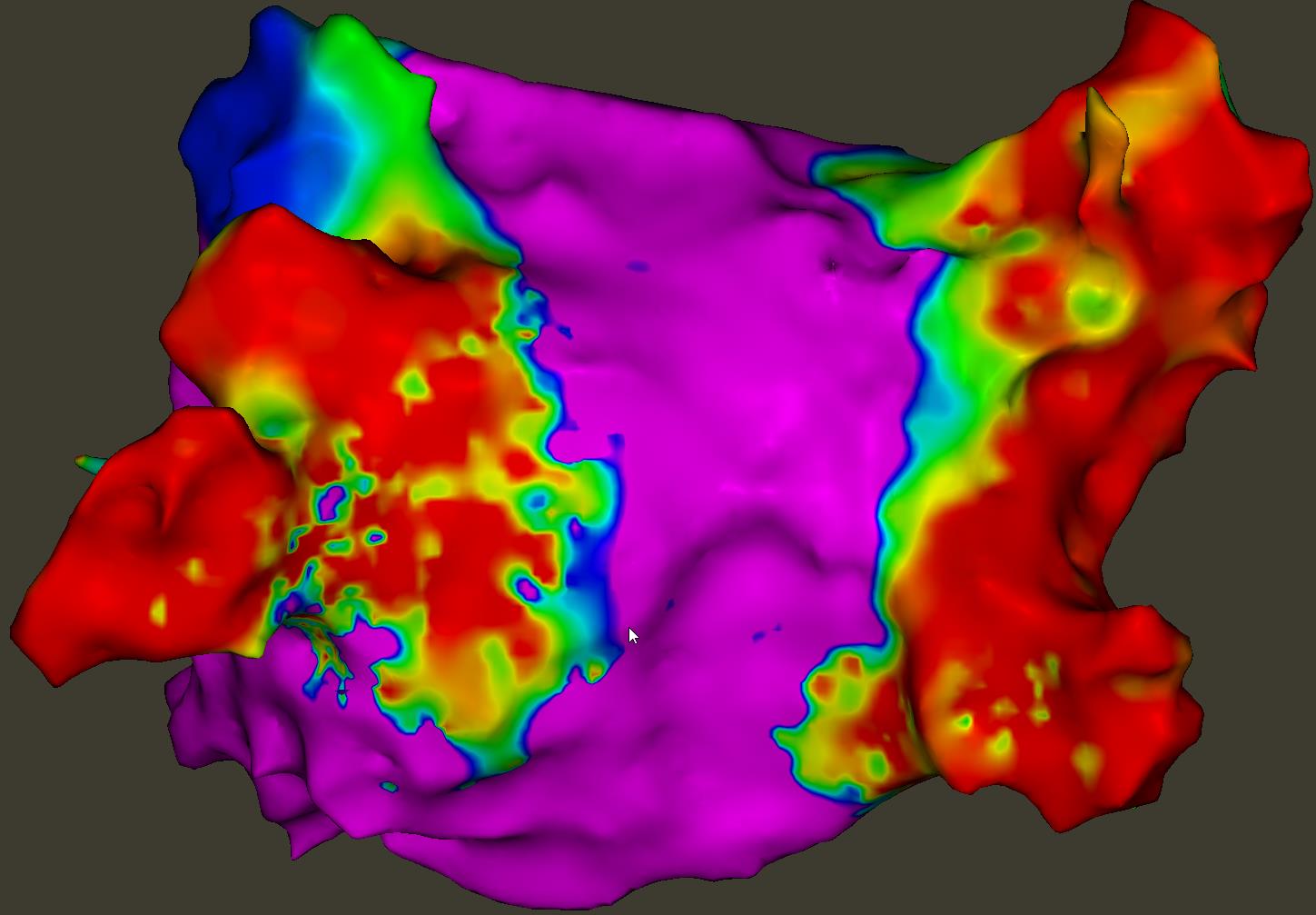

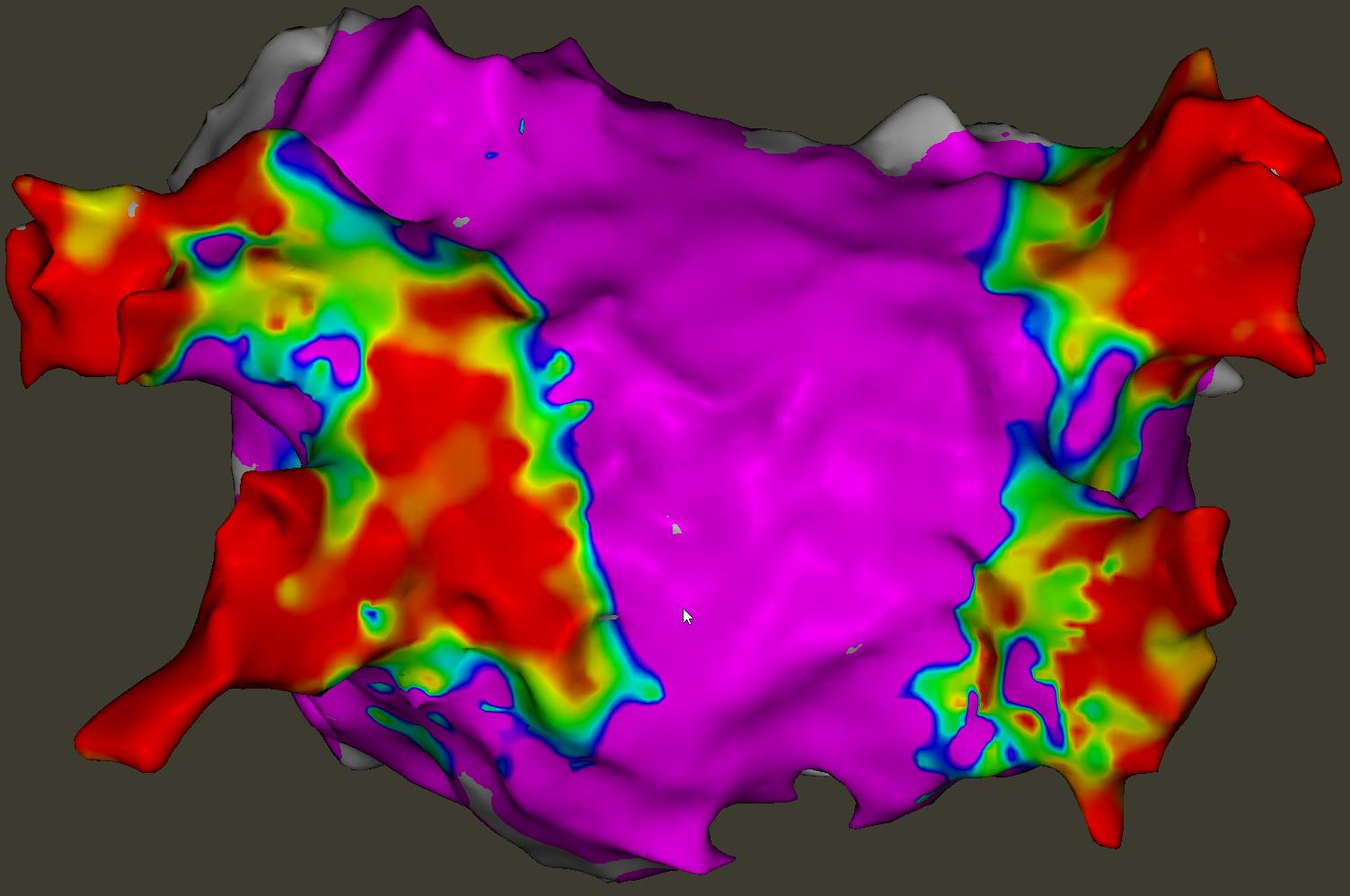

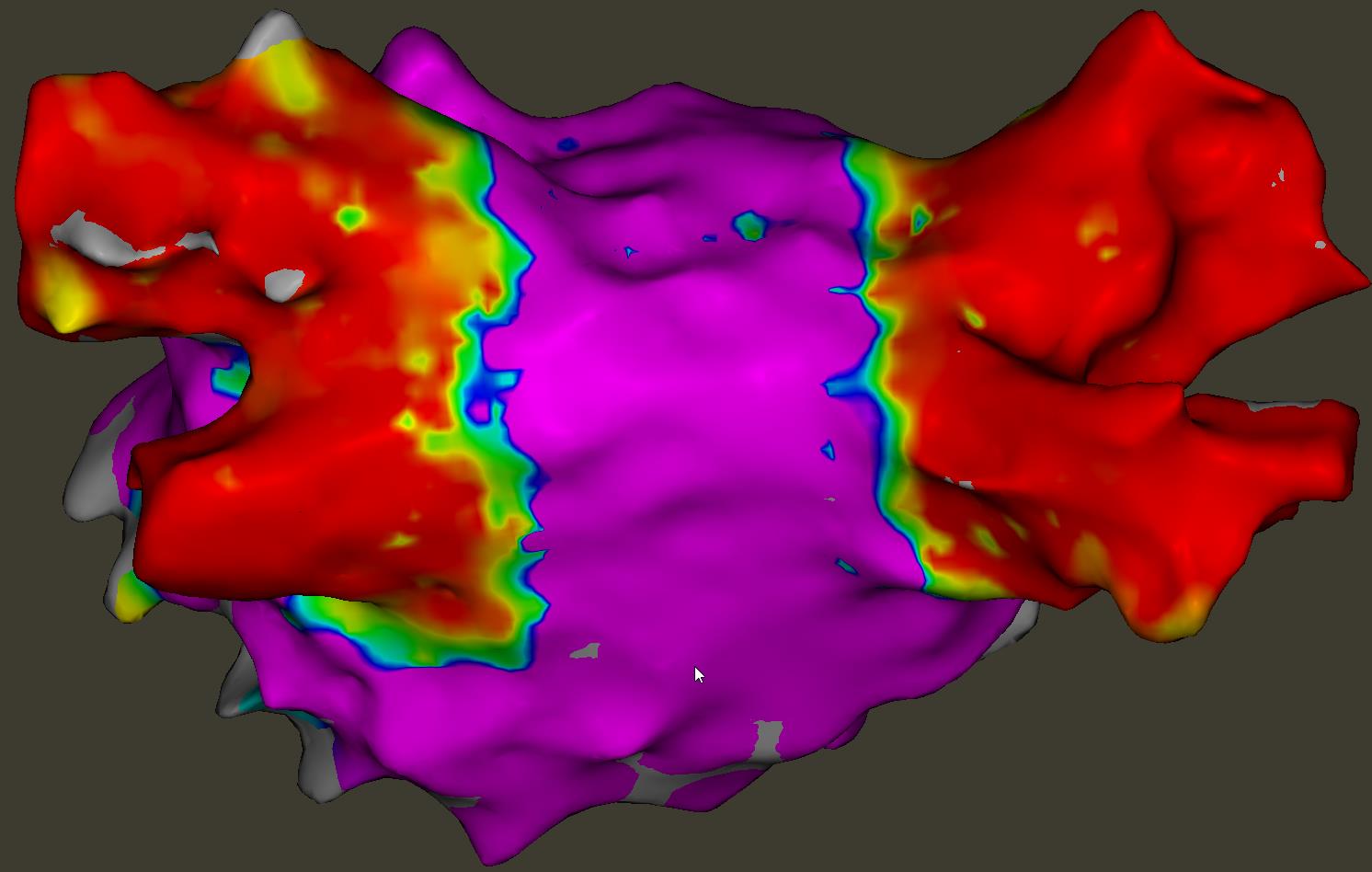

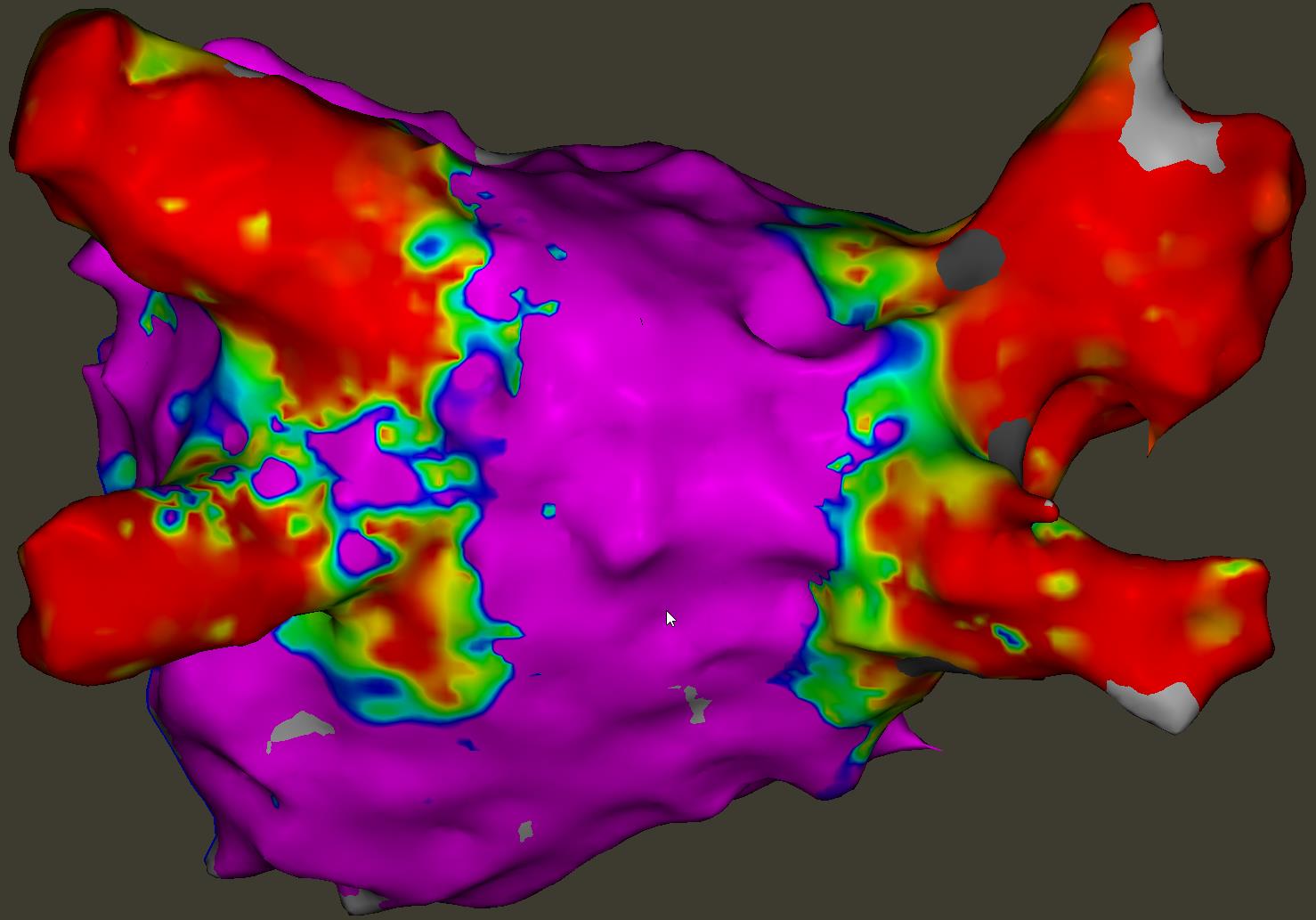

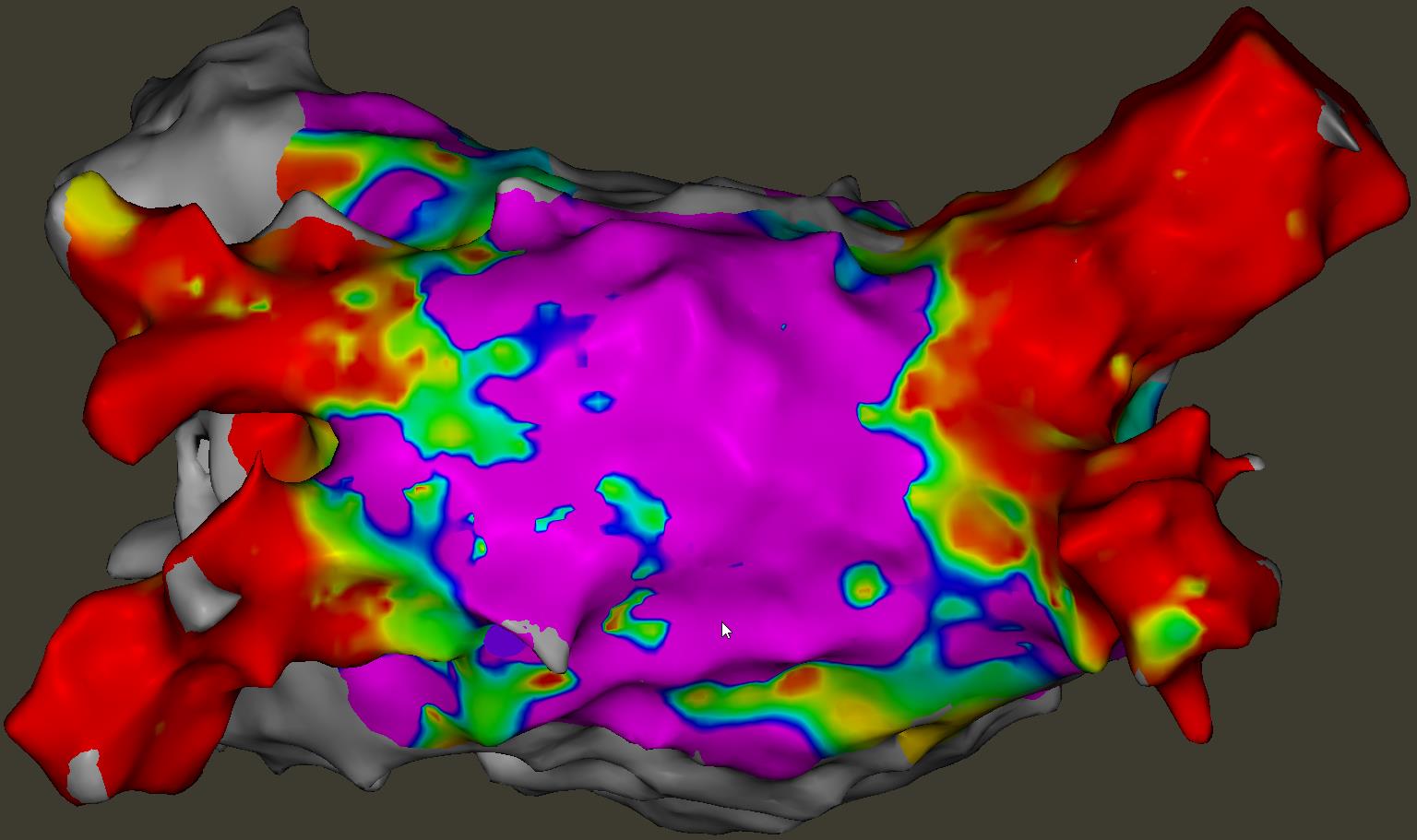

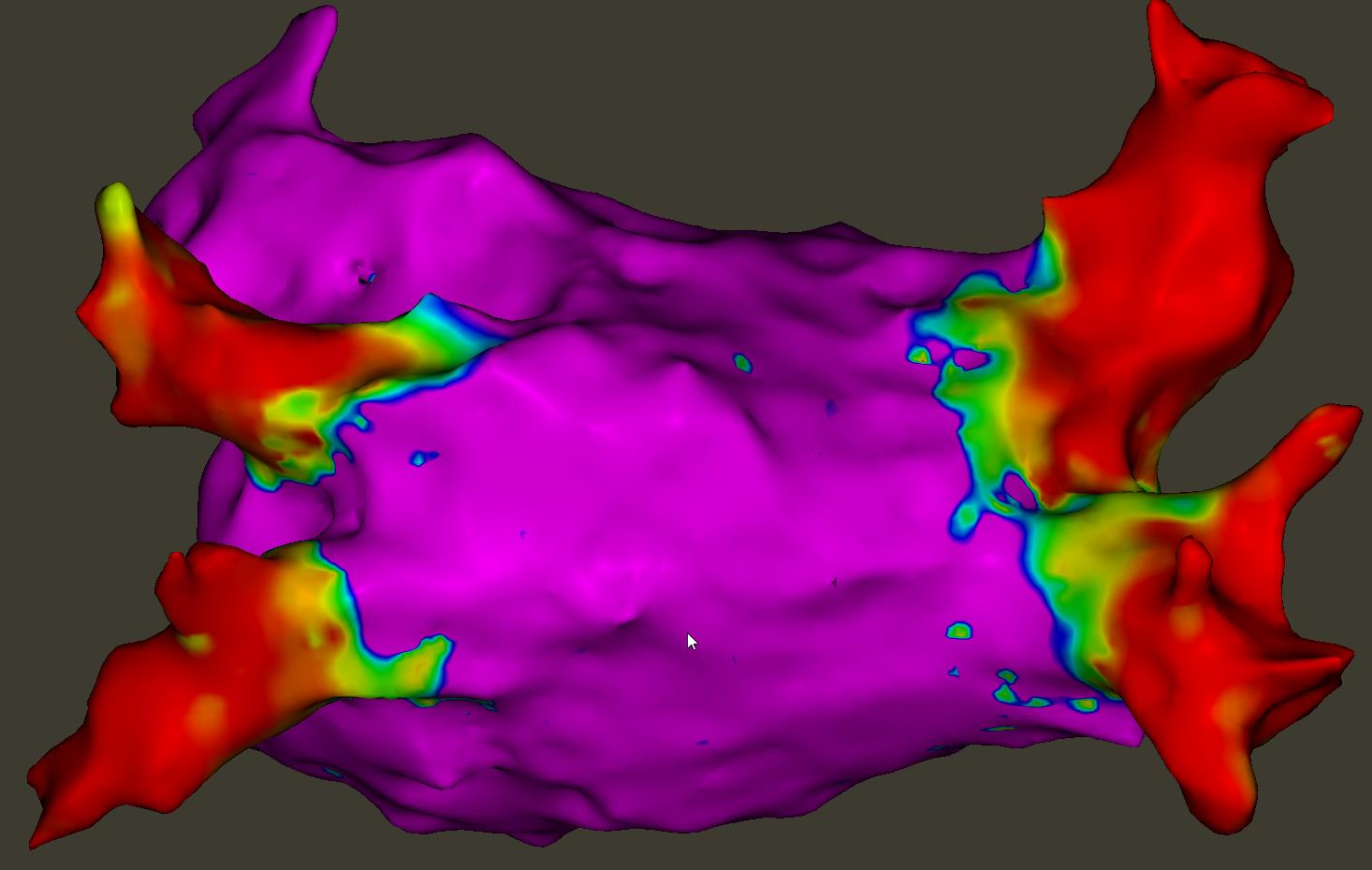

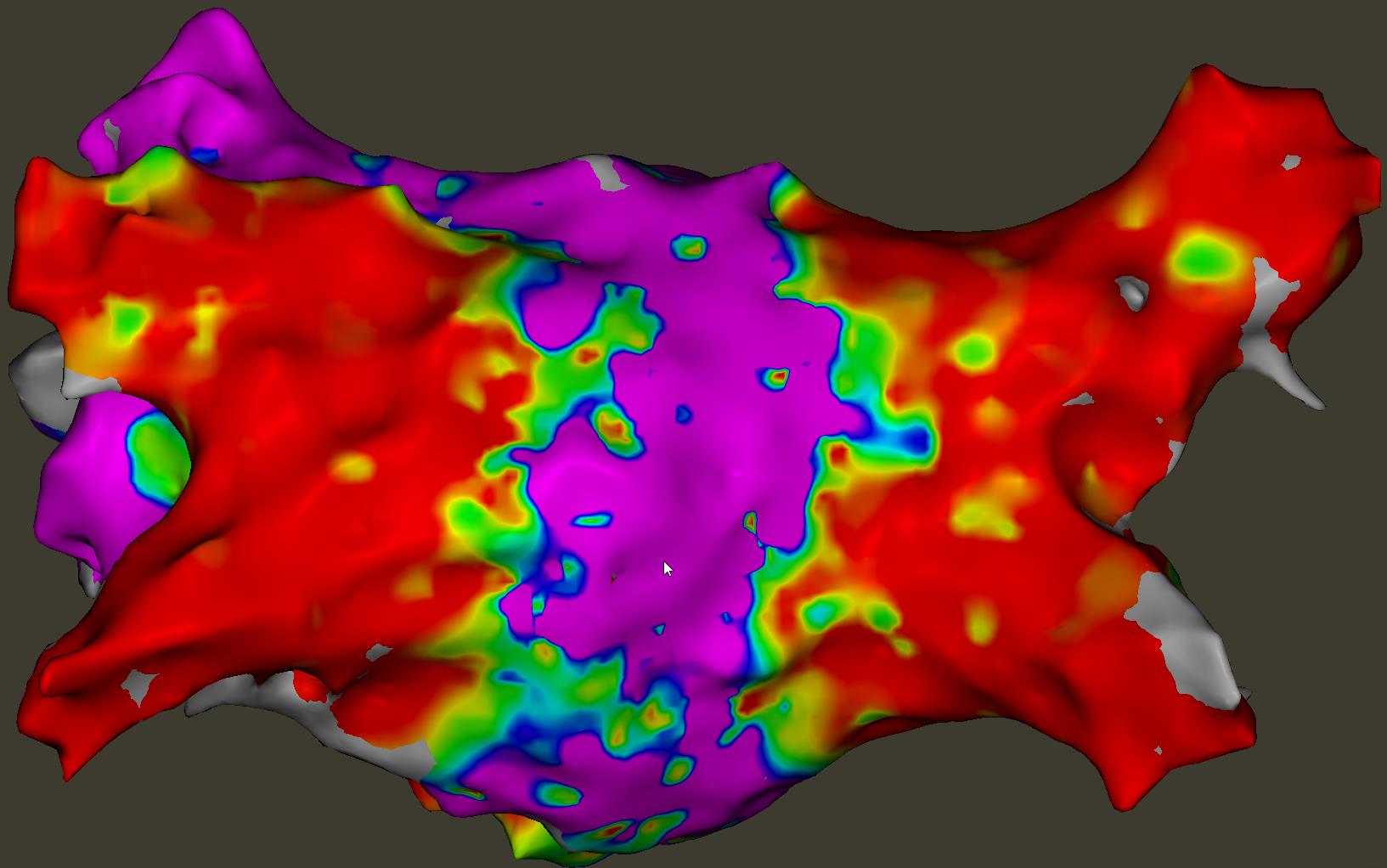

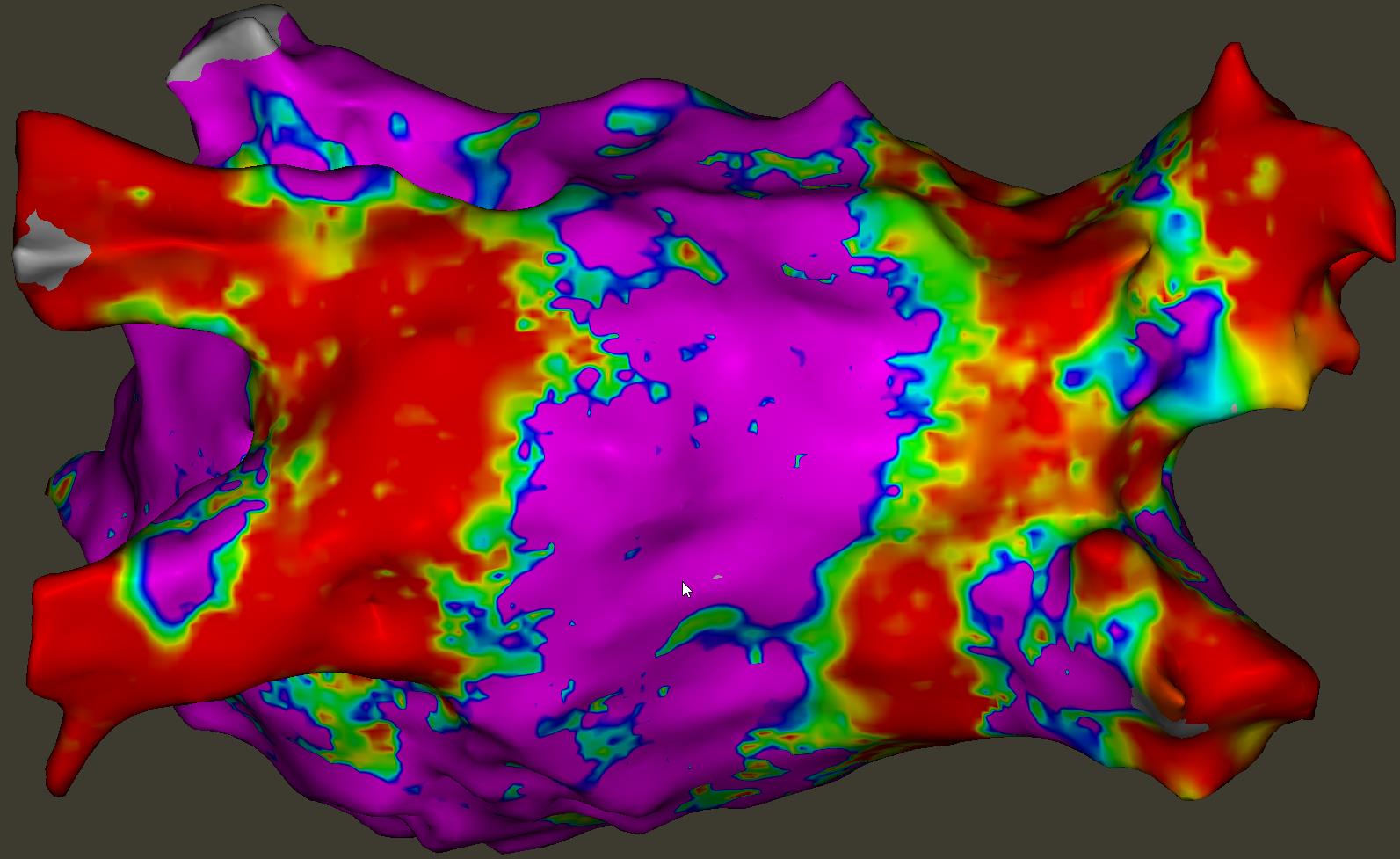

Supplement: Supplementary file 2 — (DOCX 4.91 MB) [file 10840_2023_1608_MOESM2_ESM.docx]
